# DEAD-Box RNA Helicase Family in Physic Nut (*Jatropha curcas* L.): Structural Characterization and Response to Salinity

**DOI:** 10.3390/plants13060905

**Published:** 2024-03-21

**Authors:** Rahisa Helena da Silva, Manassés Daniel da Silva, José Ribamar Costa Ferreira-Neto, Bruna de Brito Souza, Francielly Negreiros de Araújo, Elvia Jéssica da Silva Oliveira, Ana Maria Benko-Iseppon, Antonio Félix da Costa, Éderson Akio Kido

**Affiliations:** 1 Plant Molecular Genetics Laboratory, Genetics Department, Center of Biosciences, Federal University of Pernambuco, Recife CEP 50670-901, PE, Brazil; rahisa.silva@ufpe.br (R.H.d.S.); manasses.dsilva@ufpe.br (M.D.d.S.); bruna.britosouza@ufpe.br (B.d.B.S.); francielly.negreiros@ufpe.br (F.N.d.A.); elvia.oliveira@ufpe.br (E.J.d.S.O.); ederson.kido@ufpe.br (É.A.K.); 2 Plant Genetics and Biotechnology Laboratory, Genetics Department, Center of Biosciences, Federal University of Pernambuco, Recife CEP 50670-901, PE, Brazil; joseribamar.ferreiraneto@ufpe.br; 3 Agronomic Institute of Pernambuco—IPA, Recife CEP 50761-000, PE, Brazil; felix.antonio@ipa.br

**Keywords:** genomics, transcriptomics, abiotic stress, RNA helicase, Euphorbiaceae, oilseed, qPCR

## Abstract

Helicases, motor proteins present in both prokaryotes and eukaryotes, play a direct role in various steps of RNA metabolism. Specifically, SF2 RNA helicases, a subset of the DEAD-box family, are essential players in plant developmental processes and responses to biotic and abiotic stresses. Despite this, information on this family in the physic nut (*Jatropha curcas* L.) remains limited, spanning from structural patterns to stress responses. We identified 79 genes encoding DEAD-box RNA helicases (*Jc*DHX) in the *J. curcas* genome. These genes were further categorized into three subfamilies: DEAD (42 genes), DEAH (30 genes), and DExH/D (seven genes). Characterization of the encoded proteins revealed a remarkable diversity, with observed patterns in domains, motifs, and exon–intron structures suggesting that the DEAH and DExH/D subfamilies in *J. curcas* likely contribute to the overall versatility of the family. Three-dimensional modeling of the candidates showed characteristic hallmarks, highlighting the expected functional performance of these enzymes. The promoter regions of the *Jc*DHX genes revealed potential *cis*-elements such as Dof-type, BBR-BPC, and AP2-ERF, indicating their potential involvement in the response to abiotic stresses. Analysis of RNA-Seq data from the roots of physic nut accessions exposed to 150 mM of NaCl for 3 h showed most of the *Jc*DHX candidates repressed. The protein–protein interaction network indicated that *Jc*DHX proteins occupy central positions, connecting events associated with RNA metabolism. Quantitative PCR analysis validated the expression of nine DEAD-box RNA helicase transcripts, showing significant associations with key components of the stress response, including RNA turnover, ribosome biogenesis, DNA repair, clathrin-mediated vesicular transport, phosphatidyl 3,5-inositol synthesis, and mitochondrial translation. Furthermore, the induced expression of one transcript (*JcDHX44*) was confirmed, suggesting that it is a potential candidate for future functional analyses to better understand its role in salinity stress tolerance. This study represents the first global report on the DEAD-box family of RNA helicases in physic nuts and displays structural characteristics compatible with their functions, likely serving as a critical component of the plant’s response pathways.

## 1. Introduction

The physic nut (*Jatropha curcas* L.), a small, inedible shrub belonging to the Euphorbiaceae family, is notable for its seeds containing a substantial amount of oil. The quality of this oil makes it a promising source for biodiesel generation, presenting a potential solution to the issues associated with fossil fuel use [1]. Moreover, these plants thrive in marginal areas and play a vital role in controlling erosion and revitalizing soils contaminated by heavy metals [2,3].

A primary challenge in modern agriculture is the increasing salinization of soils, which is a critical abiotic stress that causes substantial production loss. Salinity disrupts the ionic and osmotic balance of plants, hindering water and nutrient absorption and ultimately leading to the excessive production of reactive oxygen species (ROS) and damage to membranes, proteins, and organelles [4].

RNA helicases are universal enzymes that use ATP hydrolysis energy to unwind RNA strands and are essential components of various steps of RNA metabolism [5]. Categorized into six superfamilies (SF1-6), the majority fall into SF2, further divided into three subfamilies: DEAD, DEAH, and DExH/D, commonly referred to as DEAD-box, based on variations in the Asp-Glu-Ala-Asp (DEAD) motif [6]. DEAD-box RNA helicases are the largest family of helicases, characterized by the presence of nine conserved motifs (Q, I, Ia, II, III, IV, V, and VI) that are known to be involved in helicase and ATPase activities [5,6]. DEAD-box proteins also contain N- and C-terminal extensions that differ in domain composition and, in some cases, target the proteins to specific substrates via protein–protein interactions [5,6].

Several plant species, including *Arabidopsis thaliana*, *Oryza sativa*, *Gossypium raimondii*, *Solanum lycopersicum*, *Vitis vinifera*, and *Triticum aestivum*, have reported *DHX* genes encoding DEAD-box RNA helicases [7,8,9,10,11,12]. These *DHX* genes play multiple roles in RNA metabolism and have been implicated in responses to environmental stresses. For example, the *BrRH22* gene, which exhibits RNA chaperone activity, has been linked to drought and salinity tolerance in transgenic *A. thaliana* plants [13]. Similarly, *SlDEAD30* and *SlDEAD31*, which are responsive to drought and salinity, conferred tolerance to these abiotic stresses when overexpressed in tomato [14]. Overexpression of the *AvDH1* gene in cotton provided salinity tolerance and reduced oxidative stress [15], whereas overexpression of the *OsRH58* gene enhanced salinity and drought tolerance in transgenic *A. thaliana* plants [16].

Understanding plant responses to abiotic stresses is crucial, given the anticipated increase in the frequency and intensity of environmental stresses due to ongoing climate change. This poses a threat to food security in several populations in addition to biodiversity loss. In this study, the *J. curcas* DEAD-box RNA helicase family was comprehensively analyzed, including characterization of exon–intron structure patterns, conserved domains and motifs, potential secondary structures, and 3D models of DEAD-box subfamily candidates. Additionally, this study explored the promoter regions of related genes for the presence of cis-regulatory elements (CREs) and evaluated the responses of *DHX* transcripts expressed in *J. curcas* plants after three hours of exposure to NaCl (150 mM). This study marks the first genome-wide report of the DEAD-box RNA helicase family in *J. curcas*, offering insights that may contribute to future investigations of this gene family in related species, particularly those with current breeding programs for developing plant tolerance to abiotic stresses.

## 2. Material and Methods

### 2.1. Identification of Putative JcDHX Proteins and Genes

The identification of *JcDHX* (*Jatropha curcas DHX* genes) potential proteins commenced with a comprehensive exploration of the physic nut proteome (RJC1_Hi-C_protein.faa) associated with the *J. curcas* reference genome (NCBI RefSeq assembly GCF_014843425.1, Genome assembly RJC1_Hi-C: 282,312 Mb, 22,718 genes, 29,586 proteins), accessible on the NCBI website (https://www.ncbi.nlm.nih.gov (accessed on 25 November 2020)). The Hidden Markov Model (HMM) played a pivotal role in this process, employing the HMM DEAD profile (PF00270) sourced from the Pfam protein family database, according to InterPro v97.0 (https://www.ebi.ac.uk/interpro/ (accessed on 5 April 2021); Ref. [17]). Standard parameters and an e-value cut-off of <$1.0^{-5}$ were applied for domain annotation of each protein sequence. Furthermore, the *JcDHX* candidates were scrutinized through CDD v3.13 (Conserved Domain Database—https://www.ncbi.nlm.nih.gov/Structure/cdd/wrpsb.cgi (accessed on 7 April 2021)) [18] and SMART v9 (Simple Modular Architecture Research Tool—http://smart.embl-heidelberg.de/ (accessed on 20 April 2021)) [19]. This dual-validation approach bolstered the identification of conserved domains within candidate proteins. Putative *Jc*DHX proteins were associated with genes in the *J. curcas* reference genome.

### 2.2. Promoter Analysis

To explore the regulatory landscape of *JcDHX* genes, we retrieved the promoter regions situated 1000 base pairs upstream of the transcription initiation site from the NCBI Genome Browser panel (https://www.ncbi.nlm.nih.gov/genome/gdv/browser/ (accessed on 9 July 2021)) of the *J. curcas* reference genome (Genome assembly RJC1_Hi-C). Subsequently, each promoter region was analyzed using the MEME v5.3.3 program (https://meme-suite.org/meme/ (accessed on 2 August 2021)) [20]. For each identified motif, the software reported the corresponding e-value. Motifs with an e-value < 0.05 are considered significant. The maximum number of motifs analyzed in this study for a single *JcDHX* promoter region was 10, and the extension ranged from 6 to 50 nt. To identify shared motifs and gain insights into their significance, the Tomtom software v5.3.3 (https://meme-suite.org/meme/tools/tomtom (accessed on 2 August 2021)) [21], in conjunction with the JASPAR database (JASPAR2018_CORE_plants_non-redundant), was employed. This collaborative approach aimed to recover the identity of the identified motifs, emphasizing those most representative, with a *p*-value cut-off of <$10^{-2}$.

### 2.3. Structural Characterization of the JcDHX Candidates and Potential Subcellular Localization

The prediction of exon–intron structures for the genes encoding candidate *Jc*DHX proteins involved a comparative analysis of the coding sequence (CDS) with the genomic sequence. This was accomplished using the Gene Structure Display Server v2.0 (GSDS, http://gsds.gao-lab.org/ (accessed on 10 November 2021)) [22]. Simultaneously, the physicochemical attributes of the candidate *Jc*DHX proteins were systematically characterized using the ExPASy tool (http://web.expasy.org/protparam/ (accessed on 28 April 2021)) [23]. Concurrently, predictions regarding subcellular localization were made using the CELLO v2.5 tool (http://cello.life.nctu.edu.tw/ (accessed on 27 December 2021)) [24]. To unveil potentially conserved motifs within the sequences, the MEME v5.3.3 program (https://meme-suite.org/meme/ (accessed on 7 July 2021)) [20] was employed. This analysis adhered to the following specific parameters: anr (any number of repetitions), a maximum number of motifs set at 10, and a motif size spanning 6–50 amino acid residues.

### 2.4. Phenetic Analysis and Orthology

To classify *Jc*DHX, a phenetic analysis was conducted, encompassing DHX protein sequences sourced from *S. lycopersicum* (63) and *A. thaliana* (69). These annotated sequences were obtained from the Sol Genomics Network (https://solgenomics.net/ (accessed on 23 July 2021)) and The *Arabidopsis* Information Resource (TAIR; https://www.arabidopsis.org/ (accessed on 29 July 2021)), respectively. Alignment of the protein sequences was performed using the ClustalX v2.1 program [25], and subsequent clustering was achieved using the Neighbor-Joining method with bootstrap analysis comprising 1000 replicates. The resulting clusters were visualized using the online tool iTOL v5 (https://itol.embl.de/ (accessed on 29 March 2022)) [26]. To scrutinize the conservation of the *Jc*DHX family across different species, orthologs were inferred using the Bidirectional Best-Hit (BBH) method [27]. The analysis encompassed species from the Euphorbiaceae family, namely *Manihot esculenta* (GCF_001659605.1; Manihot_esculenta_v6_protein.faa), *Ricinus communis* (GCF_000151685.1; JCVI_RCG_1.1_protein.faa), and *Hevea brasiliensis* (GCF_001654055.1; ASM1654 05v1_protein.faa). Additionally, representatives of *Populus trichocarpa* (GCF_000002775.4; Pop_tri_v3_protein.faa), *S. lycopersicum* (GCF_000188115.4; SL3.0_protein.faa), and *A. thaliana* (Araport11_genes.201606.pep.fasta), were included in the analysis to provide a broader comparative context.

### 2.5. Secondary Structure Elements and 3D Modeling

We performed a secondary structural analysis using the SOPMA method (https://npsa-prabi.ibcp.fr/cgi-bin/npsa_automat.pl?page=/NPSA/npsa_sopma.html (accessed on 23 October 2023)) to predict secondary structure elements (SSEs) [28]. Multiple alignment of the *Jc*DHX DEAD subfamily candidates was conducted using ClustaX v2.1 [25] and viewed using the Jalview v.2.11.3.2 software [29]. This alignment was used as an input file to generate a 2D alignment with the Ali2D tool (https://toolkit.tuebingen.mpg.de/tools/ali2d (accessed on 9 September 2023)) [30], followed by graphical visualization provided by the 2dSS tool (http://genome.lcqb.upmc.fr/2dss/ (accessed on 10 September 2023)) [31].

Homology modeling of the *Jc*DHX candidates hinged upon the structure of the most similar PDB templates, facilitated by the SWISS-MODEL web tool (https://swissmodel.expasy.org/ (accessed on 22 September 2023)) [32]. Subsequent scrutiny and adjustments of the generated 3D models were performed using the PyMOL v2.5 program (https://pymol.org/ (accessed on 1 September 2023)) [33]. Model accuracy was assessed using Ramachandran plots.

### 2.6. RNA-Seq Analysis of JcDHX Candidates and Gene Expression Validation by qPCR

We performed an in silico analysis of *Jc*DHX expression using RNA-Seq data from two distinct Brazilian accessions of *J. curcas* that displayed different NaCl tolerance phenotypes, with *Jc*183 manifesting tolerance and *Jc*171 exhibiting a less tolerant phenotype [34]. The transcriptome data used were previously sequenced and analyzed by our group [35]. The experimental salt assay followed a completely randomized design with two accessions, two treatments (without salt or with NaCl, 150 mM, three-hour salt exposure), and three plants (half-siblings) of each accession simulating biological replicates. After salt exposure, we collected the roots, which were immediately frozen in liquid nitrogen and stored (−80 °C) until RNA extraction. A total of 12 RNA-Seq libraries (two accessions × two treatments × three plants each accession) were generated following the LS protocol of the Illumina TruSeq Stranded mRNA Sample Prep kit (Illumina, Inc., San Diego, CA, USA). Libraries were sequenced on an Illumina HiSeq 2500 (paired-end 100 bp reads). The de novo transcriptome covered 101 MB and 145,422 assembled transcripts with a GC content of 41.55%, and the N50 reached 1308 bp. The average aligned fraction was 0.97, with 84,534 transcripts with at least one significant alignment to the reference genome (GenBank assembly accession number: GCA_000696525.1) [35]. Comprehensive details regarding the salinity assay, RNA extraction, RNA-Seq libraries, and transcriptome assembly procedures are outlined by Souza et al. [35]. In order to identify DHX candidates in this transcriptome, RNA-Seq transcripts were subjected to BLASTx analysis (e-value cut-off: $e^{-10}$), aligning them with previously identified *Jc*DHX proteins. Each transcript sequence had its domains confirmed using the CDD [18] and SMART [19] tools. TransDecoder (https://github.com/TransDecoder/TransDecoder (accessed on 17 August 2021)) [36] was used to obtain peptide sequences from *Jc*DHX RNA-Seq transcripts. RNAsamba (https://rnasamba.lge.ibi.unicamp.br/ (accessed on 6 February 2024)) [37] was used to describe the coding probabilities of identified transcripts. To evaluate whether RNAs classified as non-coding by RNAsamba could be assigned to protein families, we compared transcripts translated to a UniProtKB/Swiss-Prot from *Arabidopsis thaliana* using BLASTp (e-value cut-off: $e^{-10}$) [38]. Finally, the translated ORFs were compared to the Pfam protein family database (version 32.0) using the hmmsearch command from the HMMER suite. Transcripts not translated by TransDecoder had open reading frames (ORFs) predicted by ORF Finder (https://www.ncbi.nlm.nih.gov/orffinder/ (accessed on 7 February 2024)) [39]. *Jc*DHX candidates identified as differentially expressed transcripts (DETs), representing the Trinity unigenes (unique assembled transcripts), exhibited a *p*-value < 0.0001 and false discovery rate (FDR) < 0.005 for de novo transcriptome assembly. The fold change (FC) values discriminated positively (UR, upregulated; FC ≥ 1) from the negative modulation of expression (DR, downregulated; FC ≤ −1). FC values represent the ratio of transcript abundance considering their presence in two compared RNA-Seq libraries with the untreated library as the reference sample. Hierarchical clustering of the *Jc*DHX transcript, based on the Cluster v3.0 software (https://cluster2.software.informer.com/3.0/ (accessed on 11 March 2024)), considered the FC values, and the visualization was performed using the Java TreeView v1.1 software [40].

For the validation of the in silico expression of selected DETs, we designed primers for qPCR assays using the Primer3 tool [41], with some adjustments for amplicon size (70–200 bp), GC content (45–55%), dissociation temperature (50–80 °C), and CG clamp (one). The specificity of amplicons was assessed using dissociation curves generated between 65 and 95 °C (20 min) after 40 qPCR cycles. To ensure robust quantification, the amplification efficiency [42] for each primer pair was determined using calibration curves established with cDNAs from the respective accessions in serial dilutions (no dilution, 10-1, 10-2, 10-3, and 10-4). qPCR reactions were conducted in a LineGene 9660 thermocycler (Bioer^®^, Hangzhou, China) using SYBR Green as the detection system. The final reaction volume was 10 µL, comprising 1 µL of sample cDNA (1:10), 5 µL of GoTaq^®^ qPCR Master Mix (Promega®, Madison, WI, USA), 2 µL of ultrapure water, and 1 µL of each primer (0.05 µM). The qPCR reactions were executed in technical and biological triplicates, featuring a negative control, and included two reference genes (actin and β-tubulin). These reference genes were selected through specific assays using samples from the same experimental set. The reaction protocol included an initial denaturation step at 95 °C (2 min), followed by 40 cycles of 95 °C (15 s) and 60 °C (60 s). Quantification cycles (Cq), dissociation temperature, and both absolute and relative quantification were determined using the Bioer^®^ proprietary software. Expression data were further analyzed using the REST software (v. 2.0.13) [43].

### 2.7. Protein–Protein Interaction (PPI) Networks for JcDHX Candidates

To explore the potential interactions of *Jc*DHX proteins codified by DETs, we used the STRING software (v11.5) [44] to construct PPI networks. A confidence score of >0.7 (high) was set for interaction, relying on experiment-derived interactions in *Arabidopsis*. Text-mining-based evidence was excluded to ensure reliability of the predicted PPI networks. Cytoscape v3.10.1 [45] was used to visualize and editing protein–protein interaction (PPI) networks.

## 3. Results

### 3.1. Identification of JcDHX Genes and Their Gene Structures

The *J. curcas* reference genome (GCF_014843425.1_RJC1_Hi-C) contains 79 genes encoding 146 DHX proteins. *DHX* genes, designated *JcDHX1* to *JcDHX79* based on their respective scaffold positions, are shown in Table S1, with details including the genomic location, locus ID, gene length (nt), intron count, ORF length (nt), expected protein length (aa), and DHX subfamily.

**Figure 1:**
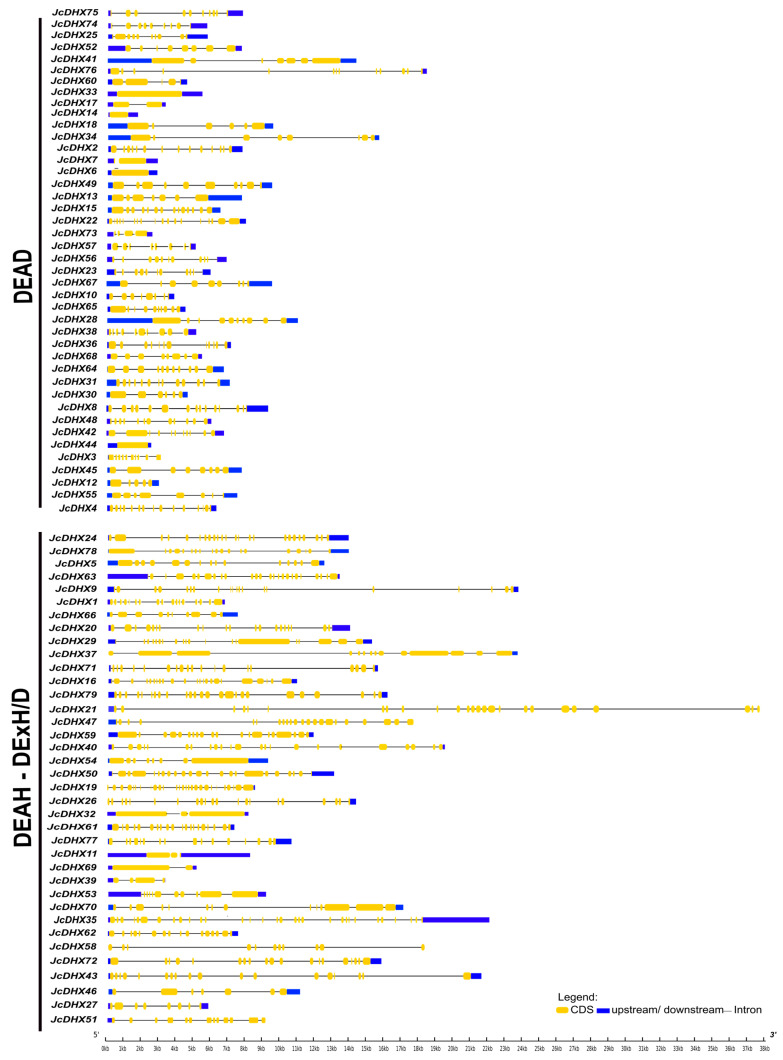
Exon–intron structures of *JcDHX* genes. The graphical representation was generated using the Gene Structure Display (GSDS).

Gene structure is a critical factor for understanding the evolution of gene families. The exon–intron structures of *JcDHX* genes, comprising 42 DEAD and 37 DEAH/DExH/D subfamily members, are shown in Figure 1. Our analysis revealed a lack of discernible patterns within each subfamily and considerable variability even among members of the same subfamily (Figure 1). The intron investigation revealed a broad range of variation, detecting no introns to a maximum of 30 introns (Table S1). In general, DEAD subfamily members exhibited simpler gene structures, with sizes ranging from approximately 1500 nt to almost 18,000 nt, and a reduced number of introns, including those without introns (e.g., *JcDHX6*, *JcDHX44*, *JcDHX33*, and *JcDHX14*; Table S1 and Figure 1). Conversely, DEAH and DExH/D subfamily members exhibited greater lengths, spanning approximately 3200 nt to almost 39,000 nt, and more complex gene structures, with a potential for up to 30 introns (Table S1 and Figure 1). This comprehensive exploration of the *JcDHX* gene family provides valuable insights into structural diversity and sheds light on the distinct characteristics within and between subfamilies.

### 3.2. Analysis of JcDHX Gene Promoter Regions

**Figure 2:**
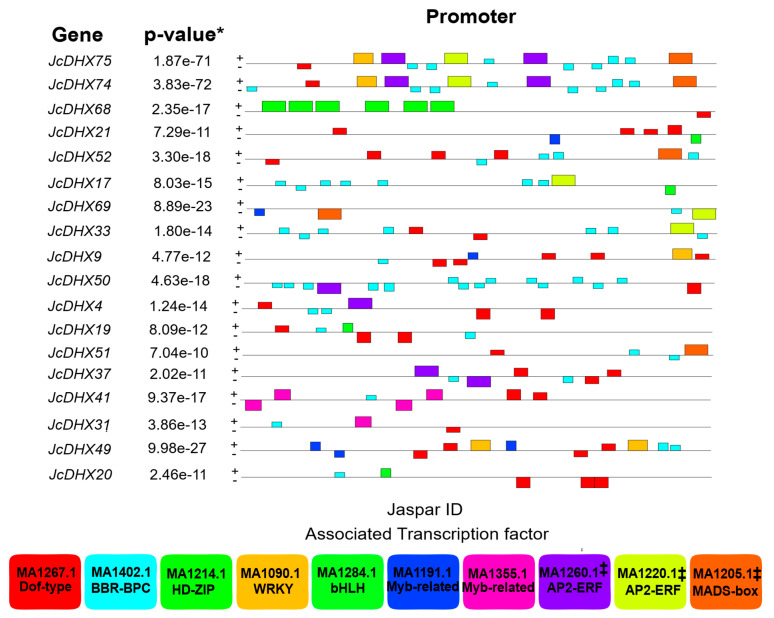
Distribution of candidate cis-regulatory elements (CREs) in the promoter set of *Jatropha curcas* DHX genes. The colored boxes present information about the CREs and the associated transcription factors with JASPAR IDs. MEME’s combined *p*-value *, representing the probability of a random sequence matching the motif under test with a score greater than or equal to that found in the sequence under test; ‡ statistical significance below the considered cut-off (e-value < 0.05; *p*-value < 0.01).

Analysis of *cis*-regulatory elements (CREs) in promoter regions (1.0 kb) of the 79 putative *JcDHX* genes of the *J. curcas* RJC1_Hi-C genome identified potential TFs possibly interacting with these genes. Eight motifs were detected using the MEME program (e-value < 0.05). Seven of the top eight detected CRE motifs (*p*-value < $10^{-2}$) were associated with TF members of the Dof-type, BBR-BPC, HD-ZIP, WRKY, Myb-related, and bHLH families (Figure 2; Table S2). For each enriched CRE motif, details such as the MEME logo, JASPAR IDs, and e-values are shown in Figure S1. CREs distributed along the promoter regions are shown in Figure S2. Notably, the most prevalent TFs associated with the analyzed gene promoters belonged to the Dof-type and BBR-BPC families. Members of these TF families are key regulatory players involved in various cellular processes, including those involving plants’ abiotic stress responses. This comprehensive examination enhances our understanding of the regulatory elements governing the expression of *JcDHX* genes and provides valuable insights concerning their potential assistance in plant responses to stress.

### 3.3. Orthology Analysis of JcDHX Genes

The orthology analysis provided insights into the evolutionary relationships of the *DHX* gene family across the plant species analyzed. The orthology analysis, encompassing bidirectional comparisons of the 79 *JcDHX* genes with sequences of each examined species, according to the BBH methodology, identified substantial ortholog amounts: 74 (with *M. esculenta*), 74 (with *H. brasiliensis*), 73 (with *R. communis*), 72 (with *P. trichocarpa*), 74 (with *S. lycopersicum*), and 70 (with *A. thaliana*). The three Euphorbiaceae species (*M. esculenta*, *R. communis*, and *H. brasiliensis*) shared orthologs of 73 *JcDHX* genes. In turn, three species outside the Euphorbiaceae family (*P. trichocarpa*, *S. lycopersicum*, and *A. thaliana*) shared orthologs of 70 *JcDHX* genes. Across all six analyzed species, shared orthologs of the 69 *JcDHX* genes were identified (Table S3 and Figure S3). Interestingly, no orthologs of five *JcDHX* genes were detected in the six analyzed species. The genes without detected orthologs were *JcDHX11*, *JcDHX14*, *JcDHX34*, and *JcDHX75* (from the DEAH subfamily), as well as *JcDHX58* (DExH subfamily) (Table S3).

### 3.4. Phenetic Analysis of JcDHX Proteins

**Figure 3:**
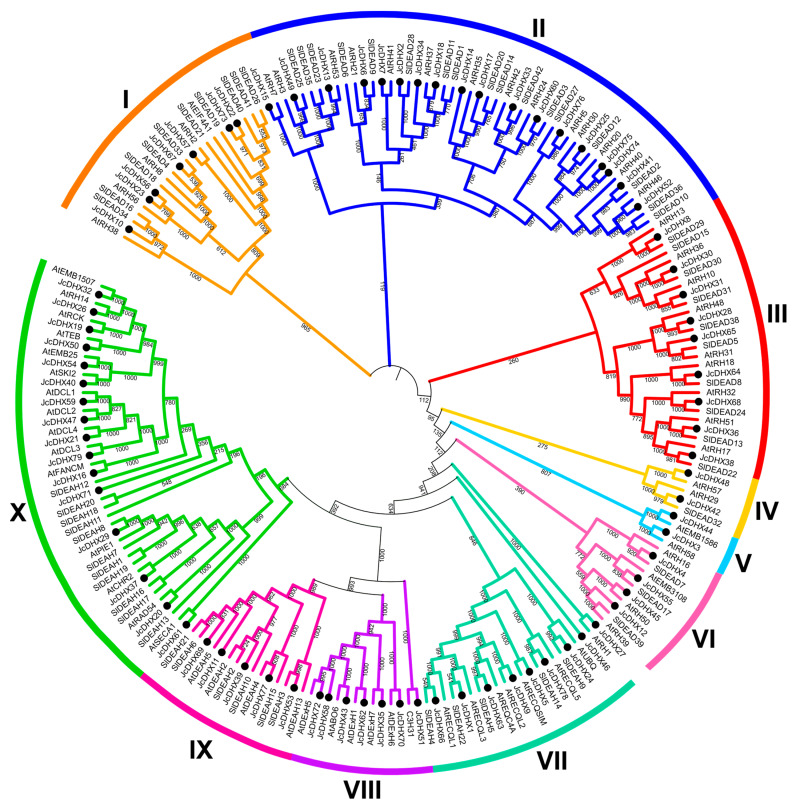
Phenetic tree generated by applying the Neighbor-Joining method (bootstrap of 1000 replicates), considering DEAD-box sequences from *Jatropha curcas*, *Arabidopsis thaliana*, and *Solanum lycopersicum*. Subgroups I to VI correspond to the DEAD subfamily; subgroups VII, IX, and X represent the DEAH subfamily, while subgroup VIII corresponds to DExH/D.

DEAD-box helicases are primarily categorized into subfamilies (DEAD, DEAH, and DExH/D) based on variations in motif II (D-E-A-D). To enhance the classification of potential *Jc*DHX proteins into subfamilies, we conducted a phenetic analysis, covering the largest protein translated from each *JcDHX* gene together with curated DEAD-box RNA helicases from *A. thaliana* and *S. lycopersicum* [7,10]. The resulting phenetic tree grouped *Jc*DHX proteins into ten distinct subgroups (Figure 3).

Evaluation of sequences covering motif II revealed that 42 *Jc*DHX proteins from subgroups I, II, III, IV, V, and VI (Figure 3) comprised the DEAD subfamily, whereas subgroups VII, IX, and X (Figure 3) were assigned to the DEAH subfamily (30 members), and subgroup VIII comprised the DExH/D subfamily (seven members) (Figure 3). This comprehensive analysis supports the validity of the adopted classification for *Jc*DHX proteins.

### 3.5. Conserved Domains and Motifs in JcDHX Proteins

Conserved domains and motifs are crucial for defining the functional specificity of a protein. Among the 146 putative DHX proteins, the structural core of the family, comprising the DEAD-box (N-terminal) and Helicase_C (C-terminal) domains, was observed. In essence, *Jc*DHX proteins have a conserved helicase core essential for their anticipated functions.

**Figure 4:**
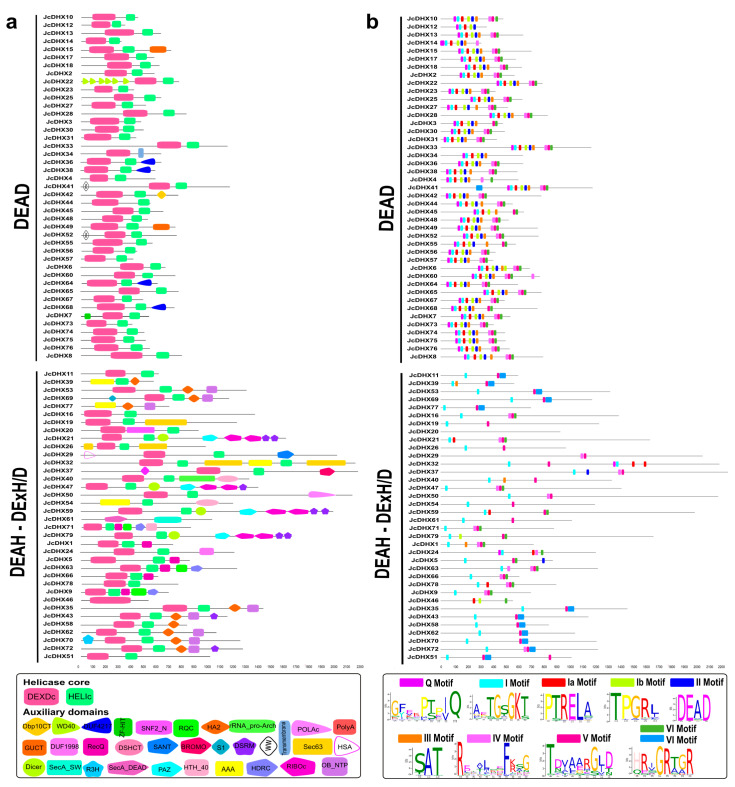
Structure of (**a**) domains and (**b**) conserved motifs detected in *Jc*DHX proteins according to the DEAD, DEAH, and DExH/D subfamilies. The conserved domains were identified using the SMART software, and the motifs were detected by the MEME program.

In addition, auxiliary domains flanking the N- and C-terminal regions were detected, contributing to the functional diversity of DHX proteins (Figure 4). A distinctive feature of DHX proteins is the presence of up to nine conserved motifs (Q, I, Ia, Ib, II, III, IV, V, and VI), which were detected in the putative *Jc*DHX proteins, showing the conservation of residues and their sequential arrangement (Figure 4; Table S4).

Additionally, the presence and variations in domains and motifs serve as indicators of the DHX subfamily classification. The DEAD subfamily sequences exhibited a canonical structure, with all nine motifs coupled, with a minimal presence of auxiliary domains (Figure 4). Conversely, the DEAH and DExH/D subfamilies displayed notable diversity in motif presence, accompanied by a greater number of auxiliary domains, such as RecQ, HRDC, HA2, OB_NTP_Bind, DSRM, Dicer, PAZ, RIBOc, R3H, POLAc, rRNA_pro-arch, DSHCT, AAA, HTH_40, SecA_DEAD, SecA_SW, HSA, SANT, DUF1998, and Sec63 (Figure 4). This comprehensive analysis provides valuable insights into the structural nuances that underlie functional diversity within the *Jc*DHX protein family.

### 3.6. Physicochemical Characteristics and Subcellular Localization of JcDHX Proteins

The comprehensive characterization of *Jc*DHX proteins was extended to their physicochemical attributes, including protein size (aa), molecular weight (MW), isoelectric point (pI), and potential subcellular localization (Table S5). Regarding protein size, the *Jc*DHX proteins exhibited a substantial range, varying from 317 aa (*Jc*DHX14) to 2247 aa (*Jc*DHX37). The isoelectric point (pI) values spanned from 5.19 (*Jc*DHX54) to 9.97 (*Jc*DHX25), reflecting the diverse charge characteristics of these proteins. In terms of molecular weight, the *Jc*DHX proteins showcased considerable variation, with weights ranging from 34.76 KDa (*Jc*DHX14) to 251.62 KDa (*Jc*DHX37). Such variability in *Jc*DHX proteins across the mentioned parameters underscores the complexity of this protein family.

Prediction analysis of subcellular localization, a crucial factor influencing biological function by regulating access to specific molecular partners, revealed the likelihood of *Jc*DHX proteins being distributed across five distinct cellular compartments. Predominantly, these proteins were predicted to be present in the nucleus (94), cytoplasm (33), chloroplast (nine), mitochondria (seven), and plasma membrane (three) (Table S5). This multifaceted analysis provided valuable insights into the diverse roles of these proteins in different cellular environments.

### 3.7. Prediction of Secondary Structure Elements in JcDHX Proteins

The forecasted secondary structure elements predicted from the *Jc*DHX protein sequences, employing the SOPMA tool (Table S6), revealed a prevailing composition ranging from 17.52% to 60.68% α-helices, 6.84% to 24.37% β-sheets, 0.00% to 11.06% β-turns, and 29.44% to 63.11% random coils. Specifically, the DEAD subfamily members exhibited an average of 40.75% α-helices and 13.47% β-sheets, whereas the DEAH subfamily displayed an average of 40.53% α-helices and 13.35% β-sheets, and the DExH/D subfamily showed an average of 40.07% α-helices and 12.61% β-sheets.

**Figure 5:**
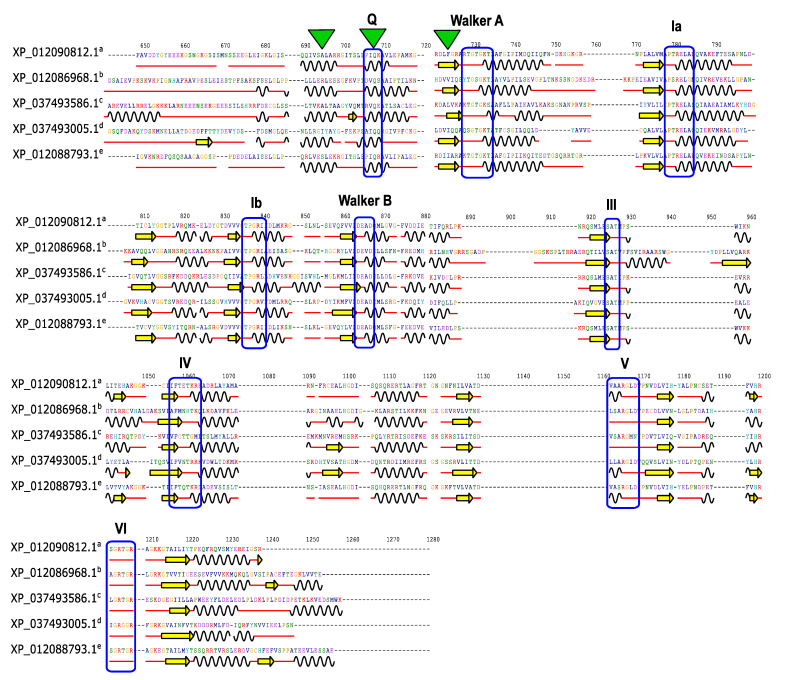
Schematic representation of secondary structure elements in DEAD subfamily proteins encoded by *J. curcas* RNA helicase genes. Curved black lines represent α-helices, horizontal yellow arrows represent β-sheets, and red lines represent the transition regions between structures. Dotted lines indicate the amino acids present in other proteins within the analysis. Blue rectangles highlight conserved motifs in the sequences, whereas green triangles emphasize the β-sheets and α-helices that make up the cap structure upstream of motif I (Walker A), encompassing motif Q. Superscript letters (a, b, c, d, e) represent *Jc*DHX13, 44, 28, 22, and 49, respectively. This figure illustrates the first 5 out of 42 protein sequences from the graphical representation view of the 2D alignment provided by the 2dSS tool after Ali2D analysis (Figure S4) based on the multiple alignment data generated by ClustalX v2.1 (Figure S5).

Distinctive structural features, referred to as “caps,” have been identified in the *J. curcas* DEAD subfamily proteins. These caps, situated atop domain “1” and depicted as green triangles (Figure 5), are composed of a β-sheet and two α-helices located just above the Walker A motif. Additionally, conserved motifs, such as motifs I (Walker A) and II (Walker B), primarily reside in the transition regions between the β-sheets and α-helices, as indicated by the red lines. These motifs are shared among the *Jc*DEAD proteins. Therefore, our *Jc*DEAD candidates exhibit similar structural elements, including a conserved cap structure above the Walker A motif, which reinforces their compatibility with their functional roles. The graphical representation of all predicted SSEs (Figure S4) considers the multiple alignment (ClustalX v2.1) of 42 *Jc*DEAD subfamily proteins, as visualized by the Jalview software (Figure S5).

### 3.8. Homology Modeling of JcDHX Candidates

**Figure 6:**
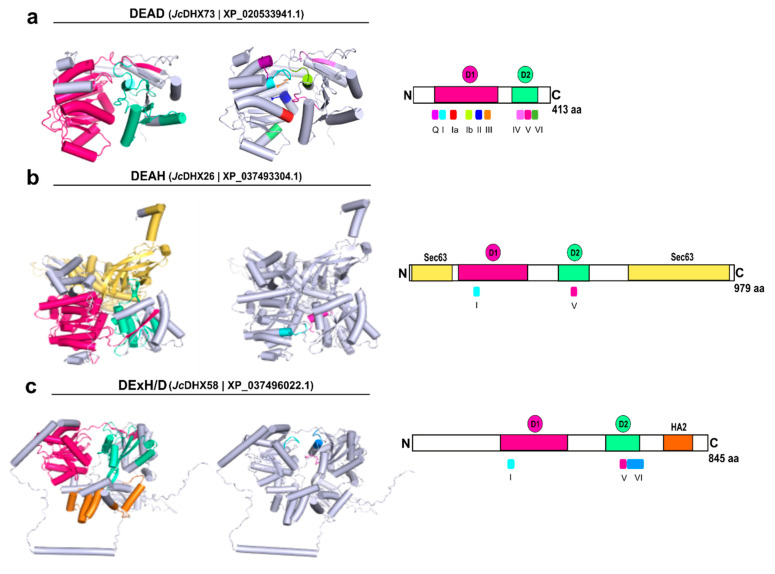
Best 3D models generated for *Jatropha curcas* DEAD-box helicases using the Swiss-model [(**a**) DEAD, (**b**) DEAH, and (**c**) DExH/D subfamilies], highlighting the presence of conserved domains and motifs. The N-terminal DEAD-box core domains (D1) are colored in pink, with the C-terminal HELIc (D2) in cyan green.

A total of 79 *Jc*DHX proteins, representing distinct *JcDHX* genes, underwent 3D modeling using Swiss-Model. Of these 79 candidates, 71 exhibited a Global Model Quality Estimate (GMQE) exceeding 0.60, with 68 achieving coverage exceeding 90%. The resulting structures displayed 80.72% to 98.05% of residues within permissible regions in Ramachandran plots, and the QMEANDisCo global scores ranged from 0.44 to 0.83 (Table S7; Figure S6). The most optimal model from each subfamily, *Jc*DHX73 (DEAD), *Jc*DHX26 (DEAH), and *Jc*DHX58 (DExH/D), is depicted in Figure 6, emphasizing the conserved motifs and domains. Within N-terminal domain 1 (DEAD-box), motifs Q, I, Ia, Ib, II, and III were arranged, while in C-terminal domain 2 (HELIc), motifs IV, V, and VI were positioned (Figure 6). All models exhibited two central globular domains (core), characterized by enveloped β-sheets surrounded by α-helices, which is a hallmark of the SF2 superfamily (Figure 6 and Figure S7).

### 3.9. In Silico Expression of JcDHX Candidates and the qPCR Assay

Considering the *Jc*DHX proteins uncovered from the RJC1_Hi-C reference genome and the RNA-Seq data of the two *J. curcas* accessions following a 3 h exposure to NaCl (150 mM), the BLASTx analysis (e-value cut-off $e^{-10}$) revealed 384 transcripts associated with 94 non-redundant proteins (the best hits) and 76 *JcDHX* genes. All *Jc*DHX candidates exhibited the expected domains identified using the CDD and SMART tools.

The coding potential of each *Jc*DHX transcript was initially detected for 234 transcripts using the SAMBA tool. Another 135 transcripts encoded ORFs translated by TransDecoder, and these ORFs in the BLASTp analysis showed similarities with cured proteins from the UniProtKB/Swiss-Prot database. Similarly, we applied the ORFfinder tool, with a total of 369 *Jc*DHX transcripts encoding 308 potentially functional proteins (Table S8). This result emphasizes the quality of the analyzed transcriptome [34]. These proteins have conserved domains associated with different processes, such as splicing, rRNA processing, translation, DNA repair, chromatin organization, post-transcriptional gene regulation, and mRNA export (Table S8).

**Figure 7:**
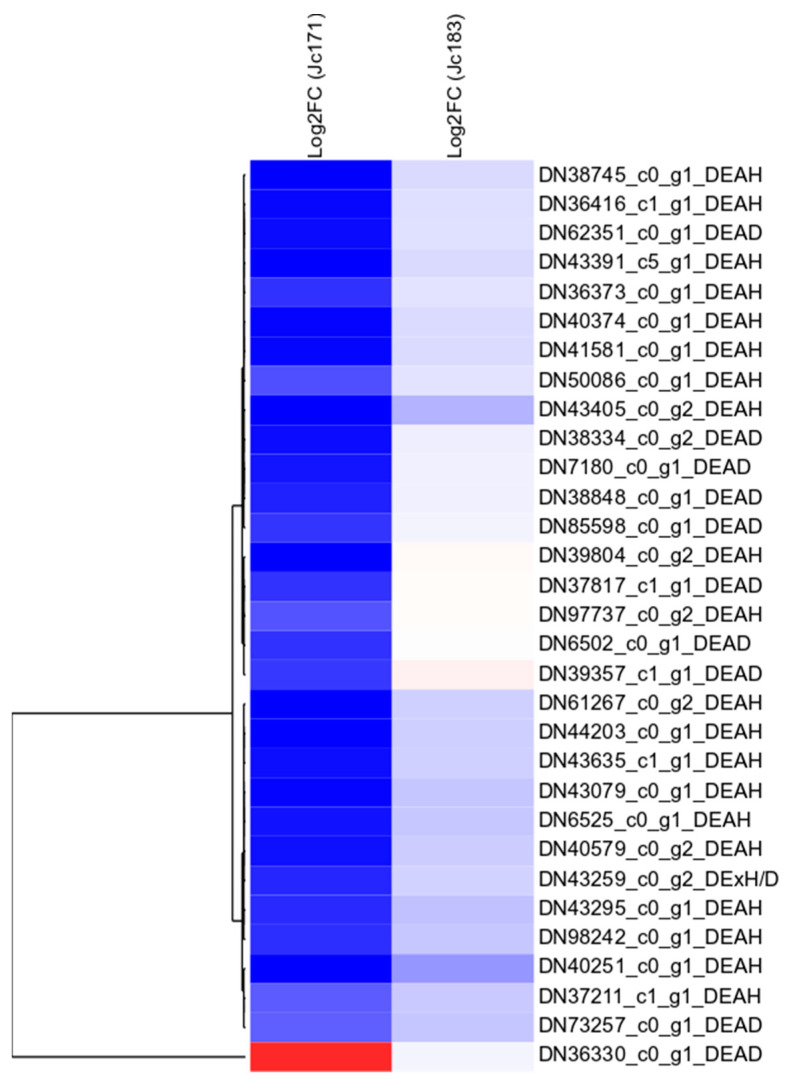
Hierarchical clustering based on Log2FC values of differentially expressed DEAD-box RNA-Seq assembled transcripts (*p*-value < 0.0001; FDR < 0.005) in the roots of accession *Jc*171 subjected to saline stimulation (150 mM of NaCl for 3 h), as well as the respective modulation in *Jc*183 accession.

Concerning the transcriptomic profile of salt-tolerant *Jc*183, this accession did not display any DET (*p*-value < 0.0001; FDR < 0.005) encoding the DHX protein. Concerning the *Jc*171 accession (the less salt-tolerant phenotype), from 120 *JcDHX* transcript isoforms, it comprised 30 repressed DETs [DEAH (20), DEAD (10), and DExH/D (one) subfamilies] and one induced DET (DEAD) (Figure 7). Detailed information about the *JcDHX* transcript isoforms expressed by the *Jc*171 accession is outlined in Tables S8 and S9.

**Figure 8:**
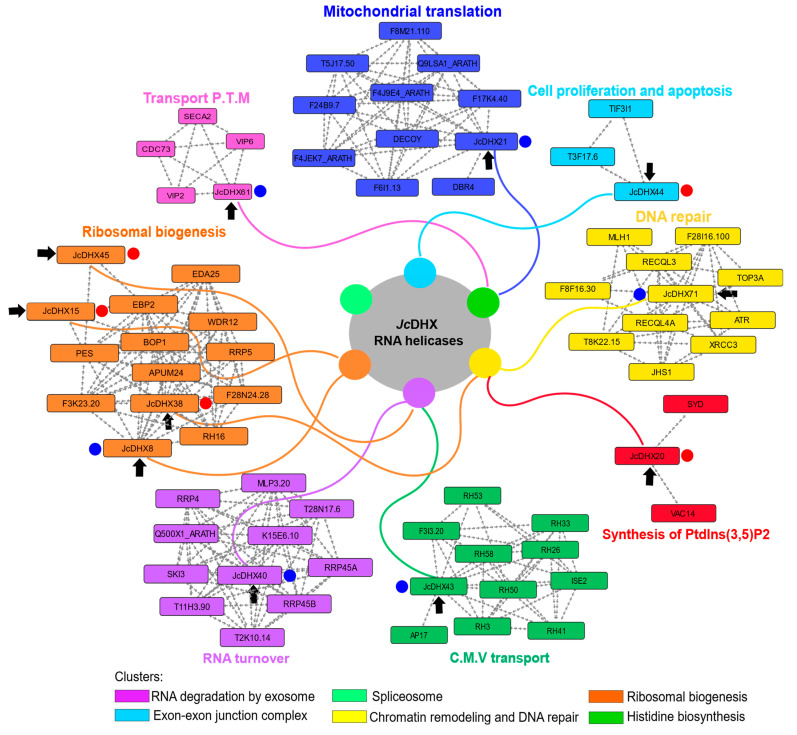
Protein–protein interaction (PPI) network proposed by STRING considering differentially expressed DEAD-box candidates (*J. curcas* RNA-Seq transcripts) also analyzed by qPCR. In the center, there is a representation of the clusters highlighted considering the entire set of expressed *Jc*DHXs. Arrows indicate the *Jc*DHX in the networks. Blue and red circles next to the names of each protein report the qPCR result: blue for downregulation and red for upregulation. *Arabidopsis thaliana* was used as a reference. The confidence score was >0.7 (high confidence). “Transport P.T.M.” represents transport of proteins across the thylakoid membrane, and “C.M.V. transport” represents clathrin-mediated vesicular transport.

Based on the qPCR analysis, out of the 20 proposed primer pairs that successfully amplified the cDNA samples (Table S10), only 13 primer pairs exhibited suitable amplification efficiency (E), slope (s), and correlation coefficient (R) values (Figure S8; Table 1). From the thirteen primer pairs that presented melting curves showing the specificity of the amplicons (Figure S9), nine *JcDHX* candidates confirmed the in silico expression patterns in the qPCR assay, while four other candidates showed a different expression (Table 1; Figure 8).

### 3.10. Protein–Protein Interaction Network

The STRING PPI network (high score > 0.7) based on *A. thaliana* orthologs (Figure 8) of *Jc*DHX proteins encoded by DETs provided a better understanding of the *Jc*171 salt-response profile. The predicted clusters (Figure S10) indicated that *Jc*DHX proteins are part of a complex network in which components of the exosome, spliceosome, and ribosome biogenesis are interconnected. For instance, EIF243 (DEAD-box, *Jc*DHX33) probably acts as a binding platform between the components involved in mRNA degradation and transport, as well as RNA processing (Figure S10). In addition, Ski2 (DEAD-box *Jc*DHX40), a component of the exosome complex, interacts with Ski3, a protein associated with histidine biosynthesis, while clusters are also associated with chromatin remodeling with DNA repair (Figure S10).

The biological processes involving each protein codified by the *Jc*DHX candidates performed in the qPCR assays (Table S11) highlighted the relevance of mitochondrial translation, phosphatidylinositol 3,5-bisphosphate synthesis, thylakoid membrane protein transport, clathrin-mediated vesicular protein transport, cell proliferation, and apoptosis (Figure 8) as those involving *Jc*DHX proteins helping to regulate specific aspects of plants responding to abiotic stress.

## 4. Discussion

### 4.1. Comprehensive Analysis of the DEAD-Box RNA Helicase Family in J. curcas Genome

RNA helicases from the DEAD-box family play pivotal roles in biological processes in prokaryotes and eukaryotes [46,47]. Although extensively studied in the *A. thaliana* model plant or economically relevant crops, such as rice, tomato, cotton, corn, and soybean [7,9,10,48], the *DHX* gene family remains unexplored in *J. curcas* and closely related Euphorbiaceae, including *H. brasiliensis*, *M. esculenta*, and *R. communis*.

Thus, we performed a comprehensive analysis of the DEAD-box family in *J. curcas*, covering classification, gene structure, gene orthologs, and protein characterization, including physicochemical parameters, subcellular localization, conserved domains/motifs, secondary structures, and 3D modeling, together with RNA-Seq analysis of *JcDHX* transcripts of two *J. curcas* accessions, after 3 h of roots exposed to NaCl (150 mM). Understanding the *JcDHX* gene family, based on its gene and protein structures, classification, and evolutionary aspects, will provide insights into its potential roles in *J. curcas* plants in response to salinity.

The 79 putative *JcDHX* genes identified corresponded to 0.35% of the *J. curcas* genes that encode proteins. Similar representativeness was observed in plant species, such as *S. lycopersicum*, *O. sativa*, *Z. mays*, *G. max*, and *Gossypium raimondii* [8,9,10,48]. The structural organization of the 79 *JcDHX* genes revealed a variable number of introns, ranging from 0 to 30 introns. In general, *JcDHX* genes are intron-rich, with members of the DEAH and DExH/D subfamilies exhibiting more complex structures than members of the DEAD subfamily. This pattern aligns with the findings in other plant species, suggesting a conserved characteristic of the family’s genes. Furthermore, considering that genes with multiple introns increase the versatility of the proteome [49], DEAH and DExH/D genes are probably more efficient in producing different isoforms. This corroborates the greater variety of auxiliary domains present in the DEAH and DExH/D subfamilies, as discussed below.

The orthologs of *JcDHX* genes identified in close-related species (*M. esculenta*, *R. communis*, *H. brasiliensis*) and also outside the Euphorbiaceae family (*P. trichocarpa*, *S. lycopersicum*, and *A. thaliana*) showed high gene conservation (87.3%), underscoring their importance in the plant metabolism. The shared orthologs across the studied plant species highlighted the relevance of these RNA helicases in plant biological processes. Notably, *J. curcas* and its taxonomically related species could benefit from *A. thaliana* and *S. lycopersicum* orthologs, given their well-described gene functions, especially in plants exposed to abiotic stress. Further investigations into the functional aspects of *JcDHX* genes in stress responses could pave the way for enhancing the resilience of *J. curcas* and related species.

The physicochemical characteristics and subcellular localization of the *Jc*DHX proteins underscore their functional diversity. These helicases predominantly localize to the nucleus, aligning with their primary role in nucleic acid-related processes such as ribosome biogenesis and the transport of mRNAs from the nucleus to the cytoplasm [50,51]. However, predictions also indicate the presence of *Jc*DHXs in the cytoplasm, where they serve as components of exosomes [52], chloroplasts, and mitochondria, playing roles in gene expression within these organelles [53], and in the plasma membrane, participating in the secretion of proteins through the endoplasmic reticulum [54]. 

All nine characteristic conserved motifs were identified in *Jc*DHX proteins. The motifs are named Q, I, Ia, Ib, II, III, IV, V, and VI. These signatures are directly related to the biochemical activities of helicases. The Q motif acts as a regulator of ATPase activity [55]; motif I (also known as Walker A) is involved in binding to NTP motifs; Ia and Ib are required for RNA binding [56]; motif II (also known as Walker B) is responsible for coordinating ${Mg}^{2+}$ ions, essential for ATP hydrolysis [57]; motif III participates in linking helicase and ATPase activities; motif IV does not have a well-defined consensus and may be functionally connected with motifs V and VI; motif V (in conjunction with Ia, Ib, and IV) acts in binding to RNA and regulating ATP hydrolysis; and motif VI, which interacts with motifs II and III, has been described as important for both ATP hydrolysis and RNA binding [6,55,56,57].

The versatility of *Jc*DHX proteins, as reflected in the variety of auxiliary domains that regulate core helicase activity (DEXDc and HELIc), contributes to their multifaceted functions, such as those associated with gene silencing (DSRM, Dicer, RIBOc), recombination and repair (RecQ-Zn-bind), chromatin remodeling (SANT, SNF2_N), translocation through the membrane (SecA_DEAD), and the endoplasmic reticulum (Sec63). In addition, different domains assist interactions with nucleic acids, such as HDRC, R3H, and HAS. These domains provide functional diversity and specificity for the catalytic reactions of *Jc*DHX proteins [58,59]. Understanding the characteristics of DHX proteins within the same subfamily is crucial as they may perform similar functions in different species.

The phenetic analysis based on motif II variations (D-E-A-D) supports the classification of *JcDHX* genes into three known subfamilies [59]: DEAD (42 members), DEAH (30), and DExH/D (seven). In plants, the number and composition of subfamilies vary significantly across species, reflecting the high diversity of the *DHX* genes [9,10,11,12]. Conserved motifs crucial for protein interactions and functional similarities were identified in *Jc*DHX candidates, with variations observed in motif VI between the DEAD and DEAH subfamilies. These variations require further study. The DEAH and DExH/D subfamilies showed more considerable motif variations and a higher occurrence of auxiliary domains compared to the DEAD subfamily, reinforcing their genetic diversity and functionalities [8,9,10,11,12].

The differences in the conserved motifs contribute to structural variations, promoting the flexibility required for diverse activities [58,59]. These structural changes favor the emergence of specialized proteins with distinct cellular activities, suggesting that the DEAH and DExH/D subfamilies may participate in more specific pathways than the more basic functions of the DEAD subfamily members. The versatility of *J. curcas* DEAD-box RNA helicases likely stems from their diversity within the DEAH and DExH/D subfamilies.

Typical secondary structures of DEAD-box helicases include a β-sheet and two α-helices, forming a “cap” upstream of motif I. These structures are associated with the presence of the Q motif, which acts as a regulator of ATPase activity [55]. The 3D models confirmed the presence of two core domains comprising β-sheets surrounded by α-helices, with motifs positioned in the cleft between the two domains. This characteristic resembles the RecA-like ATPase folding pattern and is consistent with the known structures in the family [57]. These structural characteristics confirm that *J. curcas* DEAD-box helicases are well suited for nucleic acid binding, NTP hydrolysis, and strand unwinding activities [55,57,59,60].

### 4.2. Regulatory Landscape of JcDHX Genes: Insights into Cis-Regulatory Elements

*Cis*-regulatory elements (CREs) within gene promoters are pivotal for gene expression regulation by serving as recognition sites for TFs. In the analysis of the promoter regions of *JcDHX* genes, a diverse array of TFs spanning different families (Dof-type, BBR-BPC, HD-ZIP, AP2-ERF, WRKY, bHLH, and Myb-related) were identified, with particular emphasis on Dof-type TFs. These TFs, prominently featured in plant responses to abiotic stress [61,62,63], have demonstrated key roles in orchestrating stress-responsive gene expression. Dof-type TFs, which are characterized by a DNA-binding zinc finger, have emerged as critical regulators of plant environmental stress responses. Examples include *MtDof32* in *A. thaliana*, which confers enhanced tolerance to osmotic and salt stress [64], and *GhDof1* in cotton, contributing to salinity and cold tolerance [65]. The overexpression of the cotton *GhDof1.7* gene in *A. thaliana* transgenic plants exhibited increased salinity tolerance, accompanied by reduced ROS accumulation and elevated activities of superoxide dismutase (SOD) and catalase (CAT) enzymes [66]. These findings underscore the potential of Dof-type TFs to modulate plant responses to abiotic stress. Thus, the association of Dof-type TFs with CREs on the promoters of *JcDHX* genes also suggests the involvement of *JcDHX* genes in the broader context of stress-responsive genes.

### 4.3. Differential Regulation of JcDHX Genes in J. curcas under Salt Stress

In order to decipher the expression profile of *JcDHX* transcripts in *J. curcas* roots after 3 h of NaCl (150 mM) exposure, RNA-Seq analysis revealed a distinctive response of the *Jc*171 accession, the less salt-tolerant phenotype. In the biological assay, after 3 h of NaCl exposure, only *Jc*171 plants showed visible leaf damage, whereas *Jc*183 showed a salt-tolerant phenotype [35]. Concerning the respective RNA-Seq transcriptomes, *Jc*183 almost did not modulate its transcriptome significantly after salt treatment [35]. On the other hand, the present study identified, from the *Jc*171 salt-response profile, 116 *JcDHX* transcripts, with 68 (58.6%) of them declared DETs (related to 22 *JcDHX* genes) and 67 DETs showing a remarkable downregulated response (DR).

In general, analysis of the RNA-Seq transcript isoforms identified six auxiliary domains that were not predicted in our reference sequences (Table S8): DBINO, RING, PHD, HIRAN, CHROMO, and PWI. The DBINO domain (DN62351_c0_g1_i1) is related to DNA binding activities [67], the RING domain (DN36373_c0_g1_i1) is related post-translational modification in proteins [68], the PHD domain (DN36416_c1_g1_i1) to the recognition of methylated histones [69], the HIRAN domain (DN43635_c1_g1_i1) to recognition of damaged DNA [70], the CHROMO domain (DN6525_c0_g1_i1) interacts with methylated histones [71], and the PWI domain (DN43405_c0_g1_i1) with the processing of pre-RNAs [72]. The combination of these domains was also observed in DN43635_c1_g1_i2, which presented the HIRAN and RING domains, and whose expression was validated by qPCR (Table 1). Protein isoforms presenting these unpredicted domains can add functional diversity to the helicases expressed by the studied accessions after salinity exposure, highlighting, for example, the interaction with histone methylation that could be important in chromatin remodeling and gene expression.

Regarding the subcellular location of the proteins encoded by the isoforms, 12 isoforms pointed to the extracellular region, which was not predicted by the reference proteins. Interestingly, of the 12 isoforms, 10 were expressed only by *Jc*183 (Table S8). Helicases in the extracellular region have already been reported in *Arabidopsis* defense against the pathogenic fungus *Botrytis cinerea*, which is correlated with the transport of small RNAs (sRNAs) in extracellular vesicles [73]. Such correlations concerning abiotic stress have not yet been reported.

Comparing the different isoforms, especially those related to DETs, differences in subcellular localization were observed less than changes in relation to domains. Two isoforms of the assembled transcript, DN41581_c0_g1, for example, predicted to act in the nucleus, showed differences in terms of the DEXDc/HELICc/Dicer/PAZ/RIBOc domains (Table S8). One of the isoforms (DN41581_c0_g1_i2) had three more domains (PAZ/RIBOc/RIBOc) than the other (DN41581_c0_g1_i1). The presence of more domains reinforce function or provide functional variability. The PAZ domains (Piwi/Argonaute/Zwille), RIBOc, and Dicer were related to post-transcriptional gene silencing [74], and one of the isoforms, DN41581_c0_g1_i2, was validated in the qPCR assay. In turn, the three isoforms of the assembled transcript DN43635 (Table S8) showed differences both in subcellular localization (nucleus and cytoplasm) and in the detected domains (DEXDc/HELICc/HIRAN/RING), with one of the isoforms (DN43635_c1_g1_i2) showing an extra HELICc domain. The gene expression of this isoform was validated by qPCR. Furthermore, the DN62351_c0_g1_i1 isoform, induced in the qPCR assay, presented a DBINO domain that was not detected in the reference protein (Table S8). These variations were also observed in assembled transcripts that did not show differential expression after saline exposure. For example, the products encoded by the six isoforms of the assembled transcript DN42995_c0_g2, which would be addressed to the nucleus, chloroplast, or cytoplasm, showed variations in the distribution of the HELICc and HA2 domains, which are related to helicase activity and nucleic acid binding, respectively. Overall, this sample provides insights into the diversity of DHX transcripts and protein isoforms that can be expressed by the two studied accessions, as well as their involvement in several important metabolic processes in plant metabolism.

DHX genes respond to abiotic stress in plants. qPCR results from 42 *DHX* genes in tomato leaves under salinity, drought, cold, and heat stress conditions revealed 14 genes induced across all scenarios, with five genes (*SlDEAD24*, *SlDEAD32*, *SlDEAD34*, *SlDEAD35*, and *SlDEAD42*) significantly induced during salinity exposure [75]. In *Arabidopsis*, *AtRH9* and *AtRH25* were induced under cold stress and repressed under drought and salinity conditions [76]. Further corroborating these findings, qPCR analyses in tomato plants demonstrated the induction of *SlDEAD30* and *SlDEAD31* genes exposed to salinity [14], as well as *SlDEAD25* and *SlDEAH15* genes exposed to 200 mM of NaCl [10]. Additionally, various genes (*SlDEAD23*, *SlDExD/H9*, and *SlDEAD35*) were induced in tomato leaves under drought, salinity, heat, and cold stress [10]. More specific studies are necessary to explore the observed gene expression profiles efficiently.

The scientific literature supports that some *DHX* genes stand as promising candidates for biotechnological exploration, aiming to develop stress-tolerant genotypes. Some successful cases comprise transgenic tomato plants overexpressing the *SlDEAD31* gene, showcasing heightened salinity and moderate drought tolerance [14]; the *AtRH17* gene in *A. thaliana* plants conferring salinity tolerance [77]; the wheat *TaDEAD-57-3B* gene, improving proline and chlorophyll levels and enhancing drought and salinity tolerance in *A. thaliana* [12]; the pea DEAD-box *Psp68*, improving rice salinity tolerance, marked by reducing the MDA levels [78]; and the overexpression of the DEAD-box *BrDHC1* in *Brassica rapa*, increasing the drought tolerance by enhancing water retention, chlorophyll content, and activities of antioxidant enzymes [79].

However, some DEAD-box proteins are negative regulators during abiotic stresses. In grape plants (*Vitis vinifera* L.), leaf RNA-Seq data showed more than 70% repressed DEAD-box genes (28 of 40) in response to drought (1, 2, 4, 8, and 24 h of irrigation suppression) [11]. *Arabidopsis thaliana* plants overexpressing *VviDEADRH25a* presented higher drought sensitivity [11]. In turn, the *Arabidopsis* DEAD-box *STRS1* and *STRS2* genes were repressed under various abiotic stresses (heat, drought, salinity—200 mM NaCl: 1, 3, 6, 12, 24, 48 h) and they acted by attenuating the expression of the transcriptional activators DREB1A/CBF3, DREB2A, and RD29A, which function in both ABA-dependent and ABA-independent pathways [80].

Our RNA-Seq study only pointed out the nuances of *JcDHX* genes expressed in the salt response of *Jc*171, offering insights into its acclimatization strategies to salt stress, since *Jc*183 did not modulate its *JcDHX* genes significantly after the salt treatment, indicating a distinct adaptive response. The tolerant accession *Jc*183 showed little modulation of its transcriptome, with only 57 DETs in previous works [35,81], indicating that it is not only the gene category of DHX helicases that has no modulation, being in agreement with the general response of the accession. Furthermore, *Jc*183 has already been described to support up to 750 mM of NaCl, showing rapid recovery from salinity after the alleviation of the saline conditions in the soil, and scanning electron microscopy revealed that the stomata of *Jc*183 are smaller and have a higher stomatal index compared to those of the *Jc*171 genotype [82]. On the other hand, *Jc*171 has also been reported as salt-tolerant after exposure to some levels of salinity [34], and our transcriptome analysis indicated most of the identified DHX candidates as repressed. In turn, the observed repression may not have contributed to the salt tolerance phenotype of *Jc*171, or it at least did not efficiently explore the *DHX* genes. Thus, this differential salt response showed by the two accessions underscores a genotype-dependent pattern of the *DHX* gene families after salinity exposure.

### 4.4. Unraveling Functional Networks with JcDHX Proteins in Salinity Response

Observing the predicted PPI network, our *JcDHX* candidates and pivotal functional partners revealed distinct clusters pointing to crucial cellular processes, such as DNA repair and chromatin remodeling, splicing, RNA degradation, ribosome biogenesis, and histidine biosynthesis. Inside the PPI network, a *Jc*DHX37 ortholog (*At*DEAH11) assumed a central position. Together with other proteins, they underscored the significance of RNA metabolism, with a particular emphasis on rRNA processing, alternative splicing, and mRNA degradation. Alternative splicing (AS), a regulatory process impacting diverse physiological aspects, also spotlighted abiotic stress responses, including salinity. An RNA-Seq of *A. thaliana* plants from NaCl-treated seeds disclosed that 49% of genes with introns underwent splicing alterations, with 10% undergoing differential alternative splicing (SAD) [83]. In addition, after salinity exposure, plants of *Gossypium davidsonii* exhibited a significant increase (32%) in genes undergoing splicing alterations [84], while a crucial role in the stress response was played particularly by the *SHI2* gene, with its DEAD-box activity in splicing cold response genes [85]. Another distinct cluster highlighted the biosynthesis of histidine, an amino acid crucial for plant growth, development, and responses to environmental factors, including salinity. A treatment with histidine in corn plants, when exposed to salinity, exhibited enhanced tolerance and increased activities of antioxidant enzymes after salt stress [86], while the induction of histidine biosynthesis enzymes in tomato leaves under heat and flooding stresses further underscored its importance [87].

In addition, regarding the particular contribution of each *JcDHX* DET in the *Jc*171 salt-response profile, according to the predicted PPI networks, some processes highlighted spanned DNA repair, RNA turnover, ribosomal biogenesis, mitochondrial translation, protein transport across the thylakoid membrane, clathrin-mediated protein vesicular transport, phosphatidyl 3,5-bisphosphate (PtdIns(3,5)) synthesis, and cell proliferation/apoptosis.

Considering the implications of ionic and osmotic stresses on ROS accumulation and the ensuing damage to DNA, unrepaired DNA damage can lead to genomic instability, disrupting cellular functions and potentially resulting in cell death [88]. In this context, the PPI network revealed interactions of *Jc*DHX71 with DNA repair and cell cycle checkpoint partners, emphasizing the rule of this helicase in maintaining genomic integrity during salinity stress. Additionally, DNA repair pathways, integral to cell cycle checkpoints, play a crucial role in plant development and stress adaptation [89,90]. Acting as damage sensors, checkpoint proteins intervene in cell cycle arrest, allowing DNA lesion repair to ensure normal cellular functioning [91]. However, the downregulation of candidate *Jc*DHX71, in both in silico and qPCR analyses, underlines its unique regulatory pattern of *Jc*171 after salinity exposure.

In RNA turnover, the interplay between the RNA exosome machinery, responsible for 3’-5’ degradation and the processing of various RNA classes, and DEAD-box RNA helicases influences stress granules (SGs) and processing bodies (P-bodies) dynamics [92,93]. These cytosolic ribonucleoprotein complexes are stimulated by various stresses, including oxidative stress, and modulate mRNA translation, storage, and degradation, conserving energy for translational machinery during stress conditions [94]. The predicted PPI network implicated *Jc*DHX40 in the RNA exosome pathway, and both in silico and qPCR analyses showed its downregulation in *Jc*171 after salt stress.

Several DEAD-box RNA helicases contribute to ribosomal biogenesis, a fundamental process involving rRNA maturation and assembly with ribosomal proteins [95,96]. However, salinity-induced nucleolar stress affects pre-rRNA accumulation, leading to nucleolar cavity formation and the activation of apoptotic pathways [97]. The predicted PPI network highlighted interactions of *Jc*DHX38, *Jc*DHX8, *Jc*DHX15, and *Jc*DHX45 with partners involved in rRNA processing. Besides the downregulation detection in silico data of the four candidates, the qPCR results only confirmed the downregulation of one of them, reminiscent of the essential role of DEAD-box helicases in maintaining ribosomal homeostasis.

The eIF3 complex, a key player in translation initiation, controls cyclin-dependent kinases (CDKs) linked to cell proliferation, cell cycle progression, and programmed cell death (PCD) [98,99]. Crucial for maintaining cellular homeostasis, PCD is part of the plant’s defense against stress [100]. Salinity-induced PCD and autophagy in halophyte cells underscore its role in stress responses [101]. The PPI network predicted interactions of *Jc*DHX44 with proteins of the eIF3 complex, and this candidate was upregulated in both in silico and qPCR analyses.

Mitochondria, pivotal in energy production and cell signaling, undergo stress-induced alterations affecting mitochondrial translation and protein transport [102,103]. Disruption in mitochondrial processes triggers the mitochondrial unfolded protein response pathway, essential for restoring mitochondrial homeostasis [104,105]. The PPI network implicated *Jc*DHX21 in mitochondrial translation interactions. Besides the downregulation of *JcDHX21* in both in silico and qPCR analyses, the constitutive expression of *Os*SUV3, a mitochondria-localized DEAD-box helicase, enhanced salinity tolerance in rice plants [106], emphasizing the potential of *Jc*DHX21 in a potential salinity response.

Under osmotic stress conditions, alterations in plasma membrane balance are crucial. Clathrin-mediated transport vesicles play a pivotal role in protein trafficking between membrane systems. This system is directly linked to the abundance and localization of aquaporins (type PIP) in the membrane, crucial for water absorption by roots during salinity conditions [107]. Additionally, the clathrin system is implicated in stomatal function in *A. thaliana* [108] and ROS accumulation under salinity stress [109]. The PPI network predicted interactions of *Jc*DHX43 with partners involved in clathrin-mediated vesicular protein transport; this candidate also exhibited downregulation in both in silico and qPCR data.

During osmotic stress in plants, inositol phospholipids play diverse signaling roles in cells, with rapid accumulation of phosphatidyl 3,5-bisphosphate (PtdIns [3,5]) [110], which is essential for normal vacuole function, since its depletion results in aberrant vacuoles [111]. The PtdIns(3,5) metabolic pathway has been associated with critical aspects of stress response, including stomatal conductance [112], vacuolar convolution [113], and activation of V-ATPases [114]. The PPI network predicted interactions of *Jc*DHX20 with proteins involved in PtdIns(3,5) synthesis. *Jc*DHX20 was downregulated in silico and upregulated in the qPCR assay.

In summary, predicted interactions of *Jc*DHX candidates showcase central roles in the plant’s adaptive strategies against salinity exposure. However, our *Jc*DHX candidates were not sufficiently induced to confer a positive salt response of the *Jc*171 accession, diminishing the impact of these processes highlighted by the PPI networks. Therefore, the lower-tolerance phenotype must also be associated with the impairment of these processes due to the repression of these *Jc*DHX candidates. Additionally, the upregulation of certain candidates initially identified as repressed but confirmed as upregulated by qPCR assays may underscore the relevance of DHX proteins in *Jc*171’s salt response, thereby deepening our understanding of these proteins.

## 5. Conclusions

This study comprehensively obtained and analyzed the entire family of DEAD-box RNA helicases from *Jatropha curcas*. A total of 79 *JcDHX* genes were identified, a quantity proportional to that observed in other species. Orthology analyses involving *M. esculenta*, *R. communis*, *H. brasiliensis*, *P. trichocarpa*, *A. thaliana*, and *S. lycopersicum* highlighted the conserved nature of the family and its significance across these species. The proteins were classified based on known subfamilies (DEAD, DEAH, and DExH/D), and our observations revealed that the pattern of domains, motifs, and exon–intron structures reflected this classification. Helicases from the DEAD subfamily exhibited the classic structure composed of the nine characteristic conserved motifs, featuring small C- and N-terminal extensions and very few auxiliary domains, in addition to simpler gene structures. In turn, the DEAH and DExH/D helicases are more complex in terms of both domains and gene structure, although they have shown notable variations in the presence of conserved motifs. The three-dimensional models of *Jc*DHX generated are consistent with the functions performed by these enzymes. Taken together, the analyses indicate that *Jatropha curcas* DEAD-box RNA helicases constitute a highly structurally diverse family contributing to the execution of a variety of functions. The presence of candidates for cis-regulatory elements (CREs) in the promoters of *JcDHX*, associated with important transcription factors such as Dof-type, BBR-BPC, HD-ZIP, and bHLH, demonstrates that these genes can participate in crucial pathways during environmental stresses. The repertoire of transcripts in *Jc*DHX was modulated in response to salinity stimulation, particularly in the *Jc*171 accession. Protein–protein interaction networks revealed significant functional partners of *Jc*DHX expressed in response to salinity, demonstrating their crucial role in plants under stress. Considering all of the results and acknowledging the significance of DEAD-box RNA helicases in RNA metabolism, the observed transcriptional response may be associated with the reduced capacity of *Jc*171 to cope with the applied salinity stimulus. This study contributes valuable insights into the structural and functional aspects of *Jc*DHX helicases, providing a foundation for future functional characterizations of these genes concerning their involvement in responses to abiotic stresses.

## Figures and Tables

**Table 1:** In silico results and relative gene expression (qPCR) of RNA-Seq *Jc*DHX transcripts of *J. curcas* accession *Jc*171 after exposure to 150 mM of NaCl for three hours. Data analysis performed using REST© software (v.2.0.13). DR: downregulation; UR: upregulation.

							Result	
RNA-Seq Transcript	Gene	Efficiency (%)	Relative Expression	Std. Error	95% C.I.	P(H1)	In Silico	qPCR
DN43295_c0_g1_i2	*JcDHX43*	102.34	−0.54	0.237–1.111	0.105–1.999	0.047	DR	DR
DN43259_c0_g2_i2	*JcDHX43*	95.76	−0.22	0.083–0.694	0.033–2.514	0.000	DR	DR
DN39804_c0_g2_i2	*JcDHX8*	108.19	−0.30	0.051–1.709	0.024–2.832	0.043	DR	DR
DN43635_c1_g1_i2	*JcDHX71*	99.72	−0.13	0.012–1.999	0.001–3.936	0.032	DR	DR
DN36330_c0_g1_i1	*JcDHX44*	102.98	3.50	1.451–14.283	1.043–30.645	0.000	UR	UR
DN97737_c0_g2_i1	*JcDHX40*	101.48	−0.49	0.346–0.686	0.236–0.840	0.000	DR	DR
DN43391_c5_g1_i3	*JcDHX40*	97.43	−0.51	0.380–0.704	0.296–0.867	0.000	DR	DR
DN39804_c0_g2_i1	*JcDHX21*	90.38	−0.60	0.345–1.059	0.214–1.426	0.014	DR	DR
DN41581_c0_g1_i2	*JcDHX61*	108.16	−0.68	0.398–1.046	0.282–2.043	0.031	DR	DR
DN40374_c0_g1_i2	*JcDHX20*	109.42	1.97	1.135–3.584	0.677–5.953	0.004	DR	UR
DN62351_c0_g1_i1	*JcDHX38*	95.05	1.78	1.273–2.527	0.785–3.596	0.000	DR	UR
DN7180_c0_g1_i1	*JcDHX45*	91.69	5.56	0.966–28.716	0.185–79.341	0.012	DR	UR
DN85598_c0_g1_i1	*JcDHX15*	98.48	1.42	0.942–2.299	0.573–3.147	0.042	DR	UR

## Data Availability

Data are contained within the article and Supplementary Materials.

## Supplementary Materials

The following supporting information can be downloaded at https://www.mdpi.com/article/10.3390/plants13060905/s1: Figure S1: MEME logo of the motifs detected in the promoter regions (1000 bp upstream the TSS—transcription start site) of the *JcDHX* genes. For each motif, the e-value and JASPAR ID are reported; Figure S2: Distribution of candidate cis-regulatory elements (CREs) (*p*-value < 0.01) in the promoter regions (1.0 kb) of *JcDHX* genes. Colored boxes provide information about CREs, JASPAR IDs, and associated transcription factors. * Combined MEME *p*-value; ‡ statistical significance below the considered cut-off (e-value < 0.05; *p*-value < 0.01); Figure S3: Graphical representation of (a) the number of identified *JcDHX* orthologs and (b) a Venn diagram illustrating the sharing of these genes among the analyzed species; Figure S4: Graphical representation of predicted secondary structure elements in the proteins of 42 *J. curcas* RNA helicase genes from the DEAD subfamily. A 2D alignment view was generated using the 2DSS tool and Ali2D analysis, considering multiple sequence alignment data (ClustalX v2.1); Figure S5: Multiple alignment of 42 proteins from *J. curcas* RNA helicase genes of the DEAD subfamily. This data alignment (ClustalX software v2.1) was employed in the Ali2D analysis to predict secondary structure elements; Figure S6: Ramachandran plots generated by Swiss-model from models built for *Jc*DHX; Figure S7: Three-dimensional homology models proposed for *Jc*DHX from the Swiss-model. The structures were visualized and edited using PyMOL software; Figure S8: Standard curves demonstrating the efficiency of the *Jc*DHX primer pairs. The data were generated from serial dilutions made with cDNAs from the cultivars (undiluted, 1:10, 1:100, 1:1000, and 1:10,000); Figure S9: Dissociation curves generated between 65 and 95 °C, demonstrating the specificity of the products amplified by the *Jc*DHX primers; Figure S10: Protein–protein interaction network constructed by STRING (high confidence > 0.70), considering all differentially expressed *Jc*DHX transcripts (*p*-value < 0.0001; FDR < 0.005) in RNA-Seq libraries from *Jatropha curcas* roots (*Jc*171) subjected to saline stimulation (150 mM of NaCl for 3 h). Six clusters were highlighted from the set, with proteins (nodes) colored with the same colors predicted to be components of the same cluster. Dashed lines indicate interactions between distinct clusters; Table S1: Characteristics of *Jatropha curcas* genes (*Jc*DHX) encoding DEAD-box RNA helicases: name (ID), subfamilies, *locus* ID, genomic location, gene length, number of introns, CDS length, primary protein at each *locus*, and transcript variants; Table S2: Motifs detected as candidate cis-regulatory elements (CREs) by the MEME and TOMTOM programs coupled to the JASPAR database; Table S3: Orthologs to genes encoding DEAD-box helicases from the *Jatropha curcas* RJc1_Hi-C genome (GCF_014843425.1); Table S4: Conserved motifs detected by the MEME program in *Jatropha curcas* DEAD-box helicases (*Jc*DHX). Motif VI had two variations detected (MEME-1 and MEME-7); Table S5: Physicochemical characteristics of proteins encoded by *JcDHX* genes, their sequences and predicted subcellular localization. ‘aa’: amino acids; ‘pI’: isoelectric point; ‘MW’: molecular weight ‘KDa’: kilodalton; Table S6: Secondary structures detected in *Jc*DHX proteins performing the SOPMA tool; Table S7: Quality parameters obtained for the 3D models generated for the *Jatropha curcas* DEAD-box RNA helicases (*Jc*DHX) using the Swiss-model; Table S8: DEAD-box transcripts (*Jc*DHX) identified in RNA-Seq libraries (BLASTx e-value cut-off $e^{-10}$) for accessions *Jc*171 and *Jc*183 after exposure to 150 mM of NaCl for 3 h. For each transcript, it is possible to access the transcript isoform; *J. curcas* accession; Trinity unigene (‡ corresponds to differentially expressed unigene); regulation (DEG); classification regarding the coding nature (‘*’ corresponds to a transcript verified manually using the ORF finder) transcript sequence; annotation; domain CDD; encoded protein; predicted protein (aa); domain (SMART); subcellular location; BLASTx e-value and information about of the *J. curcas* RJC1_Hi-C genome-associated proteins; Table S9: Information on each assembled transcript in different samples. For each assembled transcript, it is possible to access the subfamily, regulation, Log2FC, *p*-value, FDR, and normalized FPKM data in the *Jc*171 transcripts that are also expressed in *Jc*183. “n.d” corresponds to Jc183 transcripts that were not expressed in *Jc*171; Table S10: Primers designed for *Jc*DHX transcripts identified in RNA-Seq libraries for accession *Jc*171 after exposure to 150 mM of NaCl for 3 h. ‘Tm’: Melting temperature; Table S11: Data on protein–protein interaction networks constructed by STRING (High score > 0.7) from *Jc*DHX associated with transcripts differentially expressed in qPCR.

## Abbreviations

AAA (ATPases associated with a variety of cellular activities); AP2-ERF (Apetala 2-Ethylene Response Factor); AS (alternative splicing); ATP (Adenosine TriPhosphate); BBR-BPC (Barley B Recombinant/Basic PentaCysteine); bHLH (basic/helix–loop–helix); BROMO (Bromodomain); C.M.V (clathrin-mediated vesicular transport); SAD (differential alternative splicing); CAT (catalase); CDD (Conserved Domain Database); CDS (coding sequence); CREs (cis-regulatory elements); DEAD (Asp-Glu-Ala-Asp); DETs (differentially expressed transcripts); Dof (DNA-binding One Zinc Finger); DR (downregulated); DSHCT (DOB1/SK12/helY-like C-terminal domain); DSRM (Double-Stranded RNA-binding Motif); DUF (Domain of unknown function); FC (fold change); FDR (false discovery rate); GMQE (Global Model Quality Estimate); GSDS (Gene Structure Display Server); GUCT (Gu C-terminal domain); HA2 (Helicase associated 2); HAS (helicase/SANT-associated); HD-ZIP (Homeodomain–leucine zipper); HMM (Hidden Markov Model); HRDC (helicase and RNaseD C-terminal); HTH_40 (helix–turn–helix); iTOL (Interactive Tree of Life); *Jc*DHX (*Jatropha curcas* DEAD-box); MDA (Malondialdehyde); MEME (Multiple Em for Motif Elicitation); MYB (myeloblastosis); NCBI (National Center for Biotechnology Information); NTP (nucleoside triphosphate); P.T.M. (transport of proteins across the thylakoid membrane); PAZ (Piwi Argonaut and Zwille); PFAM (protein family); POLAc (DNA polymerase A); PIP (plasma membrane intrinsic proteins); PPI (protein–protein interaction); PtdIns(3,5) (phosphatidyl 3,5-bisphosphate); RIBOc (ribonuclease); ROS (reactive oxygen species); RQC (RecQ C-terminal); SANT (SWI3, ADA2, N-CoR and TFIIIB); SF2 (Superfamily 2); SGs (stress granules); SMART (Simple Modular Architecture Research Tool); SNF2 (Sucrose non-fermenting); SOD (superoxide dismutase); TAIR (The Arabidopsis Information Resource); TF (transcription factor); UR (upregulated); WD40 (WD [Trp-Asp] or beta-transducin repeats).
