# Supplementary material for: DEAD-Box RNA Helicase Family in Physic Nut (Jatropha curcas L.): Structural Characterization and Response to Salinity
Source: Plants (Basel). 2024 Mar 21;13(6):905. doi: 10.3390/plants13060905 (PMC10974417; doi:10.3390/plants13060905)
Supplement: Supplementary file 1 [file plants-13-00905-s001.zip › Supp_Mat/Figures/Figure S1.pdf]

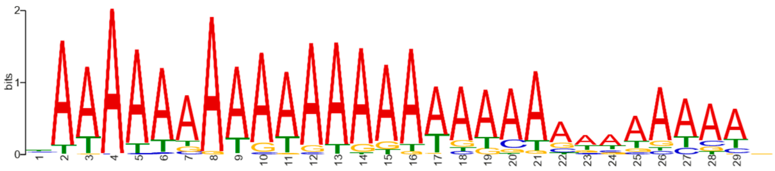

MA1267.1|Dof-type   e-value: 2.8e-164

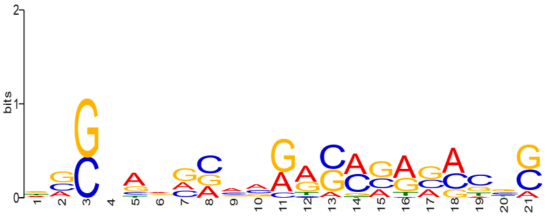

MA1402.1|BBR-BPC   e-value: 9.8e-059

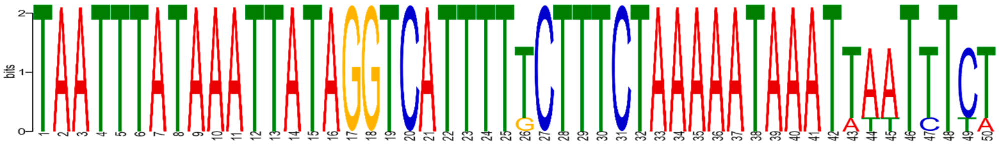

MA1214.1|HD-ZIP   e-value: 4.4e-035

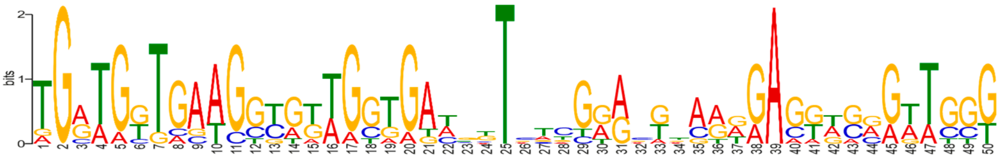

MA1260.1|AP2-ERF   e-value: 2.0e-009

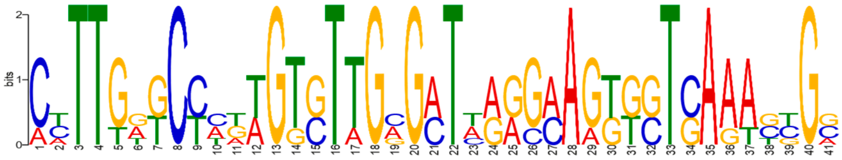

MA1090.1|WRKY   e-value: 3.2e-006

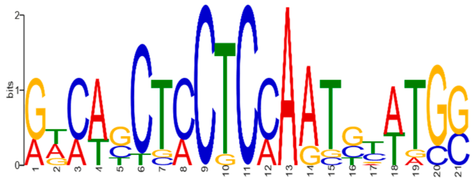

MA1284.1|bHLH   e-value: 1.6e-004

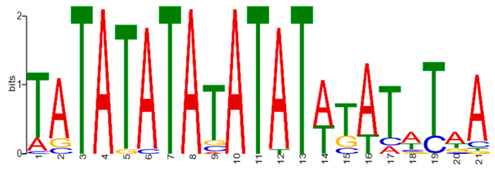

MA1191.1|Myb-related   e-value: 5.2e-007

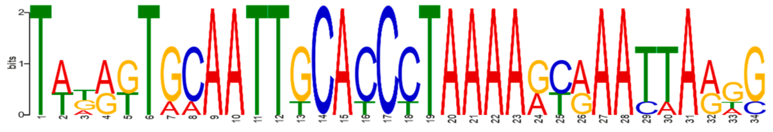

MA1355.1|Myb-related   e-value: 4.6e-004
