# Supplementary material for: DEAD-Box RNA Helicase Family in Physic Nut (Jatropha curcas L.): Structural Characterization and Response to Salinity
Source: Plants (Basel). 2024 Mar 21;13(6):905. doi: 10.3390/plants13060905 (PMC10974417; doi:10.3390/plants13060905)
Supplement: Supplementary file 1 [file plants-13-00905-s001.zip › Supp_Mat/Figures/Figure S10.pdf]

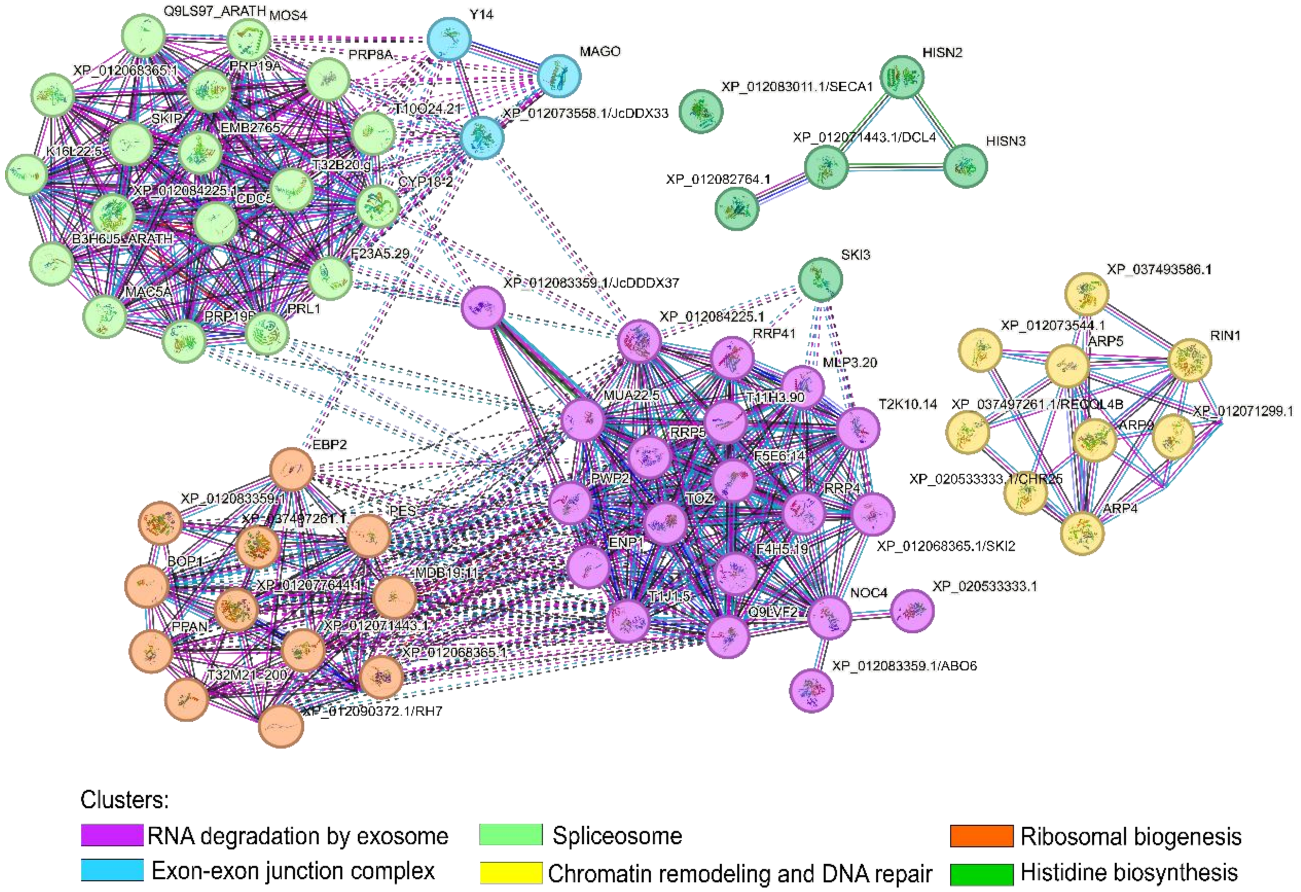
Q9LS97_ARATH
MOS4
Y14
MAGO
PRP8A
XP_012068365.1
PRP19A
T10O24.21
XP_012073558.1/JcDDX33
SKIP
EMB2765
K16L22.5
T32B20.g
XP_012084225.1
CDC5
CYP18-2
B3H6J5_ARATH
F23A5.29
MAC5A
PRP19B
PRL1
HISN2
XP_012083011.1/SECA1
XP_012071443.1/DCL4
HISN3
XP_012082764.1
SKI3
XP_012083359.1/JcDDDX37
XP_012084225.1
RRP41
MLP3.20
MUA22.5
T11H3.90
T2K10.14
RRP5
F5E6.14
EBP2
PWP2
RRP4
TOZ
XP_012083359.1
PES
ENP1
F4H5.19
XP_012068365.1/SKI2
BOP1
MDB19.11
NOC4
XP_020533333.1
T1J1.5
Q9LVF2
PPAN
XP_012071443.1
XP_012068365.1
XP_012083359.1/ABO6
T32M21-200
XP_012090372.1/RH7
XP_037493586.1
XP_012073544.1
ARP5
RIN1
XP_037497261.1/RECQL4B
XP_012071299.1
XP_020533333.1/CHR25
ARP4
Clusters:
RNA degradation by exosome
Exon-exon junction complex
Spliceosome
Chromatin remodeling and DNA repair
Ribosomal biogenesis
Histidine biosynthesis
