# Supplementary material for: DEAD-Box RNA Helicase Family in Physic Nut (Jatropha curcas L.): Structural Characterization and Response to Salinity
Source: Plants (Basel). 2024 Mar 21;13(6):905. doi: 10.3390/plants13060905 (PMC10974417; doi:10.3390/plants13060905)
Supplement: Supplementary file 1 [file plants-13-00905-s001.zip › Supp_Mat/Figures/Figure S6.pdf]

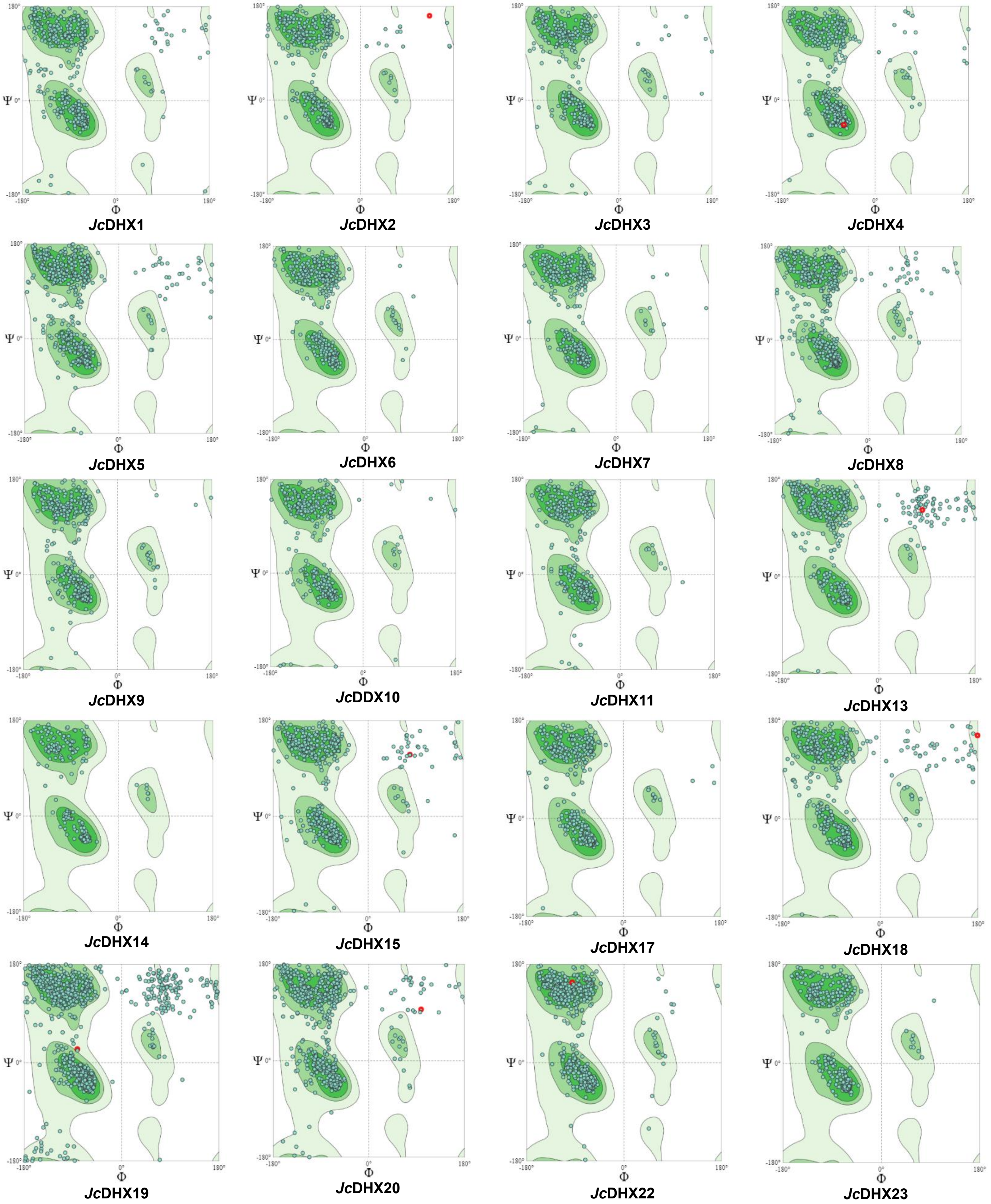
JcDHX1
JcDHX2
JcDHX3
JcDHX4
JcDHX5
JcDHX6
JcDHX7
JcDHX8
JcDHX9
JcDDX10
JcDHX11
JcDHX13
JcDHX14
JcDHX15
JcDHX17
JcDHX18
JcDHX19
JcDHX20
JcDHX22
JcDHX23
Φ
Ψ 0°
180°
-180°
0°

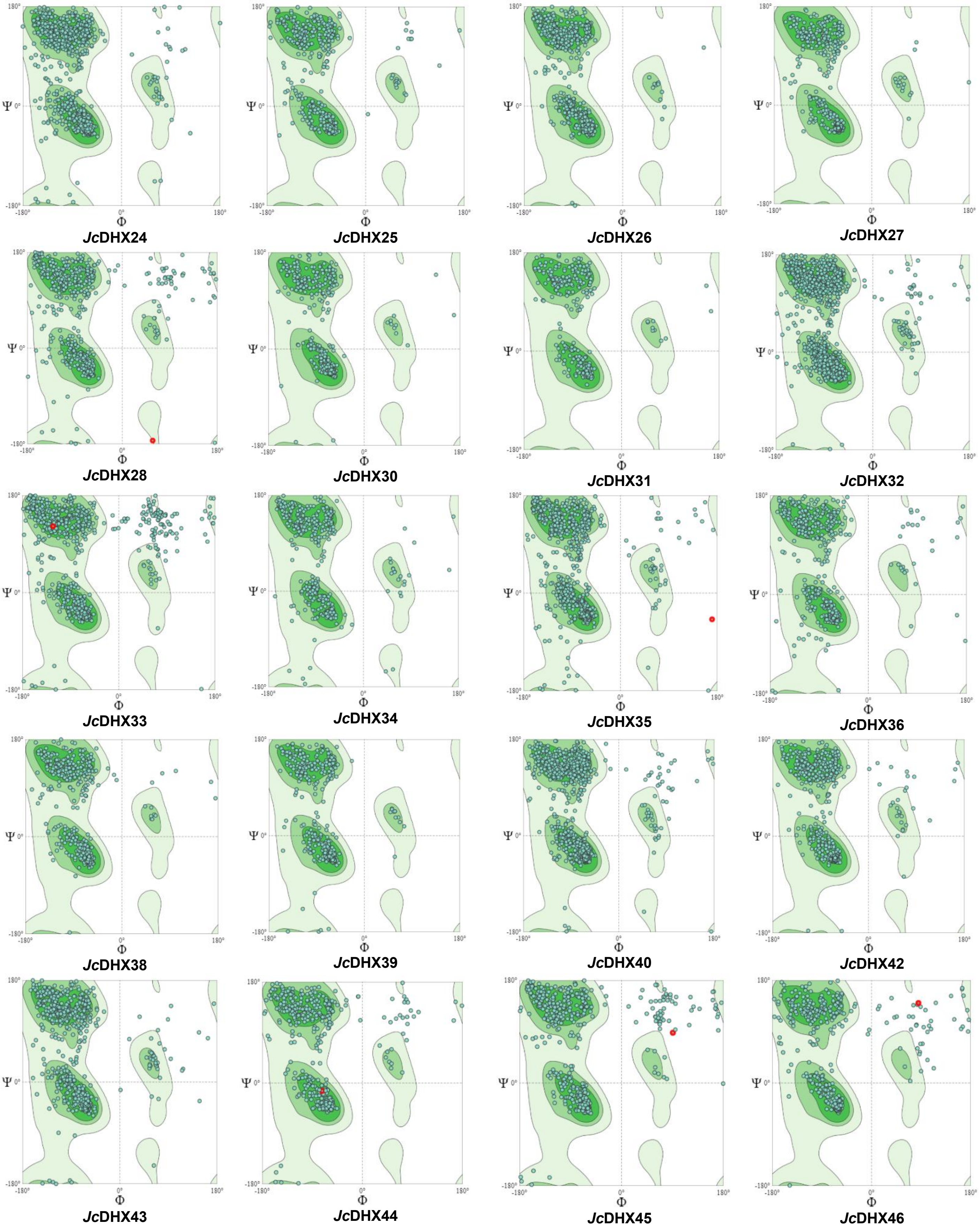

180°
Ψ 0°
-180°
-180°
0°
Φ
180°
JcDHX24
JcDHX25
JcDHX26
JcDHX27
JcDHX28
JcDHX30
JcDHX31
JcDHX32
JcDHX33
JcDHX34
JcDHX35
JcDHX36
JcDHX38
JcDHX39
JcDHX40
JcDHX42
JcDHX43
JcDHX44
JcDHX45
JcDHX46

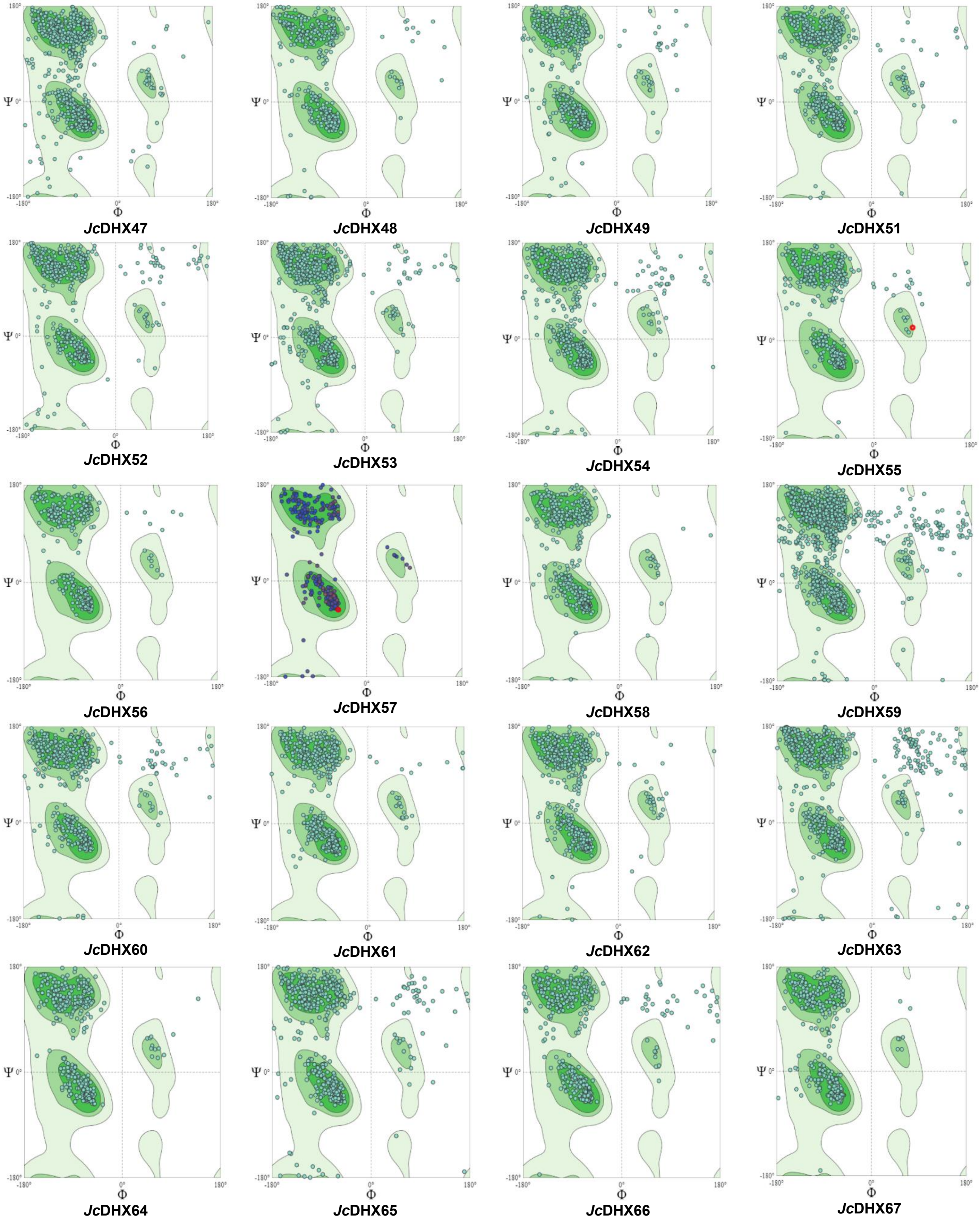

JcDHX47
JcDHX48
JcDHX49
JcDHX51
JcDHX52
JcDHX53
JcDHX54
JcDHX55
JcDHX56
JcDHX57
JcDHX58
JcDHX59
JcDHX60
JcDHX61
JcDHX62
JcDHX63
JcDHX64
JcDHX65
JcDHX66
JcDHX67

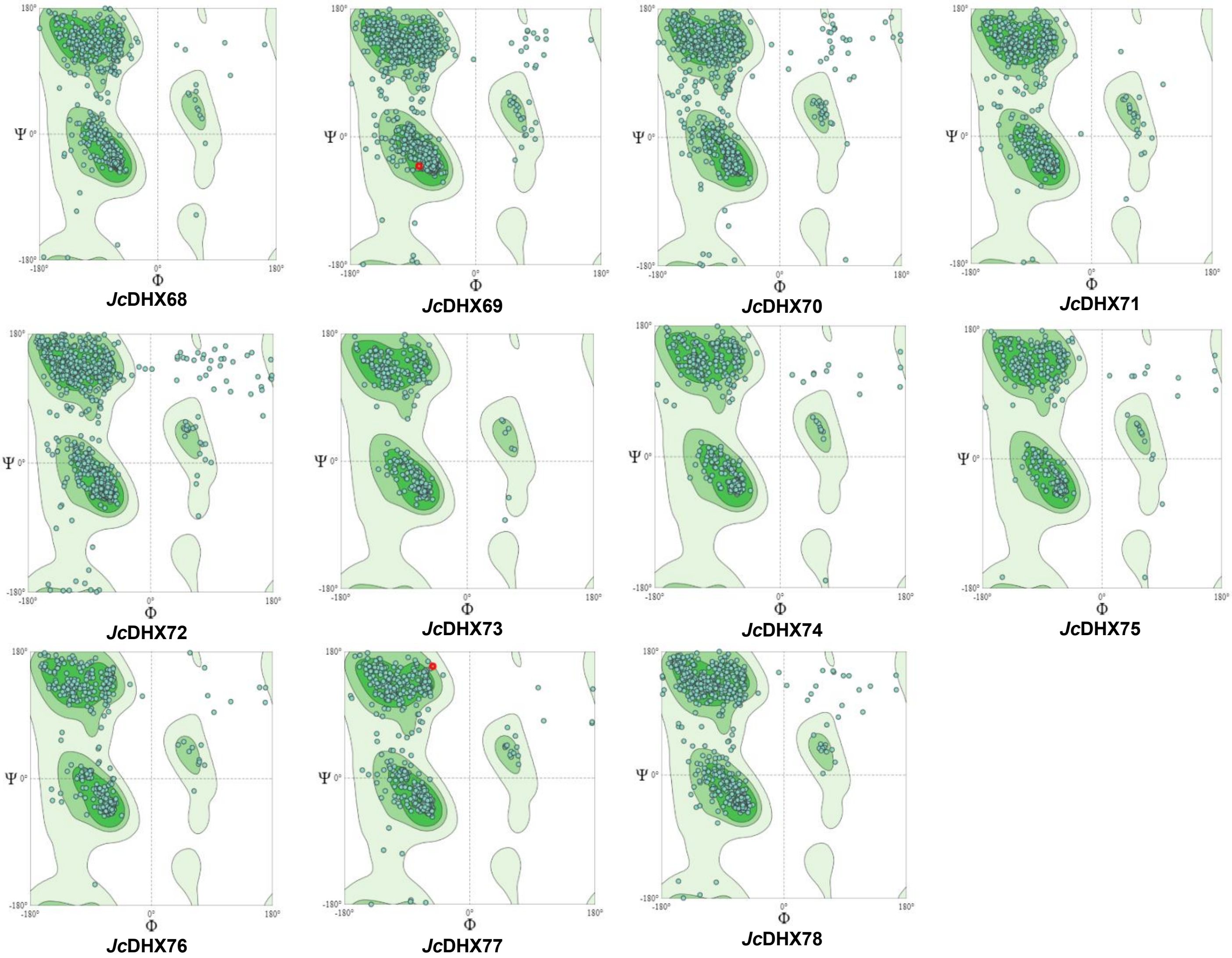
JcDHX68
JcDHX69
JcDHX70
JcDHX71
JcDHX72
JcDHX73
JcDHX74
JcDHX75
JcDHX76
JcDHX77
JcDHX78
