# Supplementary material for: DEAD-Box RNA Helicase Family in Physic Nut (Jatropha curcas L.): Structural Characterization and Response to Salinity
Source: Plants (Basel). 2024 Mar 21;13(6):905. doi: 10.3390/plants13060905 (PMC10974417; doi:10.3390/plants13060905)
Supplement: Supplementary file 1 [file plants-13-00905-s001.zip › Supp_Mat/Figures/Figure S7.pdf]

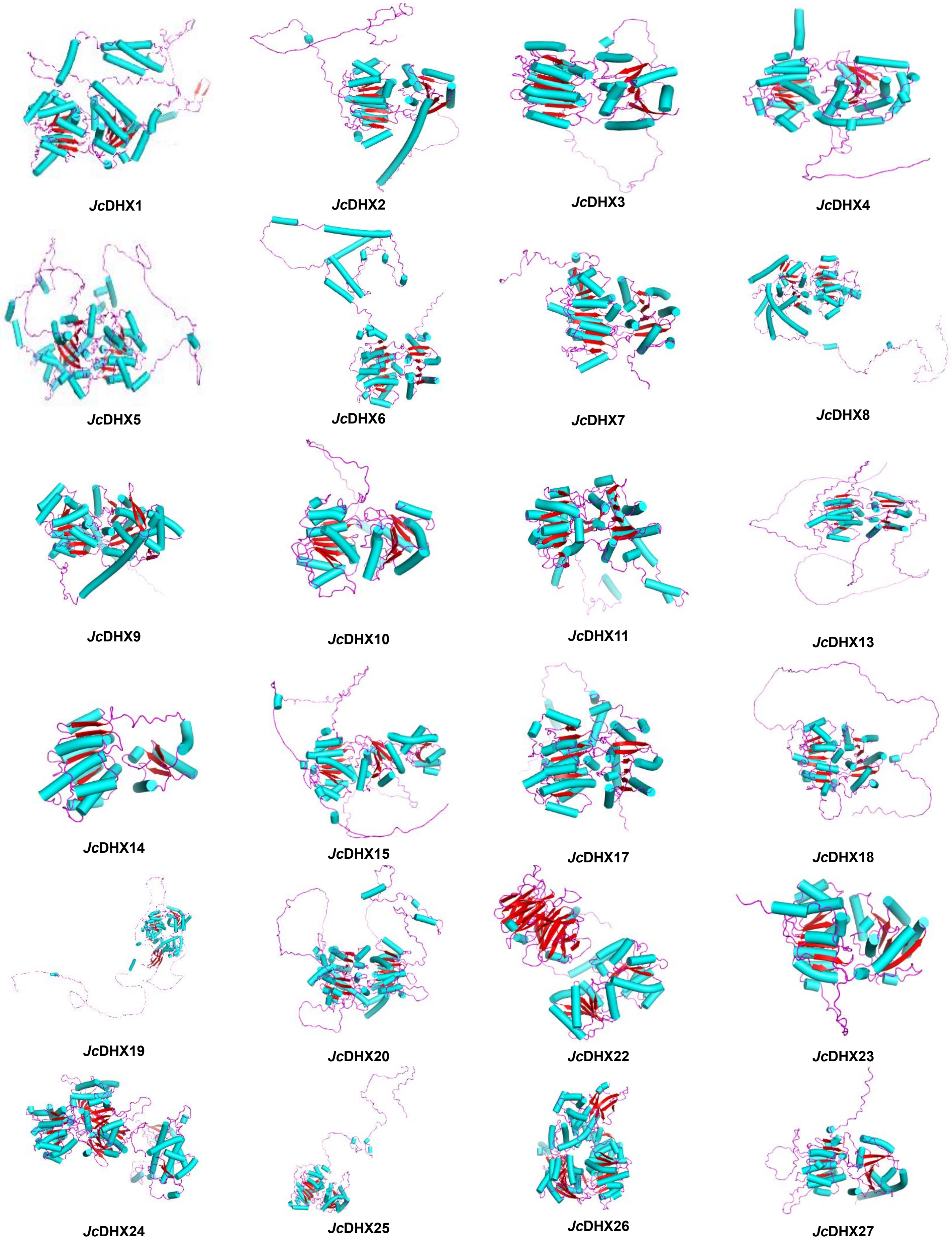
*Jc*DHX1
*Jc*DHX2
*Jc*DHX3
*Jc*DHX4
*Jc*DHX5
*Jc*DHX6
*Jc*DHX7
*Jc*DHX8
*Jc*DHX9
*Jc*DHX10
*Jc*DHX11
*Jc*DHX13
*Jc*DHX14
*Jc*DHX15
*Jc*DHX17
*Jc*DHX18
*Jc*DHX19
*Jc*DHX20
*Jc*DHX22
*Jc*DHX23
*Jc*DHX24
*Jc*DHX25
*Jc*DHX26
*Jc*DHX27

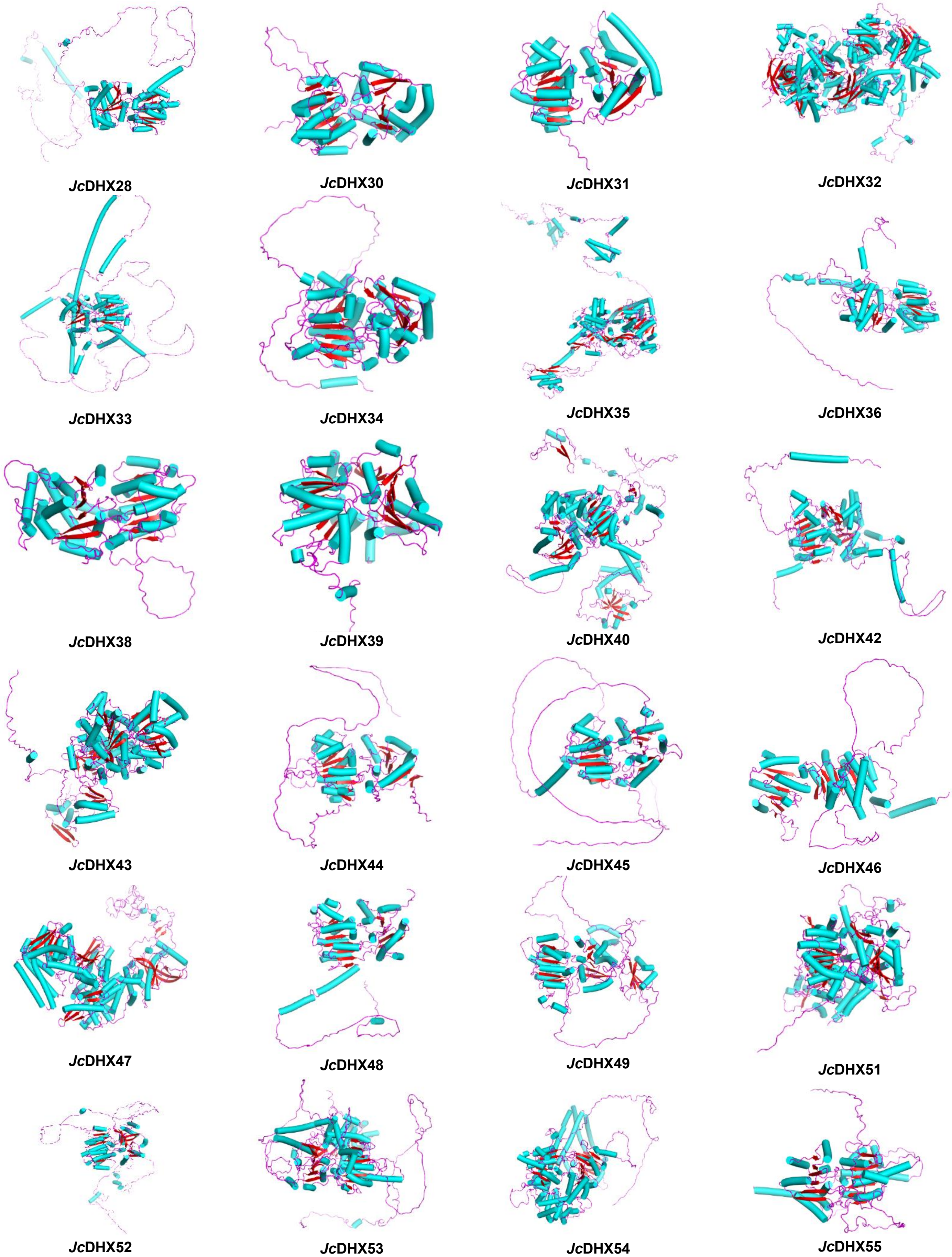
*Jc*DHX28
*Jc*DHX30
*Jc*DHX31
*Jc*DHX32
*Jc*DHX33
*Jc*DHX34
*Jc*DHX35
*Jc*DHX36
*Jc*DHX38
*Jc*DHX39
*Jc*DHX40
*Jc*DHX42
*Jc*DHX43
*Jc*DHX44
*Jc*DHX45
*Jc*DHX46
*Jc*DHX47
*Jc*DHX48
*Jc*DHX49
*Jc*DHX51
*Jc*DHX52
*Jc*DHX53
*Jc*DHX54
*Jc*DHX55

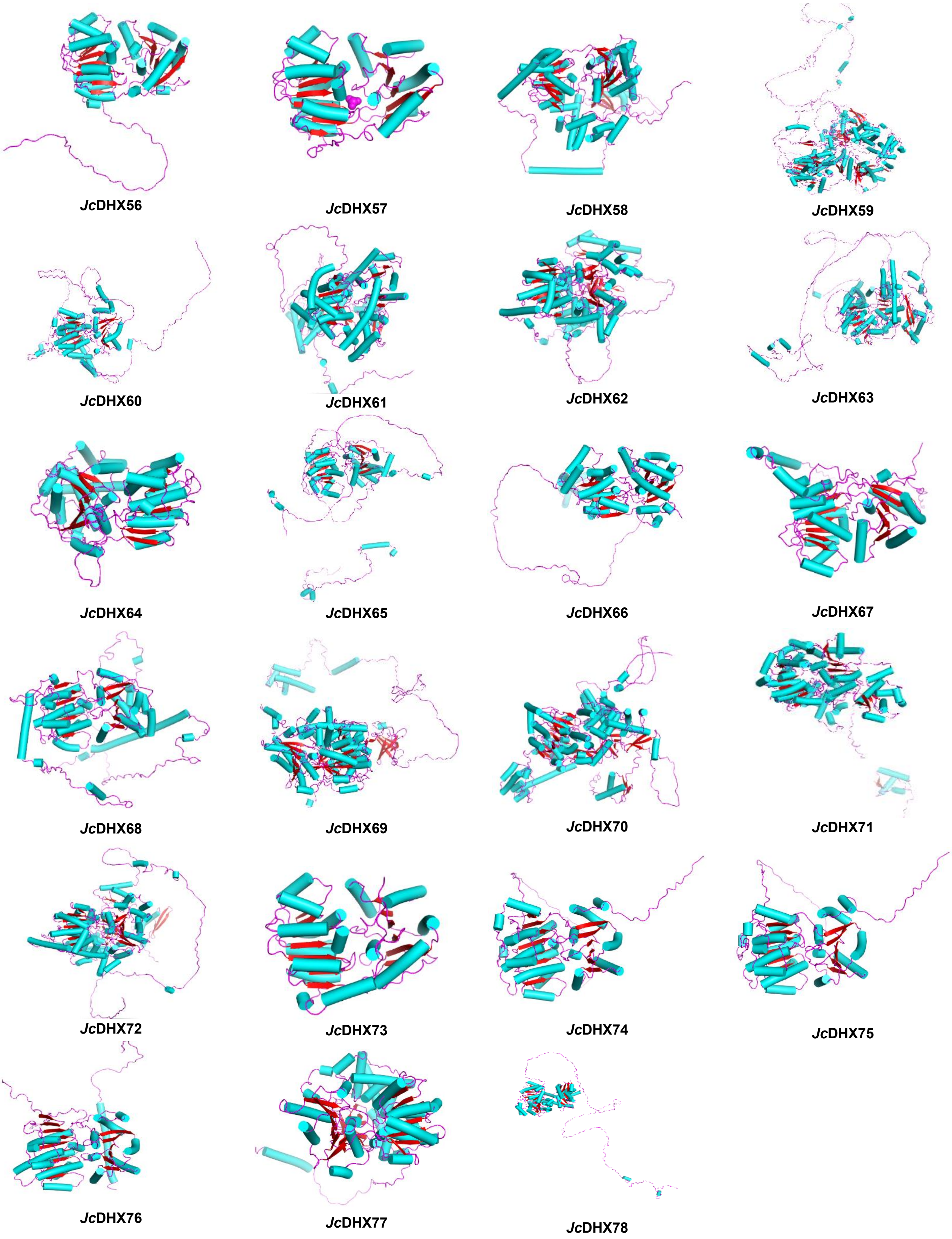
*Jc*DHX56
*Jc*DHX57
*Jc*DHX58
*Jc*DHX59
*Jc*DHX60
*Jc*DHX61
*Jc*DHX62
*Jc*DHX63
*Jc*DHX64
*Jc*DHX65
*Jc*DHX66
*Jc*DHX67
*Jc*DHX68
*Jc*DHX69
*Jc*DHX70
*Jc*DHX71
*Jc*DHX72
*Jc*DHX73
*Jc*DHX74
*Jc*DHX75
*Jc*DHX76
*Jc*DHX77
*Jc*DHX78
