# Supplementary material for: DEAD-Box RNA Helicase Family in Physic Nut (Jatropha curcas L.): Structural Characterization and Response to Salinity
Source: Plants (Basel). 2024 Mar 21;13(6):905. doi: 10.3390/plants13060905 (PMC10974417; doi:10.3390/plants13060905)
Supplement: Supplementary file 1 [file plants-13-00905-s001.zip › Supp_Mat/Figures/Figure S8.pdf]

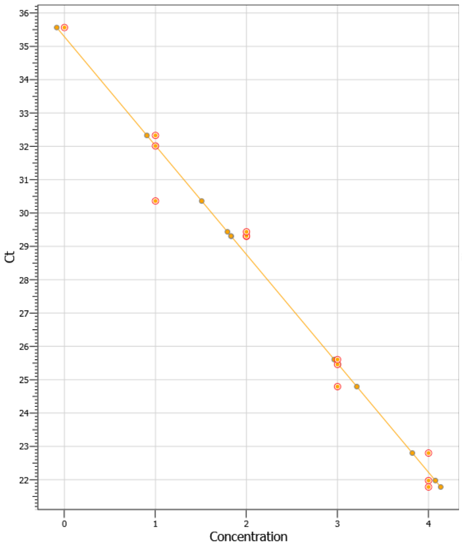

Assay:**7374 - SYBR** Intercept:**35.29** Slope:**-3.27** Error:**0.023** Correlation:**-0.989** Efficiency:**102.34**

DN43295_g1_i2

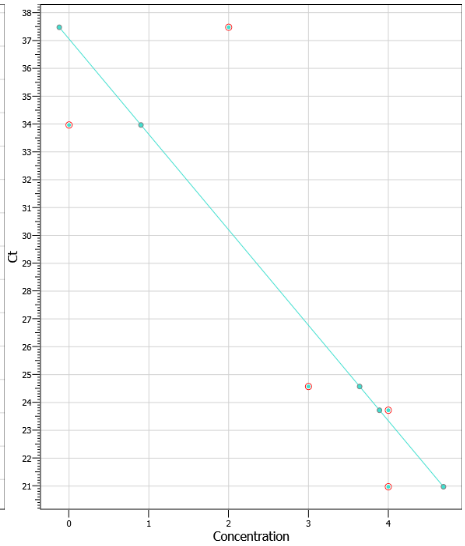

Assay:**DN43259_g2_i2 - SYBR** Intercept:**37.06** Slope:**-3.43** Error:**0.136** Correlation:**-0.802** Efficiency:**95.76**

DN43259_g2_i2

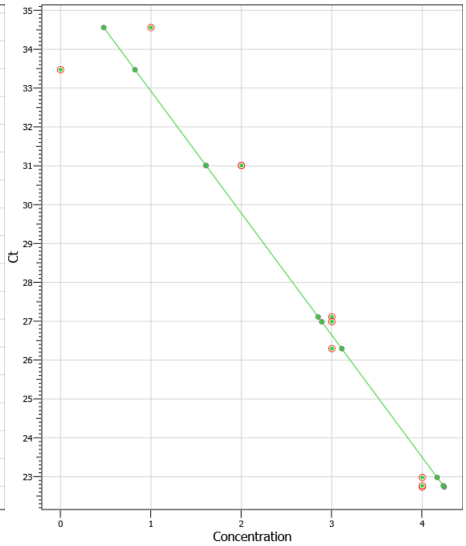

Assay:**7980 - SYBR** Intercept:**36.06** Slope:**-3.14** Error:**0.043** Correlation:**-0.959** Efficiency:**108.19**

DN39804_g2_i2

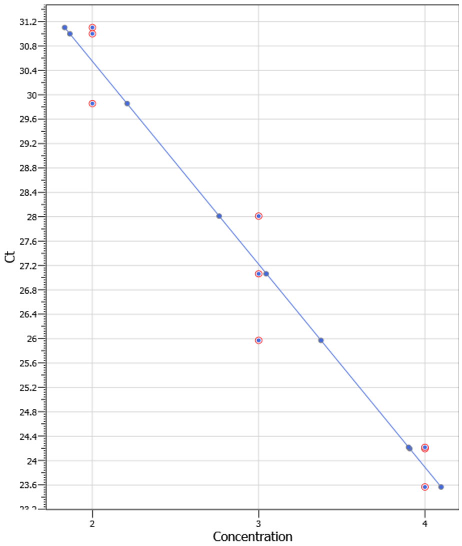

Assay:**8586 - SYBR** Intercept:**37.21** Slope:**-3.33** Error:**0.023** Correlation:**-0.975** Efficiency:**99.72**

DN43635_g1_i2

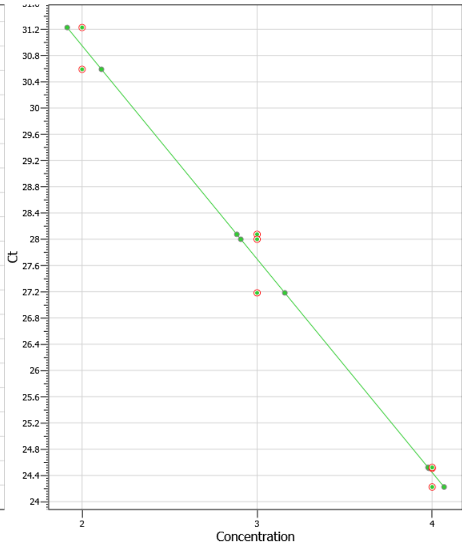

Assay:**A1A2 - SYBR** Intercept:**37.46** Slope:**-3.25** Error:**0.011** Correlation:**-0.993** Efficiency:**102.98**

DN36330_g1_i1

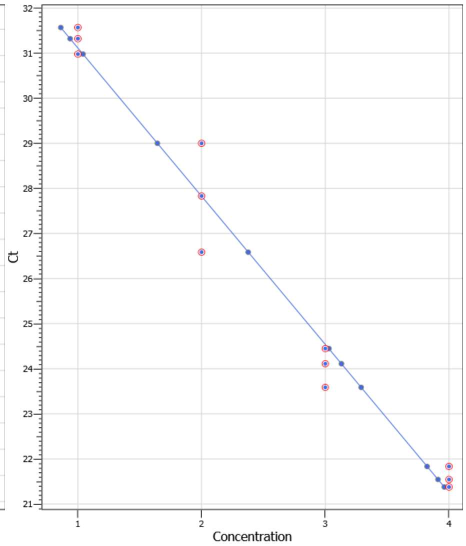

Assay:**B1B2 - SYBR** Intercept:**34.4** Slope:**-3.29** Error:**0.024** Correlation:**-0.986** Efficiency:**101.48**

DN97737_g2_i1

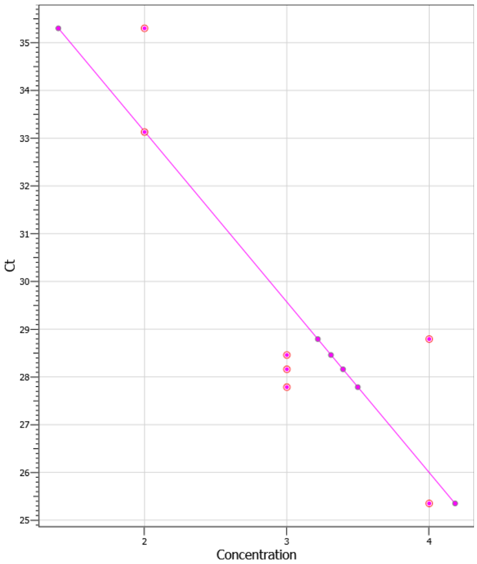

Assay:**DN40374_g1_i1 - SYBR** Intercept:**40.28** Slope:**-3.57** Error:**0.056** Correlation:**-0.852** Efficiency:**90.52**

DN40374_g1_i2

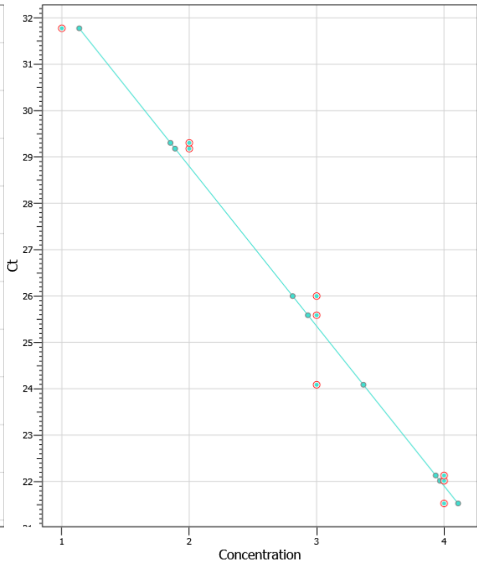

Assay:**8990 - SYBR** Intercept:**35.69** Slope:**-3.45** Error:**0.022** Correlation:**-0.987** Efficiency:**95.05**

DN62351_g1_i1

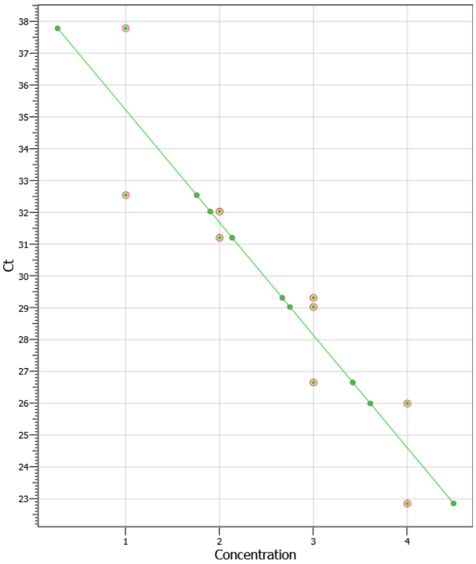

Assay:**DN7180_g2_i1 - SYBR** Intercept:**38.76** Slope:**-3.54** Error:**0.054** Correlation:**-0.919** Efficiency:**91.69**

DN7180_g1_i1

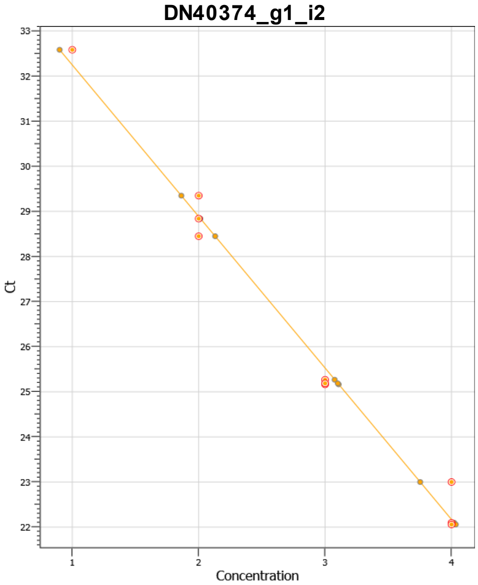

Assay:**DN85598_g1_i1 - SYBR** Intercept:**35.6** Slope:**-3.36** Error:**0.015** Correlation:**-0.993** Efficiency:**98.48**

DN85598_g1_i1

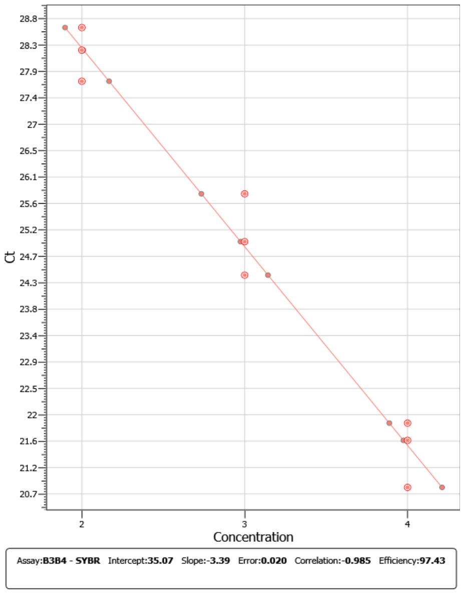

DN43391_g1_i3

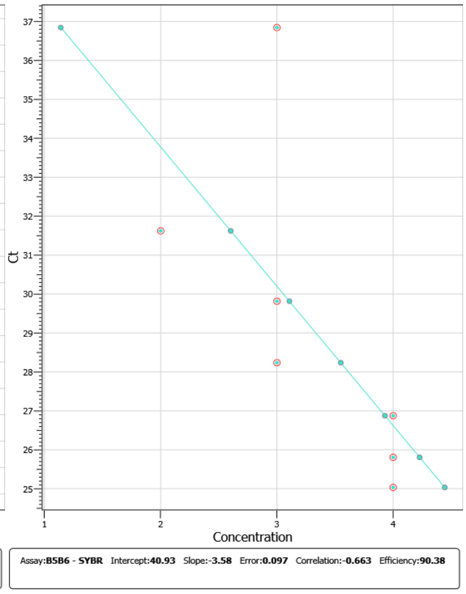

DN39804_g2_i1

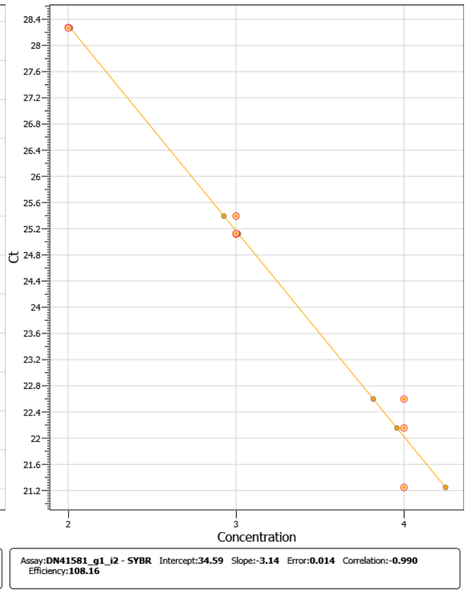

DN41581_g1_i2
