# Supplementary figures and images for: DEAD-Box RNA Helicase Family in Physic Nut (Jatropha curcas L.): Structural Characterization and Response to Salinity
Source: Plants (Basel). 2024 Mar 21;13(6):905. doi: 10.3390/plants13060905 (PMC10974417; doi:10.3390/plants13060905)

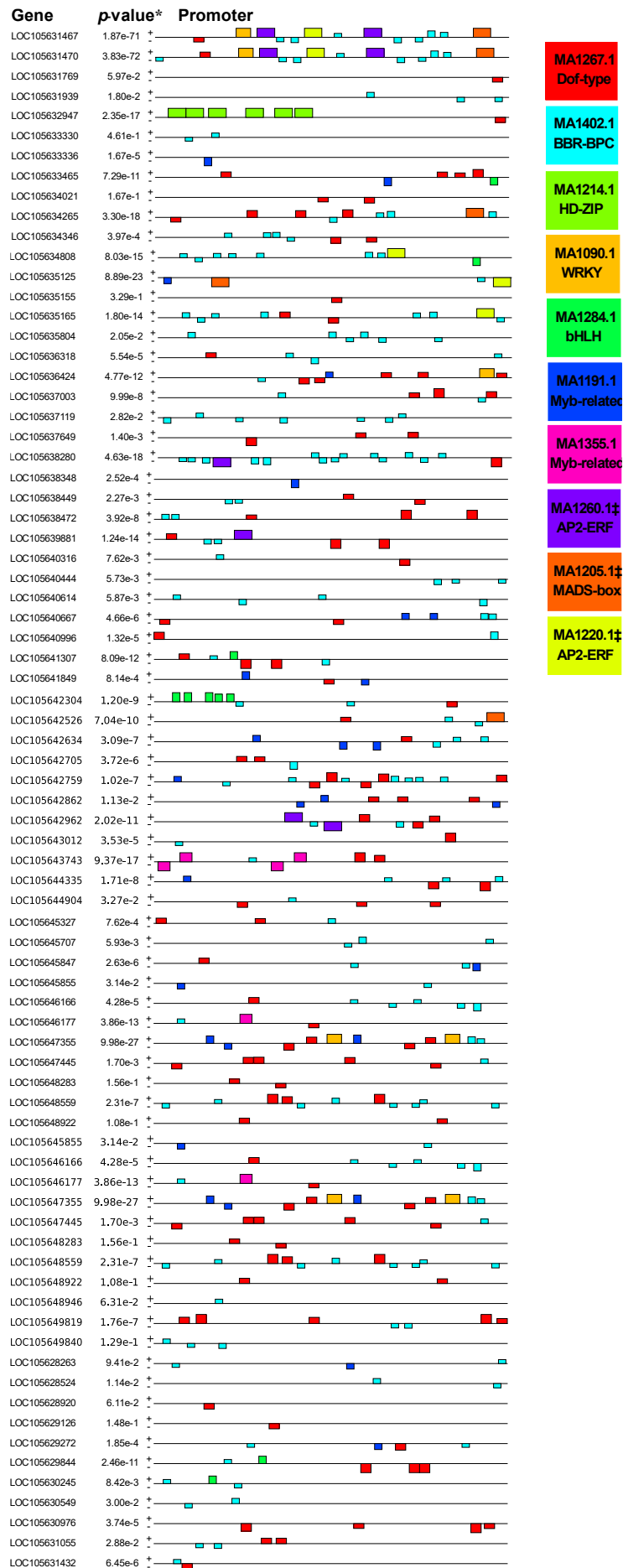

Supplement: Supplementary file 1 [file plants-13-00905-s001.zip › Supp_Mat/Figures/Figure S2.pdf]

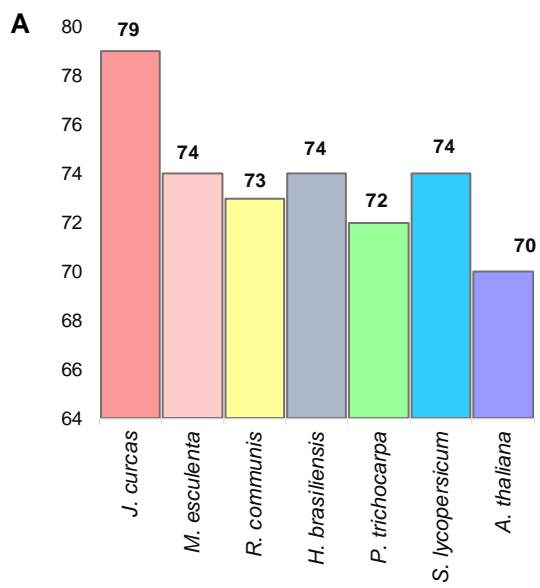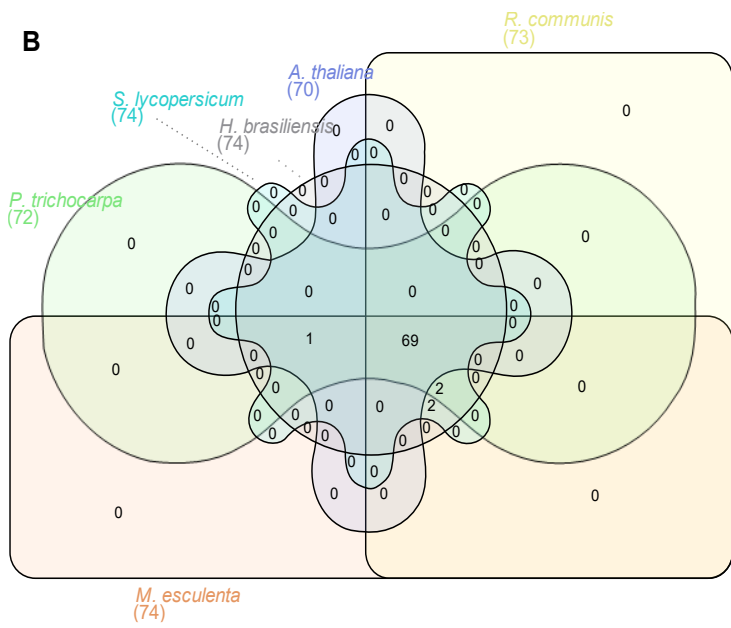

Supplement: Supplementary file 1 [file plants-13-00905-s001.zip › Supp_Mat/Figures/Figure S3.pdf]

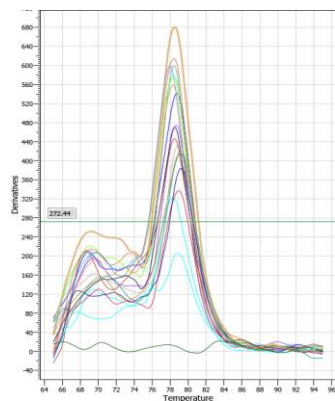

DN43295\_g1\_i2

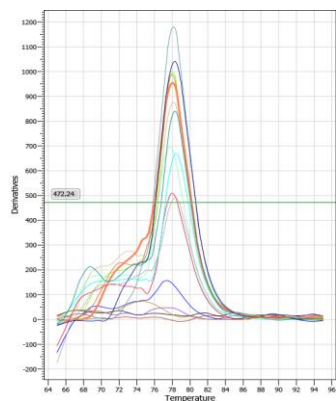

DN43259\_g2\_i2

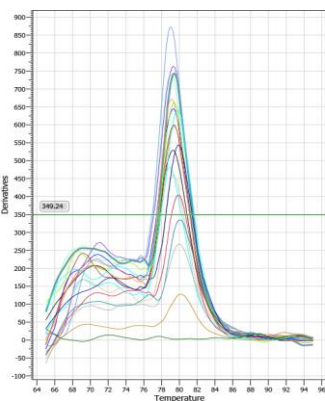

DN39804\_g2\_i2

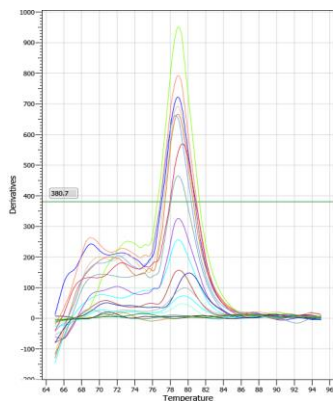

DN43635\_g1\_i2

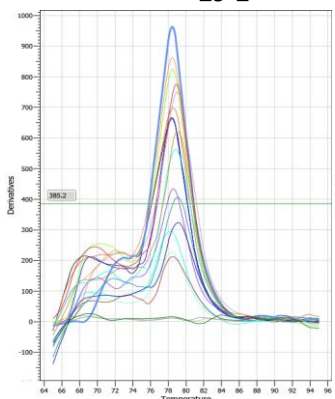

DN36330\_g1\_i1

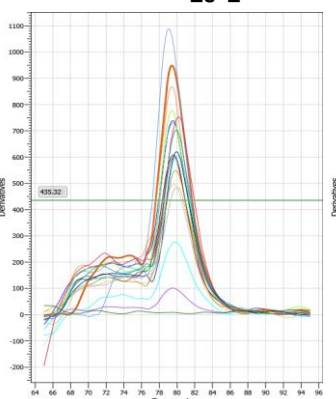

DN97737\_g2\_i1

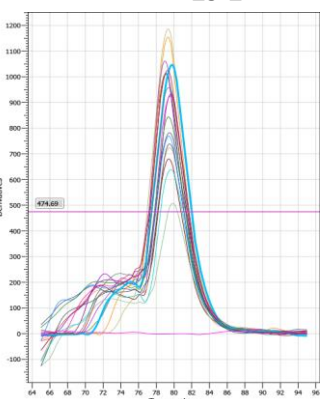

DN43391\_g1\_i3

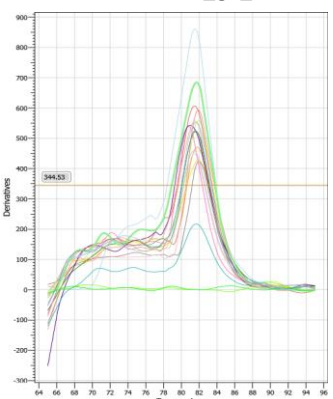

DN39804\_g2\_i1

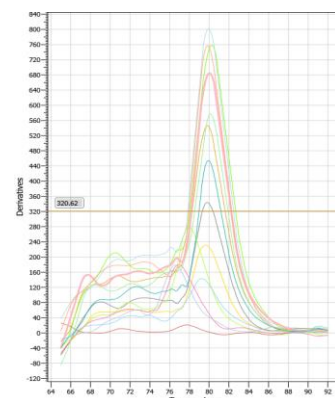

DN41581\_g1\_i2

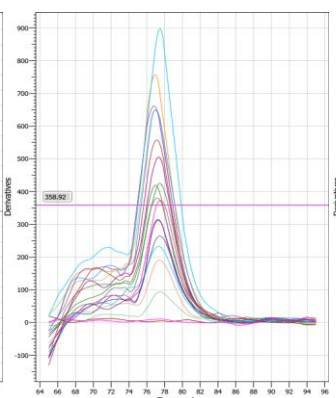

DN40374\_g1\_i2

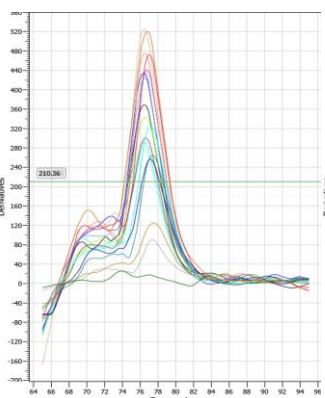

DN62351\_g1\_i1

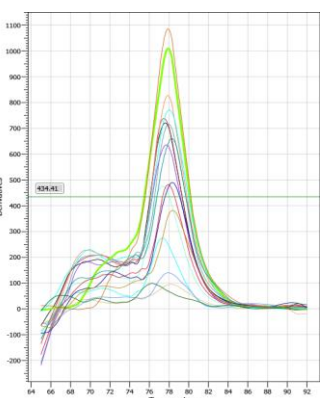

DN7180\_g1\_i1

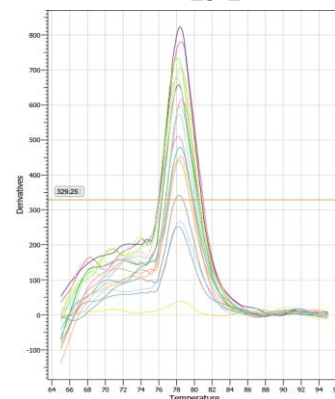

DN85598\_g1\_i1

Supplement: Supplementary file 1 [file plants-13-00905-s001.zip › Supp_Mat/Figures/Figure S9.pdf]
