# Supplementary material for: DEAD-Box RNA Helicase Family in Physic Nut (Jatropha curcas L.): Structural Characterization and Response to Salinity
Source: Plants (Basel). 2024 Mar 21;13(6):905. doi: 10.3390/plants13060905 (PMC10974417; doi:10.3390/plants13060905)
Supplement: Supplementary file 1 [file Figure S4.pdf › Supp_Mat/Figures/Figure S4.pdf]

```
                         10        20        30        40        50        60        70        80
XP_012090812.1  --------------------------------------------------------------------------------
XP_012086968.1  --------------------------------------------------------------------------------
XP_037493586.1  --------------------------------------------------------------------------------
XP_037493005.1  MCGDATNWDEDAYRESILKEREIQTLTVFRTAWAPSSDVSLNTVVVASSDGSIASYSISSCISNLPLGFSNAKAQQIEPT
XP_012088793.1  --------------------------------------------------------------------------------
XP_012071299.1  --------------------------------------------------------------------------------
XP_012070805.1  --------------------------------------------------------------------------------
XP_012083226.1  --------------------------------------------------------------------------------
XP_012065797.1  --------------------------------------------------------------------------------
XP_012087374.1  --------------------------------------------------------------------------------
XP_012074968.1  --------------------------------------------------------------------------------
XP_012082496.1  --------------------------------------------------------------------------------
XP_012073200.1  --------------------------------------------------------------------------------
XP_012068438.1  --------------------------------------------------------------------------------
XP_012090372.1  --------------------------------------------------------------------------------
XP_012087359.1  --------------------------------------------------------------------------------
XP_012083429.1  --------------------------------------------------------------------------------
XP_012090844.1  --------------------------------------------------------------------------------
XP_020534206.1  --------------------------------------------------------------------------------
XP_012084225.1  --------------------------------------------------------------------------------
XP_037493126.1  --------------------------------------------------------------------------------
XP_020537458.2  --------------------------------------------------------------------------------
XP_012077644.1  --------------------------------------------------------------------------------
XP_012092036.1  --------------------------------------------------------------------------------
XP_012073558.1  --------MEDVKHKSRKEDSEKKEEDPKRSHRDKERERDKERNGERHRDREKRDRESRRSEREKSTDSDDKYERERDKD
XP_012082917.1  --------------------------------------------------------------------------------
XP_012086018.1  --------------------------------------------------------------------------------
XP_037493732.1  --------------------------------------------------------------------------------
XP_037497718.1  --------------------------------------------------------------------------------
XP_037492156.1  --------------------------------------------------------------------------------
XP_020540476.1  --------------------------------------------------------------------------------
```

```
XP_012076586.1    --------------------------------------------------------------------------------
XP_012084332.2    --------------------------------------------------------------------------------
XP_012065043.1    --------------------------------------------------------------------------------
XP_012067404.1    --------------------------------------------------------------------------------
XP_012079452.1    --------------------------------------------------------------------------------
XP_037497717.1    --------------------------------------------------------------------------------
XP_012072496.1    --------------------------------------------------------------------------------
XP_012073120.1    --------------------------------------------------------------------------------
XP_012069334.1    --------------------------------------------------------------------------------
XP_037494154.1    --------------------------------------------------------------------------------
XP_020533941.1    --------------------------------------------------------------------------------

                            90        100       110       120       130       140       150       160
XP_012090812.1    --------------------------------------------------------------------------------
XP_012086968.1    --------------------------------------------------------------------------------
XP_037493586.1    --------------------------------------------------MSTSILLQHSRTISGMLHTRIFVRFMGGGP
XP_037493005.1    AFLQGHDGPAYDLKFYGNGEDALLLSCGDDGRIRGWRWKEFSQLEAPISLKGNHIQPVVDLVNPQHKGPWGALSPIPENN
XP_012088793.1    --------------------------------------------------------------------------------
XP_012071299.1    --------------------------------------------------------------------------------
XP_012070805.1    --------------------------------------------------------------------------------
XP_012083226.1    --------------------------------------------------MSIKLPLQ--------------LRFLG---
XP_012065797.1    --------------------------------------------------------------------------------
XP_012087374.1    --------------------------------------------------------------------------------
XP_012074968.1    ---------------------------------------------------------------------MKRSIDDIGAN
XP_012082496.1    --------------------------------------------------------------------------------
XP_012073200.1    --------------------------------------------------------------------------------
XP_012068438.1    --------------------------------------------------------------------------------
XP_012090372.1    --------------------------------------------------------------------------------
XP_012087359.1    --------------------------------------------------------------------------------
XP_012083429.1    --------------------------------------------------------------------------------
XP_012090844.1    --------------------------------------------------------------------------------
XP_020534206.1    --------------------------------------------------------------------------------
XP_012084225.1    --------------------------------------------------------------------------------
XP_037493126.1    --------------------------------------------------------------------------------
```

```
XP_020537458.2   --------------------------------------------------------------------------------
XP_012077644.1   --------------------------------------------------------------------------------
XP_012092036.1   --------------------------------------------------------------------------------
XP_012073558.1   RDKHRDRDKDRRSRDDERERTKDKSRKRDRDSERDEKRERARDRDKERERDRERERERDREKREREREERERERDKERER
XP_012082917.1   --------------------------------------------------------------------------------
XP_012086018.1   --------------------------------------------------------------------------------
XP_037493732.1   --------------------------------------------------------------------------------
XP_037497718.1   --------------------------------------------------------------------------------
XP_037492156.1   --------------------------------------------------------------------------------
XP_020540476.1   --------------------------------------------------------------------------------
XP_012076586.1   --------------------------------------------------------------------------------
XP_012084332.2   --------------------------------------------------------------------------------
XP_012065043.1   --------------------------------------------------------------------------------
XP_012067404.1   --------------------------------------------------------------------------------
XP_012079452.1   --------------------------------------------------------------------------------
XP_037497717.1   --------------------------------------------------------------------------------
XP_012072496.1   --------------------------------------------------------------------------------
XP_012073120.1   --------------------------------------------------------------------------------
XP_012069334.1   --------------------------------------------------------------------------------
XP_037494154.1   --------------------------------------------------------------------------------
XP_020533941.1   --------------------------------------------------------------------------------

                           170       180       190       200       210       220       230       240
XP_012090812.1   ---------------MMSITLRRSTSQAPKRVLAALTS------------------------------------------
XP_012086968.1   --------------------------------------------------------------------------------
XP_037493586.1   RTFPGGLNKWQWKRLHEKRAKEKEKRLLDQEKQLYEARIRSNIRAKLAGK-PDPNLNPDTNTTSYSPMSPENHIKALADR
XP_037493005.1   AIAVDTQGGCIFSAAGDSCAYCWDVETSKIRMVFKGHFDYLHCIVPRNSTNQIITGSEDGTARIWDCKSGKCIQVIGSAK
XP_012088793.1   --------MASSSVIGVSSIFHTPSVELSARRTTTLPP------------------------------------------
XP_012071299.1   --------------------------------------------------------------------------------
XP_012070805.1   --------------------------------------------------------------------------------
XP_012083226.1   -------PSFSATRLPPMKSLLS---VTSSRGVPILSRIFP-FRLKYVGFGPYPNSQVGVRRFSTRPFRPRHGSGSQFRR
XP_012065797.1   --------------------------------------------------------------------------------
XP_012087374.1   --------------------------------------------------------------------------------
XP_012074968.1   SNNKNGSKPVFLTKAQREQLALERRQEEIQQQKLRQQQLLAQIRSSDPNNTNQKPSDSERRDRDKDRERDRDRDRDRSSR
```

```
XP_012082496.1  --------------------------------------------------------------------------------
XP_012073200.1  --------------------------------------------------------------------------------
XP_012068438.1  --------------------------------------------------------------------------------
XP_012090372.1  --------------MPSIALANNSSDSKDKKERKMKKR------------------------------------------
XP_012087359.1  --------------------------------------------------------------------------------
XP_012083429.1  --------------------------------------------------------------------------------
XP_012090844.1  ------------MRTSWADSVANSASDSVESVNSGNNG-----QPRPTRATYIPPHLRNRAPSNDVPASSS---------
XP_020534206.1  --------------------------------------------------------------------------------
XP_012084225.1  -------MLTIAPPHPLSSFRLQITDSHSQSHRGVVFDYVSKSGAKPFALVRAACSSTRLNTPFQSVTELADGGGVCSHS
XP_037493126.1  ---------MSYVPPHLRNSSSHATTATSTTIINTTTT------------------------------------------
XP_020537458.2  ----------------MNPYDHRYS--DPESYRRRRSDLMGPPQPVGPPVAGVPYGRSGPVPYGGPPPPA----------
XP_012077644.1  --------------------------------------------------------------------------------
XP_012092036.1  --------------------------------------------------------------------------------
XP_012073558.1  RERERERRERERbusEEQRERERDRERERRDREREKERERERRTREKEKHREVNSDDNSDDDSREHDKKRRRRDDDDYKDRQI
XP_012082917.1  --------------MSKRKFGFEGFGINRQTTYNFER------------------SQAPQRLYVPPSSRHGHDNYEDTDL
XP_012086018.1  --------------------------------------------------------------------------------
XP_037493732.1  ----------MSTRTSWADLAANSAAENTSAGASANNGNIGTTSAVPSRATYVPPHLRNRAPSSDPPAAP----------
XP_037497718.1  ----------------MSRYDSRSG--DPTSYRDRRSD------------------------------------------
XP_037492156.1  --------------------------------------------------------------------------------
XP_020540476.1  --------------------------------------------------------------------------------
XP_012076586.1  --------------------------------------------------------------------------------
XP_012084332.2  ---------MAAAEPAPSSLGPRYAPEDPTLPKPWMGLIDGSTGRLYYWNPETNITQYEKPASLPPPLPPGPPPPASTPK
XP_012065043.1  --------------------------------------------------------------------------------
XP_012067404.1  --------------MEVANSDNHCKNSAAVVSFDQFTG------------------------------------------
XP_012079452.1  --------------------------------------------------------------------------------
XP_037497717.1  ----------------MSRYDSRSG--DPTSYRDRRSD------------------------------------------
XP_012072496.1  ---------------MAATAGPRYAPEDPTLPKPWRGLVDGKTGYLYFWNPETNVTQYERPTAPAHPLKS----------
XP_012073120.1  --------------------------------------------------------------------------------
XP_012069334.1  ---------MVKKHQEPALVGGEQFGEQTDKKKKMKKK------------------------------------------
XP_037494154.1  --------------------------------------------------------------------------------
XP_020533941.1  --------------------------------------------------------------------------------

                         250           260           270           280           290           300           310           320
XP_012090812.1  --------------------------------------------------------------------------------
```

```
XP_012086968.1   --------------------------------------------------------------------------------
XP_037493586.1   FMKEGAEDLWNENDGPLKSSLAK---------------------------------------------------------
XP_037493005.1   DKKLKRSLCVSCIALDSSESWLACSNGQSLSVWN----------------------------------------------
XP_012088793.1   --------------------------------------------------------------------------------
XP_012071299.1   --------------------------------------------------------------------------------
XP_012070805.1   --------------------------------------------------------------------------------
XP_012083226.1   GERDRGD--VGASKSLIEDEAEL---------------------------------------------------------
XP_012065797.1   --------------------------------------------------------------------------------
XP_012087374.1   --------------------------------------------------------------------------------
XP_012074968.1   RDRDREREREEEAKARERARLEKLAEREREKELD----------------------------------------------
XP_012082496.1   --------------------------------------------------------------------------------
XP_012073200.1   --------------------------------------------------------------------------------
XP_012068438.1   --------------------------------------------------------------------------------
XP_012090372.1   --------------------------------------------------------------------------------
XP_012087359.1   --------------------------------------------------------------------------------
XP_012083429.1   --------------------------------------------------------------------------------
XP_012090844.1   --------------------------------------------------------------------------------
XP_020534206.1   --------------------------------------------------------------------------------
XP_012084225.1   PNK-----------------------------------------------------------------------------
XP_037493126.1   --------------------------------------------------------------------------------
XP_020537458.2   --------------------------------------------------------------------------------
XP_012077644.1   --------------------------------------------------------------------------------
XP_012092036.1   --------------------------------------------------------------------------------
XP_012073558.1   EL--------------------------------------SNSRSNRHRDDTEGSPRKKSSEDDSDKKEKKTREEELEDE
XP_012082917.1   DN--------------------------------------IDYEENDNAKDTS---------------------------
XP_012086018.1   --------------------------------------------------------------------------------
XP_037493732.1   --------------------------------------------------------------------------------
XP_037497718.1   --------------------------------------------------------------------------------
XP_037492156.1   --------------------------------------------------------------------------------
XP_020540476.1   --------------------------------------------------------------------------------
XP_012076586.1   --------------------------------------------------------------------------------
XP_012084332.2   LAQIPMAHSAAQPNGLVVQIAQQIPQTTVQQGQQISQLPQQAQATQQITQVTQQLGQQSNQLPQQQGQLMPQQQSLPVSQ
XP_012065043.1   --------------------------------------------------------------------------------
```

```
XP_012067404.1    --------------------------------------------------------------------------------
XP_012079452.1    --------------------------------------------------------------------------------
XP_037497717.1    --------------------------------------------------------------------------------
XP_012072496.1    --------------------------------------------------------------------------SMPISS
XP_012073120.1    --------------------------------------------------------------------------------
XP_012069334.1    --------------------------------------------------------------------------------
XP_037494154.1    --------------------------------------------------------------------------------
XP_020533941.1    --------------------------------------------------------------------------------

                           330       340       350       360       370       380       390       400
XP_012090812.1    --------------------------------------------------------------------------------
XP_012086968.1    --------------------------------------------------------------------------------
XP_037493586.1    ------------------------------------------------SSERPELSGPNQRPTSINSPIDLRKLISGGVN
XP_037493005.1    ---------------------------------------------------------LPASECVSRSSCPASIQDVIFDD
XP_012088793.1    --------------------------------------------------------------------------------
XP_012071299.1    --------------------------------------------------------------------------------
XP_012070805.1    --------------------------------------------------------------------------------
XP_012083226.1    ------------------------------------------------SDWVSELSTSSFRGRVTDGESDS-DISRDRVS
XP_012065797.1    --------------------------------------------------------------------------------
XP_012087374.1    --------------------------------------------------------------------------------
XP_012074968.1    ---------------------------------------------------------SIKEQYLGSKKPKKRVIKPSEKF
XP_012082496.1    --------------------------------------------------------------------------------
XP_012073200.1    --------------------------------------------------------------------------------
XP_012068438.1    --------------------------------------------------------------------------------
XP_012090372.1    --------------------------------------------------------------------------------
XP_012087359.1    --------------------------------------------------------------------------------
XP_012083429.1    --------------------------------------------------------------------------------
XP_012090844.1    --------------------------------------------------------------------------------
XP_020534206.1    --------------------------------------------------------------------------------
XP_012084225.1    -----------------------------------------------------------EDEDATSGIANPGAVTKTANP
XP_037493126.1    --------------------------------------------------------------------------------
XP_020537458.2    --------------------------------------------------------------------------------
XP_012077644.1    ------------------------------------------MAADSPLSSQPPLSSKSSNKKGKGKRQRKPHKPELDRV
XP_012092036.1    --------------------------------------------------------------------------------
```

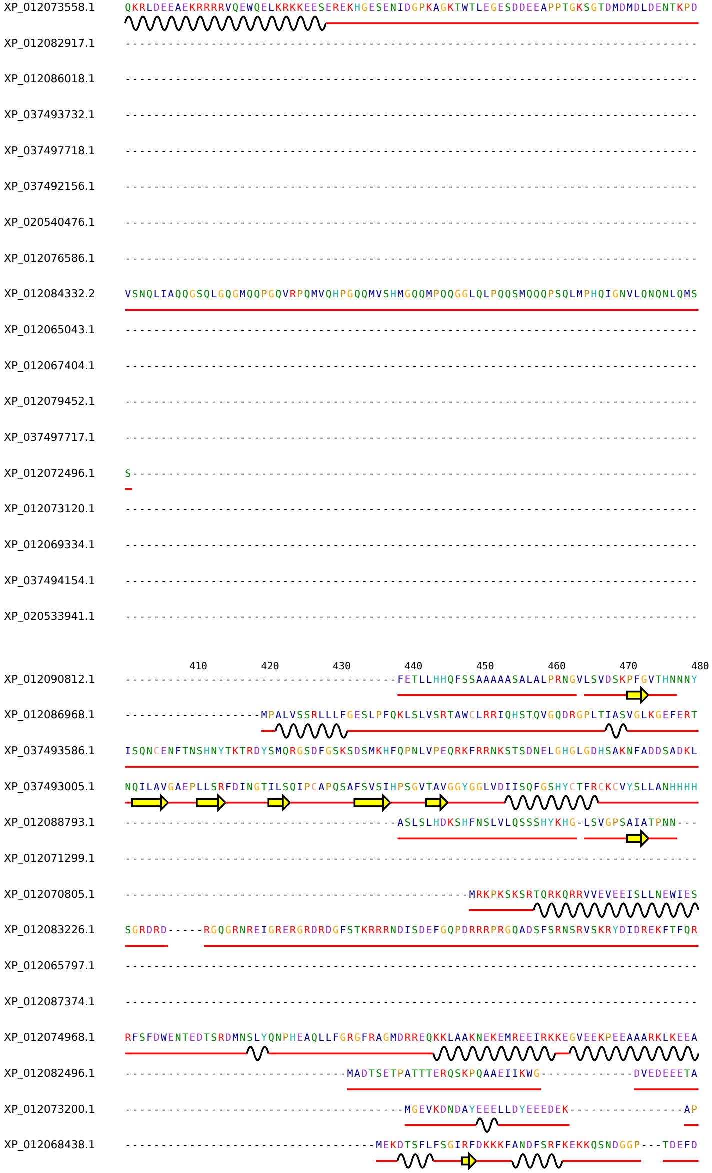

XP_012073558.1 QKRLDEEAEKRRRRVQEWQELKRKKEESEREKHGESENIDGPKAGKTWTLEGESDDEEAPPTGKSGTDMDMDLDENTKPD
XP_012082917.1 --
XP_012086018.1 --
XP_037493732.1 --
XP_037497718.1 --
XP_037492156.1 --
XP_020540476.1 --
XP_012076586.1 --
XP_012084332.2 VSNQLIAQQGSQLGQGMQQPGQVRPQMVQHPGQQMVSHMGQQMPQQGGLQLPQQSMQQQPSQLMPHQIGNVLQNQNLQMS
XP_012065043.1 --
XP_012067404.1 --
XP_012079452.1 --
XP_037497717.1 --
XP_012072496.1 S---
XP_012073120.1 --
XP_012069334.1 --
XP_037494154.1 --
XP_020533941.1 --
410 420 430 440 450 460 470 480
XP_012090812.1 --------------------------------------FETLLHHQFSSAAAAASALALPRNGVLSVDSKPFGVTHNNNY
XP_012086968.1 -------------------MPALVSSRLLLFGESLPFQKLSLVSRTAWCLRRIQHSTQVGQDRGPLTIASVGLKGEFERT
XP_037493586.1 ISQNCENFTNSHNYTKTRDYSMQRGSDFGSKSDSMKHFQPNLVPEQRKFRRNKSTSDNELGHGLGDHSAKNFADDSADKL
XP_037493005.1 NQILAVGAEPLLSRFDINGTILSQIPCAPQSAFSVSIHPSGVTAVGGYGGLVDIISQFGSHYCTFRCKCVYSLLANHHHH
XP_012088793.1 --------------------------------------ASLSLHDKSHFNSLVLQSSSHYKHG-LSVGPSAIATPNN---
XP_012071299.1 --
XP_012070805.1 --MRKPKSKSRTQRKQRRVVEVEEISLLNEWIES
XP_012083226.1 SGRDRD-----RGQGRNREIGRERGRDRDGFSTKRRRNDISDEFGQPDRRRPRGQADSFSRNSRVSKRYDIDREKFTFQR
XP_012065797.1 --
XP_012087374.1 --
XP_012074968.1 RFSFDWENTEDTSRDMNSLYQNPHEAQLLFGRGFRAGMDRREQKKLAAKNEKEMREEIRKKEGVEEKPEEAAARKLKEEA
XP_012082496.1 -------------------------------MADTSETPATTTERQSKPQAAEIIKWG-------------DVEDEEETA
XP_012073200.1 ---------------------------------------MGEVKDNDAYEEELLDYEEEDEK----------------AP
XP_012068438.1 -----------------------------------MEKDTSFLFSGIRFDKKKFANDFSRFKEKKQSNDGGP---TDEFD

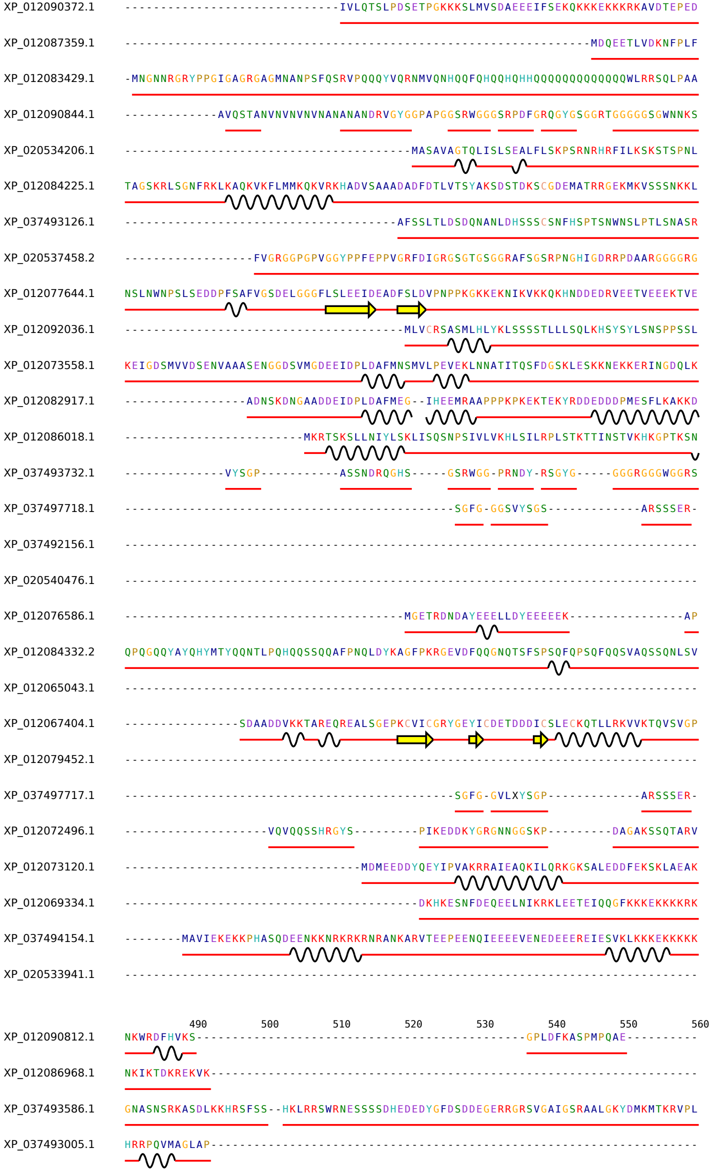

XP_012090372.1 ------------------------------IVLQTSLPDSETPGKKKSLMVSDAEEEIFSEKQKKKEKKKRKAVDTEPED
XP_012087359.1 ---MDQEETLVDKNFPLF
XP_012083429.1 -MNGNNRGRYPPGIGAGRGAGMNANPSFQSRVPQQQYVQRNMVQNHQQFQHQQHQHHQQQQQQQQQQQQQWLRRSQLPAA
XP_012090844.1 -------------AVQSTANVNVNVNVNANANANDRVGYGGPAPGGSRWGGGSRPDFGRQGYGSGGRTGGGGGSGWNNKS
XP_020534206.1 --MASAVAGTQLISLSEALFLSKPSRNRHRFILKSKSTSPNL
XP_012084225.1 TAGSKRLSGNFRKLKAQKVKFLMMKQKVRKHADVSAAADADFDTLVTSYAKSDSTDKSCGDEMATRRGEKMKVSSSNKKL
XP_037493126.1 --------------------------------------AFSSLTLDSDQNANLDHSSSCSNFHSPTSNWNSLPTLSNASR
XP_020537458.2 ------------------FVGRGGPGPVGGYPPFEPPVGRFDIGRGSGTGSGGRAFSGSRPNGHIGDRRPDAARGGGGRG
XP_012077644.1 NSLNWNPSLSEDDPFSAFVGSDELGGGFLSLEEIDEADFSLDVPNPPKGKKEKNIKVKKQKHNDDEDRVEETVEEEKTVE
XP_012092036.1 ---------------------------------------MLVCRSASMLHLYKLSSSSTLLLSQLKHSYSYLSNSPPSSL
XP_012073558.1 KEIGDSMVVDSENVAAASENGGDSVMGDEEIDPLDAFMNSMVLPEVEKLNNATITQSFDGSKLESKKNEKKERINGDQLK
XP_012082917.1 -----------------ADNSKDNGAADDEIDPLDAFMEG--IHEEMRAAPPPKPKEKTEKYRDDEDDDPMESFLKAKKD
XP_012086018.1 -------------------------MKRTSKSLLNIYLSKLISQSNPSIVLVKHLSILRPLSTKTTINSTVKHKGPTKSN
XP_037493732.1 --------------VYSGP-----------ASSNDRQGHS-----GSRWGG-PRNDY-RSGYG-----GGGRGGGWGGRS
XP_037497718.1 --SGFG-GGSVYSGS-------------ARSSSER-
XP_037492156.1 --
XP_020540476.1 --
XP_012076586.1 ---------------------------------------MGETRDNDAYEEELLDYEEEEEK----------------AP
XP_012084332.2 QPQGQQYAYQHYMTYQQNTLPQHQQSSQQAFPNQLDYKAGFPKRGEVDFQQGNQTSFSPSQFQPSQFQQSVAQSSQNLSV
XP_012065043.1 --
XP_012067404.1 ----------------SDAADDVKKTAREQREALSGEPKCVICGRYGEYICDETDDDICSLECKQTLLRKVVKTQVSVGP
XP_012079452.1 --
XP_037497717.1 --SGFG-GVLXYSGP-------------ARSSSER-
XP_012072496.1 --------------------VQVQQSSHRGYS---------PIKEDDKYGRGNNGGSKP---------DAGAKSSQTARV
XP_012073120.1 ---------------------------------MDMEEDDYQEYIPVAKRRAIEAQKILQRKGKSALEDDFEKSKLAEAK
XP_012069334.1 ---DKHKESNFDEQEELNIKRKLEETEIQQGFKKKEKKKKRK
XP_037494154.1 --------MAVIEKEKKPHASQDEENKKNRKRKRNRANKARVTEEPEENQIEEEEVENEDEEEREIESVKLKKKEKKKKK
XP_020533941.1 --
490 500 510 520 530 540 550 560
XP_012090812.1 NKWRDFHVKS--GPLDFKASPMPQAE----------
XP_012086968.1 NKIKTDKREKVK--
XP_037493586.1 GNASNSRKASDLKKHRSFSS--HKLRRSWRNESSSSDHEDEDYGFDSDDEGERRGRSVGAIGSRAALGKYDMKMTKRVPL
XP_037493005.1 HRRPQVMAGLAP--

XP_012088793.1	SILSEEAFKA--LTERFSEYDDDEND----------
XP_012071299.1	--
XP_012070805.1	QKPESGSNP---
XP_012083226.1	KKVSNTGIMNGTRGDGGVNSGFRRDVKGLREQRGFCVDEEDDRDID-DNNGDERKELMGRLQDLADE-------------
XP_012065797.1	--MAAAVTSVVP--
XP_012087374.1	--
XP_012074968.1	ADMYDTFDMRVD--
XP_012082496.1	STSEDKVASQLE--
XP_012073200.1	DS----VSAKAG--
XP_012068438.1	SGDFEPVDVAVSARKRKRKDVTSSEIVEGFSVFKSSKSVSLSIDE-------------QNNQEEKNEKS-----------
XP_012090372.1	EEKSETSSELVEP---
XP_012087359.1	SKSAKTLKRLQNP---
XP_012083429.1	DSSVDEVEKTVQ--
XP_012090844.1	SGWDRVRDRE--VNPFGNDDG-----------
XP_020534206.1	SSLFNSRDRLILS---
XP_012084225.1	SEYQTLDYEIMPDKPVKSIPRRAGTVHAG---
XP_037493126.1	RTCSTAFQSP--RTLSIPEPVFPQWNP---------
XP_020537458.2	RDNGRGGHRGGRVSGGGGRGFAGGRGFDGGRGGGRGARHGGVASRGDLDDIALPKQDFGNLVPFKKN--------FYIES
XP_012077644.1	NEKMKKKKKKKRK---
XP_012092036.1	FFPSSSLRIRFFS---
XP_012073558.1	KSSTKSLGRIIPGEDSDSEFVDLENDEDP-LDEEDDDEFMKRVKKTKAEKLSVVDHSKIDYKPFRKNFY-----------
XP_012082917.1	LGLTLAADALHAGYDSDEEVYAAAKAVDAGMLEYDSDDNPVVVDKKKIEPIPALDHSSIEYEPFNKDFY-----------
XP_012086018.1	KDSFILEKFRQRK---
XP_037493732.1	GGWG-GRDRE--VNPFGDDD------------
XP_037497718.1	RDYGADSPR---KSDLDGLTPFEKN--------FYIES
XP_037492156.1	--
XP_020540476.1	--
XP_012076586.1	DS----VAAKVN--
XP_012084332.2	GGDSASTPHTGAHLGQSQQFIGSAVNVQQPASMAQLQQTGDDVVHQQHGPRFQNHIGPSMMHNQQSNVPLQKSKNIHGGS
XP_012065043.1	--
XP_012067404.1	SAPAVRLAATDECFYVRES---
XP_012079452.1	--
XP_037497717.1	RDYGADSPR---KSDLDGLTPFEKN--------FYIES

```
XP_012072496.1   GP------------GQSQTVPSDTAN------------------------------GPGGLYAR-----------THGSS
XP_012073120.1   PSLLVKASQLKRDQPEISQTEQIVQQEKEMIEHLSDRKTLMSVRELAKG---------ITYTEPLLTGW-----------
XP_012069334.1   EEKENGGDK------------------------------------------------DEEIKLHENN-------------
XP_037494154.1   NKKVENEVKNSEE-------------------------------------------------------------------
XP_020533941.1   ------MAGLAP--------------------------------------------------------------------

                          570       580       590       600       610       620       630       640
XP_012090812.1   --------------------------------------------------------------------------------
XP_012086968.1   --------------------------------------------------------------------------------
XP_037493586.1   KELED------------------------------------------------------------------------ETN
XP_037493005.1   --------------------------------------------------------------------------------
XP_012088793.1   --------------------------------------------------------------------------------
XP_012071299.1   --------------------------------------------------------------------------------
XP_012070805.1   --------------------------------------------------------------------------------
XP_012083226.1   EESDD------------------------------------------------------------------------LDE
XP_012065797.1   --------------------------------------------------------------------------------
XP_012087374.1   --------------------------------------------------------------------------------
XP_012074968.1   --------------------------------------------------------------------------------
XP_012082496.1   --------------------------------------------------------------------------------
XP_012073200.1   --------------------------------------------------------------------------------
XP_012068438.1   --------------------------------------------------------------------------------
XP_012090372.1   --------------------------------------------------------------------------------
XP_012087359.1   --------------------------------------------------------------------------------
XP_012083429.1   --------------------------------------------------------------------------------
XP_012090844.1   -------------------------------------------------------------------------------D
XP_020534206.1   --------------------------------------------------------------------------------
XP_012084225.1   --------------------------------------------------------------------------------
XP_037493126.1   -------------------------------------------------------------------------------S
XP_020537458.2   P-------------------------------------------------------------------------------
XP_012077644.1   --------------------------------------------------------------------------------
XP_012092036.1   --------------------------------------------------------------------------------
XP_012073558.1   --------------------------------------------------------------------------------
XP_012082917.1   --------------------------------------------------------------------------------
XP_012086018.1   -------------------------------------------------------------------------------L
```

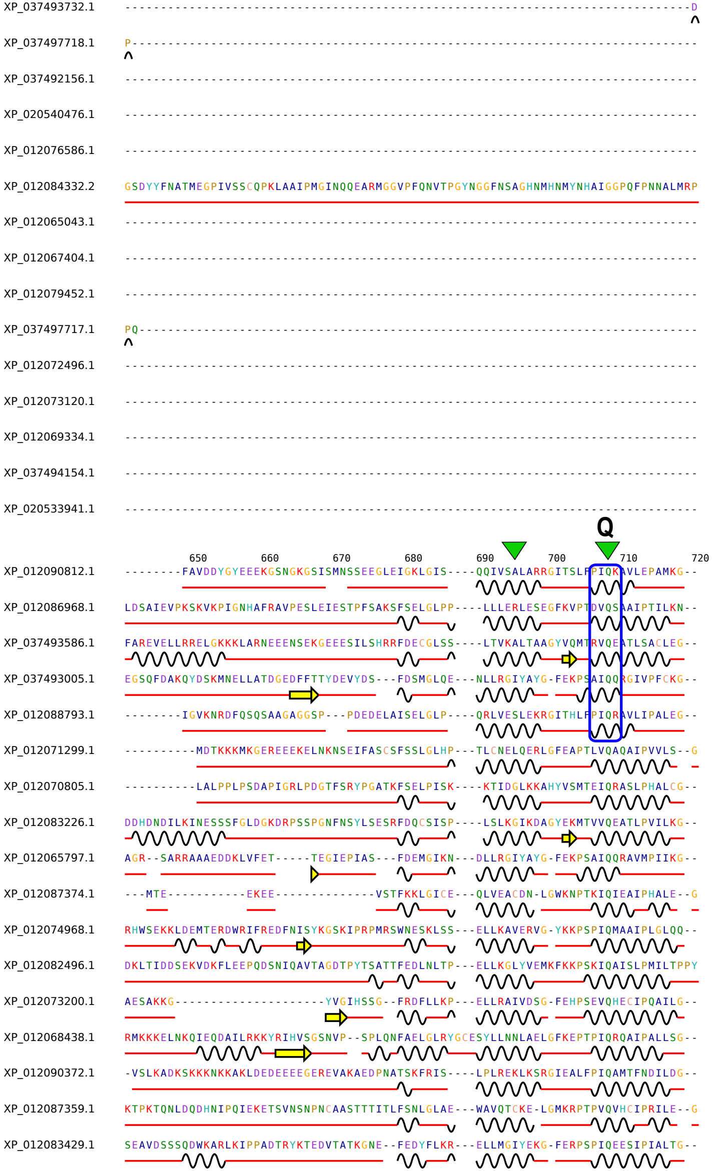
XP_037493732.1 --D
XP_037497718.1 P--
XP_037492156.1 --
XP_020540476.1 --
XP_012076586.1 --
XP_012084332.2 GSDYYFNATMEGPIVSSCQPKLAAIPMGINQQEARMGGVPFQNVTPGYNGGFNSAGHNMHNMYNHAIGGPQFPNNALMRP
XP_012065043.1 --
XP_012067404.1 --
XP_012079452.1 --
XP_037497717.1 PQ--
XP_012072496.1 --
XP_012073120.1 --
XP_012069334.1 --
XP_037494154.1 --
XP_020533941.1 --
Q
650 660 670 680 690 700 710 720
XP_012090812.1 --------FAVDDYGYEEEKGSNGKGSISMNSSEEGLEIGKLGIS----QQIVSALARRGITSLFPIQKAVLEPAMKG--
XP_012086968.1 LDSAIEVPKSKVKPIGNHAFRAVPESLEIESTPFSAKSFSELGLPP----LLLERLESEGFKVPTDVQSAAIPTILKN--
XP_037493586.1 FAREVELLRRELGKKKLARNEEENSEKGEEESILSHRRFDECGLSS----LTVKALTAAGYVQMTRVQEATLSACLEG--
XP_037493005.1 EGSQFDAKQYDSKMNELLATDGEDFFTTYDEVYDS---FDSMGLQE---NLLRGIYAYG-FEKPSAIQQRGIVPFCKG--
XP_012088793.1 --------IGVKNRDFQSQSAAGAGGSP---PDEDELAISELGLP----QRLVESLEKRGITHLFPIQRAVLIPALEG--
XP_012071299.1 ----------MDTKKKMKGEREEEKELNKNSEIFASCSFSSLGLHP---TLCNELQERLGFEAPTLVQAQAIPVVLS--G
XP_012070805.1 ----------LALPPLPSDAPIGRLPDGTFSRYPGATKFSELPISK---KTIDGLKKAHYVSMTEIQRASLPHALCG--
XP_012083226.1 DDHDNDILKINESSSFGLDGKDRPSSPGNFNSYLSESRFDQCSISP----LSLKGIKDAGYEKMTVVQEATLPVILKG--
XP_012065797.1 AGR--SARRAAAEDDKLVFET-----TEGIEPIAS---FDEMGIKN---DLLRGIYAYG-FEKPSAIQQRAVMPIIKG--
XP_012087374.1 ---MTE-----------EKEE--------------VSTFKKLGICE---QLVEACDN-LGWKNPTKIQIEAIPHALE--G
XP_012074968.1 RHWSEKKLDEMTERDWRIFREDFNISYKGSKIPRPMRSWNESKLSS---ELLKAVERVG-YKKPSPIQMAAIPLGLQQ--
XP_012082496.1 DKLTIDDSEKVDKFLEEPQDSNIQAVTAGDTPYTSATTFEDLNLTP---ELLKGLYVEMKFKKPSKIQAISLPMILTPPY
XP_012073200.1 AESAKKG-------------------YVGIHSSG--FRDFLLKP---ELLRAIVDSG-FEHPSEVQHECIPQAILG--
XP_012068438.1 RMKKKELNKQIEQDAILRKKYRIHVSGSNVP--SPLQNFAELGLRYGCESYLLNNLAELGFKEPTPIQRQAIPALLSG--
XP_012090372.1 -VSLKADKSKKKNKKAKLDEDEEEEGEREVAKAEDPNATSKFRIS----LPLREKLKSRGIEALFPIQAMTFNDILDG--
XP_012087359.1 KTPKTQNLDQDHNIPQIEKETSVNSNPNCAASTTTITLFSNLGLAE---WAVQTCKE-LGMKRPTPVQVHCIPRILE--G
XP_012083429.1 SEAVDSSSQDWKARLKIPPADTRYKTEDVTATKGNE--FEDYFLKR---ELLMGIYEKG-FERPSPIQEESIPIALTG--

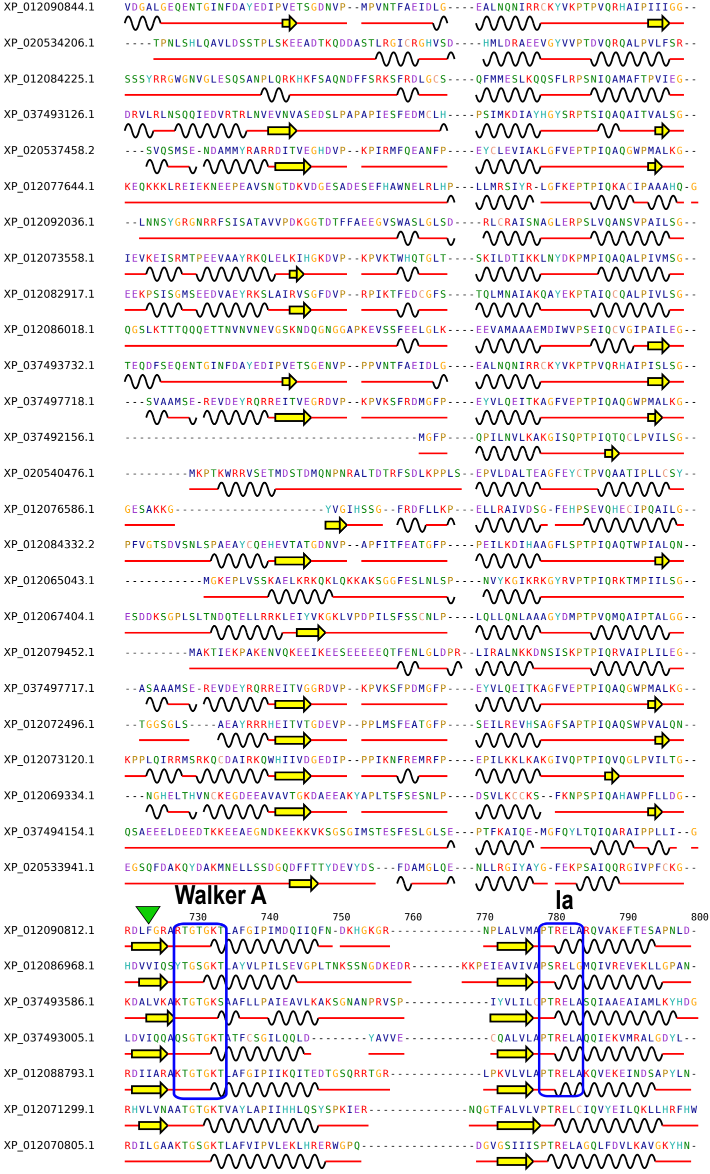
XP_012090844.1 VDGALGEQENTGINFDAYEDIPVETSGDNVP--MPVNTFAEIDLG----EALNQNIRRCKYVKPTPVQRHAIPIIIGG--
XP_020534206.1 ----TPNLSHLQAVLDSSTPLSKEEADTKQDDASTLRGICRGHVSD----HMLDRAEEVGYVVPTDVQRQALPVLFSR--
XP_012084225.1 SSSYRRGWGNVGLESQSANPLQRKHKFSAQNDFFSRKSFRDLGCS----QFMMESLKQQSFLRPSNIQAMAFTPVIEG--
XP_037493126.1 DRVLRLNSQQIEDVRTRLNVEVNVASEDSLPAPAPIESFEDMCLH----PSIMKDIAYHGYSRPTSIQAQAITVALSG--
XP_020537458.2 ---SVQSMSE-NDAMMYRARRDITVEGHDVP--KPIRMFQEANFP----EYCLEVIAKLGFVEPTPIQAQGWPMALKG--
XP_012077644.1 KEQKKKLREIEKNEEPEAVSNGTDKVDGESADESEFHAWNELRLHP---LLMRSIYR-LGFKEPTPIQKACIPAAAHQ-G
XP_012092036.1 --LNNSYGRGNRRFSISATAVVPDKGGTDTFFAEEGVSWASLGLSD----RLCRAISNAGLERPSLVQANSVPAILSG--
XP_012073558.1 IEVKEISRMTPEEVAAYRKQLELKIHGKDVP--KPVKTWHQTGLT----SKILDTIKKLNYDKPMPIQAQALPIVMSG--
XP_012082917.1 EEKPSISGMSEEDVAEYRKSLAIRVSGFDVP--RPIKTFEDCGFS----TQLMNAIAKQAYEKPTAIQCQALPIVLSG--
XP_012086018.1 QGSLKTTTQQQETTNVNVNEVGSKNDQGNGGAPKEVSSFEELGLK----EEVAMAAAEMDIWVPSEIQCVGIPAILEG--
XP_037493732.1 TEQDFSEQENTGINFDAYEDIPVETSGENVP--PPVNTFAEIDLG----EALNQNIRRCKYVKPTPVQRHAIPISLSG--
XP_037497718.1 ---SVAAMSE-REVDEYRQRREITVEGRDVP--KPVKSFRDMGFP----EYVLQEITKAGFVEPTPIQAQGWPMALKG--
XP_037492156.1 ---MGFP----QPILNVLKAKGISQPTPIQTQCLPVILSG--
XP_020540476.1 ---------MKPTKWRRVSETMDSTDMQNPNRALTDTRFSDLKPPLS--EPVLDALTEAGFEYCTPVQAATIPLLCSY--
XP_012076586.1 GESAKKG---------------------YVGIHSSG--FRDFLLKP---ELLRAIVDSG-FEHPSEVQHECIPQAILG--
XP_012084332.2 PFVGTSDVSNLSPAEAYCQEHEVTATGDNVP--APFITFEATGFP----PEILKDIHAAGFLSPTPIQAQTWPIALQN--
XP_012065043.1 -----------MGKEPLVSSKAELKRKQKLQKKAKSGGFESLNLSP---NVYKGIKRKGYRVPTPIQRKTMPIILSG--
XP_012067404.1 ESDDKSGPLSLTNDQTELLRRKLEIYVKGKLVPDPILSFSSCNLP----LQLLQNLAAAGYDMPTPVQMQAIPTALGG--
XP_012079452.1 ---------MAKTIEKPAKENVQKEEIKEESEEEEEQTFENLGLDPR--LIRALNKKDNSISKPTPIQRVAIPLILEG--
XP_037497717.1 --ASAAAMSE-REVDEYRQRREITVGGRDVP--KPVKSFPDMGFP----EYVLQEITKAGFVEPTPIQAQGWPMALKG--
XP_012072496.1 --TGGSGLS----AEAYRRRHEITVTGDEVP--PPLMSFEATGFP----SEILREVHSAGFSAPTPIQAQSWPVALQN--
XP_012073120.1 KPPLQIRRMSRKQCDAIRKQWHIIVDGEDIP--PPIKNFREMRFP----EPILKKLKAKGIVQPTPIQVQGLPVILTG--
XP_012069334.1 ---NGHELTHVNCKEGDEEAVAVTGKDAEEAKYAPLTSFSESNLP----DSVLKCCKS--FKNPSPIQAHAWPFLLDG--
XP_037494154.1 QSAEEELDEEDTKKEEAEGNDKEEKKVKSGSGIMSTESFESLGLSE---PTFKAIQE-MGFQYLTQIQARAIPPLLI--G
XP_020533941.1 EGSQFDAKQYDAKMNELLSSDGQDFFTTYDEVYDS---FDAMGLQE---NLLRGIYAYG-FEKPSAIQQRGIVPFCKG--
Walker A
Ia
730 740 750 760 770 780 790 800
XP_012090812.1 RDLFGRARTGTGKTLAFGIPIMDQIIQFN-DKHGKGR------------NPLALVMAPTRELARQVAKEFTESAPNLD-
XP_012086968.1 HDVVIQSYTGSGKTLAYVLPILSEVGPLTNKSSNGDKEDR------KKPEIEAVIVAPSRELGMQIVREVEKLLGPAN-
XP_037493586.1 KDALVKAKTGTGKSAAFLLPAIEAVLKAKSGNANPRVSP-----------IYVLILCPTRELASQIAAEAIAMLKYHDG
XP_037493005.1 LDVIQQAQSGTGKTATFCSGILQQLD--------YAVVE-----------CQALVLAPTRELAQQIEKVMRALGDYL--
XP_012088793.1 RDIIARAKTGTGKTLAFGIPIIKQITEDTGSQRRTGR-------------LPKVLVLAPTRELAKQVEKEINDSAPYLN-
XP_012071299.1 RHVLVNAATGTGKTVAYLAPIIHHLQSYSPKIER-------------NQGTFALVLVPTRELCIQVYEILQKLLHRFHW
XP_012070805.1 RDILGAAKTGSGKTLAFVIPVLEKLHRERWGPQ----------------DGVGSIIISPTRELAGQLFDVLKAVGKYHN-

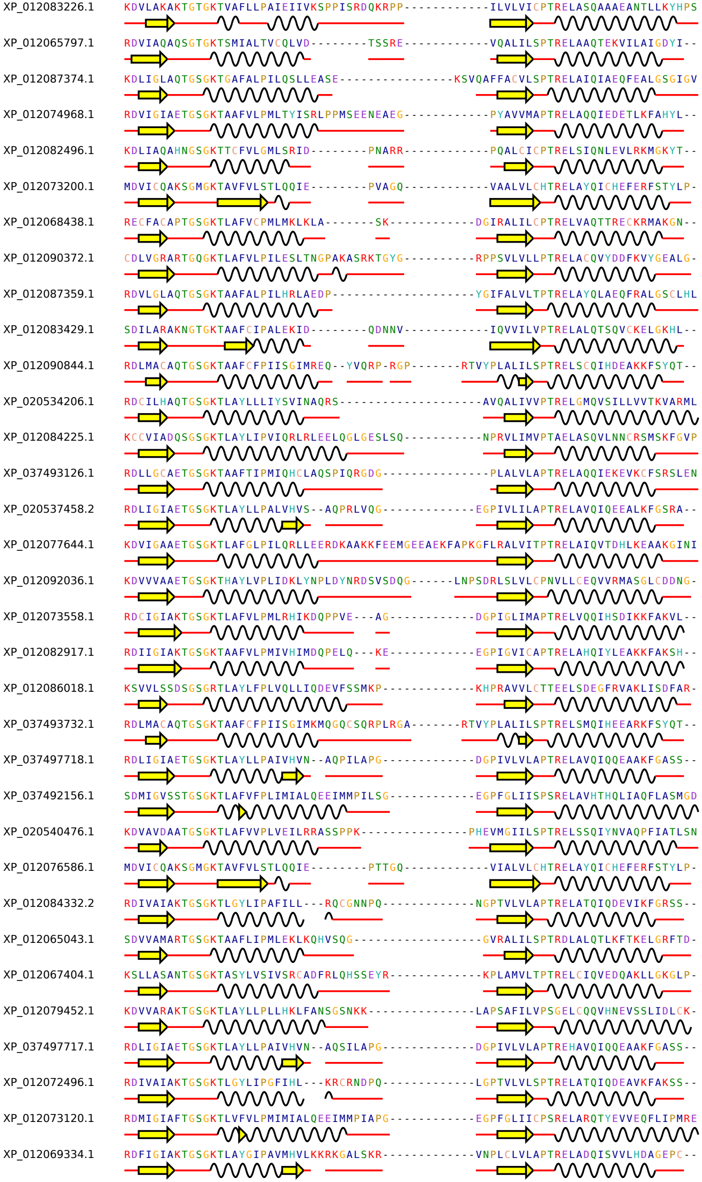

XP_012083226.1 KDVLAKAKTGTGKTVAFLLPAIEIIVKSPPISRDQKRPP-----------ILVLVICPTRELASQAAAEANTLLKYHPS
XP_012065797.1 RDVIAQAQSGTGKTSMIALTVCQLVD--------TSSRE------------VQALILSPTRELAAQTEKVILAIGDYI--
XP_012087374.1 KDLIGLAQTGSGKTGAFALPILQSLLEASE---------------KSVQAFFACVLSPTRELAIQIAEQFEALGSGIGV
XP_012074968.1 RDVIGIAETGSGKTAAFVLPMLTYISRLPPMSEENEAEG-----------PYAVVMAPTRELAQQIEDETLKFAHYL--
XP_012082496.1 KDLIAQAHNGSGKTTCFVLGMLSRID--------PNARR------------PQALCICPTRELSIQNLEVLRKMGKYT--
XP_012073200.1 MDVICQAKSGMGKTAVFVLSTLQQIE--------PVAGQ-----------VAALVLCHTRELAYQICHEFERFSTYLP-
XP_012068438.1 RECFACAPTGSGKTLAFVCPMLMKLKLA-------SK-----------DGIRALILCPTRELVAQTTRECKRMAKGN--
XP_012090372.1 CDLVGRARTGQGKTLAFVLPILESLTNGPAKASRKTGYG----------RPPSVLVLLPTRELACQVYDDFKVYGEALG-
XP_012087359.1 RDVLGLAQTGSGKTAAFALPILHRLAEDP------------------YGIFALVLTPTRELAYQLAEQFRALGSCLHL
XP_012083429.1 SDILARAKNGTGKTAAFCIPALEKID--------QDNNV-----------IQVVILVPTRELALQTSQVCKELGKHL--
XP_012090844.1 RDLMACAQTGSGKTAAFCFPIISGIMREQ--YVQRP-RGP-------RTVYPLALILSPTRELSCQIHDEAKKFSYQT--
XP_020534206.1 RDCILHAQTGSGKTLAYLLLIYSVINAQRS-----------------AVQALIVVPTRELGMQVSILLVVTKVARML
XP_012084225.1 KCCVIADQSGSGKTLAYLIPVIQRLRLEELQGLGESLSQ----------NPRVLIMVPTAELASQVLNNCRSMSKFGVP
XP_037493126.1 RDLLGCAETGSGKTAAFTIPMIQHCLAQSPIQRGDG--------------PLALVLAPTRELAQQIEKEVKCFSRSLEN
XP_020537458.2 RDLIGIAETGSGKTLAYLLPALVHVS--AQPRLVQG------------EGPIVLILAPTRELAVQIQEEALKFGSRA--
XP_012077644.1 KDVIGAAETGSGKTLAFGLPILQRLLEERDKAAKKFEEMGEEAEKFAPKGFLRALVITPTRELAIQVTDHLKEAAKGINI
XP_012092036.1 KDVVVAAETGSGKTHAYLVPLIDKLYNPLDYNRDSVSDQG------LNPSDRLSLVLCPNVLLCEQVVRMASGLCDDNG-
XP_012073558.1 RDCIGIAKTGSGKTLAFVLPMLRHIKDQPPVE---AG-----------DGPIGLIMAPTRELVQQIHSDIKKFAKVL--
XP_012082917.1 RDIIGIAKTGSGKTAAFVLPMIVHIMDQPELQ---KE-----------EGPIGVICAPTRELAHQIYLEAKKFAKSH--
XP_012086018.1 KSVVLSSDSGSGRTLAYLFPLVQLLIQDEVFSSMKP------------KHPRAVVLCTTEELSDEGFRVAKLISDFAR-
XP_037493732.1 RDLMACAQTGSGKTAAFCFPIISGIMKMQGQCSQRPLRGA-------RTVYPLALILSPTRELSMQIHEEARKFSYQT--
XP_037497718.1 RDLIGIAETGSGKTLAYLLPAIVHVN--AQPILAPG------------DGPIVLVLAPTRELAVQIQQEAAKFGASS--
XP_037492156.1 SDMIGVSSTGSGKTLAFVFPLIMIALQEEIMMPILSG-----------EGPFGLIISPSRELAVHTHQLIAQFLASMGD
XP_020540476.1 KDVAVDAATGSGKTLAFVVPLVEILRRASSPPK--------------PHEVMGIILSPTRELSSQIYNVAQPFIATLSN
XP_012076586.1 MDVICQAKSGMGKTAVFVLSTLQQIE--------PTTGQ-----------VIALVLCHTRELAYQICHEFERFSTYLP-
XP_012084332.2 RDIVAIAKTGSGKTLGYLIPAFILL---RQCGNNPQ-------------NGPTVLVLAPTRELATQIQDEVIKFGRSS--
XP_012065043.1 SDVVAMARTGSGKTAAFLIPMLEKLKQHVSQG-----------------GVRALILSPTRDLALQTLKFTKELGRFTD-
XP_012067404.1 KSLLASANTGSGKTASYLVSIVSRCADFRLQHSSEYR------------KPLAMVLTPTRELCIQVEDQAKLLGKGLP-
XP_012079452.1 KDVVARAKTGSGKTLAYLLPLLHKLFANSGSNKK--------------LAPSAFILVPSGELCQQVHNEVSSLIDLCK-
XP_037497717.1 RDLIGIAETGSGKTLAYLLPAIVHVN--AQSILAPG------------DGPIVLVLAPTREHAVQIQQEAAKFGASS--
XP_012072496.1 RDIVAIAKTGSGKTLGYLIPGFIHL---KRCRNDPQ-------------LGPTVLVLSPTRELATQIQDEAVKFAKSS--
XP_012073120.1 RDMIGIAFTGSGKTLVFVLPMIMIALQEEIMMPIAPG-----------EGPFGLIICPSRELARQTYEVVEQFLIPMRE
XP_012069334.1 RDFIGIAKTGSGKTLAYGIPAVMHVLKKRKGALSKR------------VNPLCLVLAPTRELADQISVVLHDAGEPC--

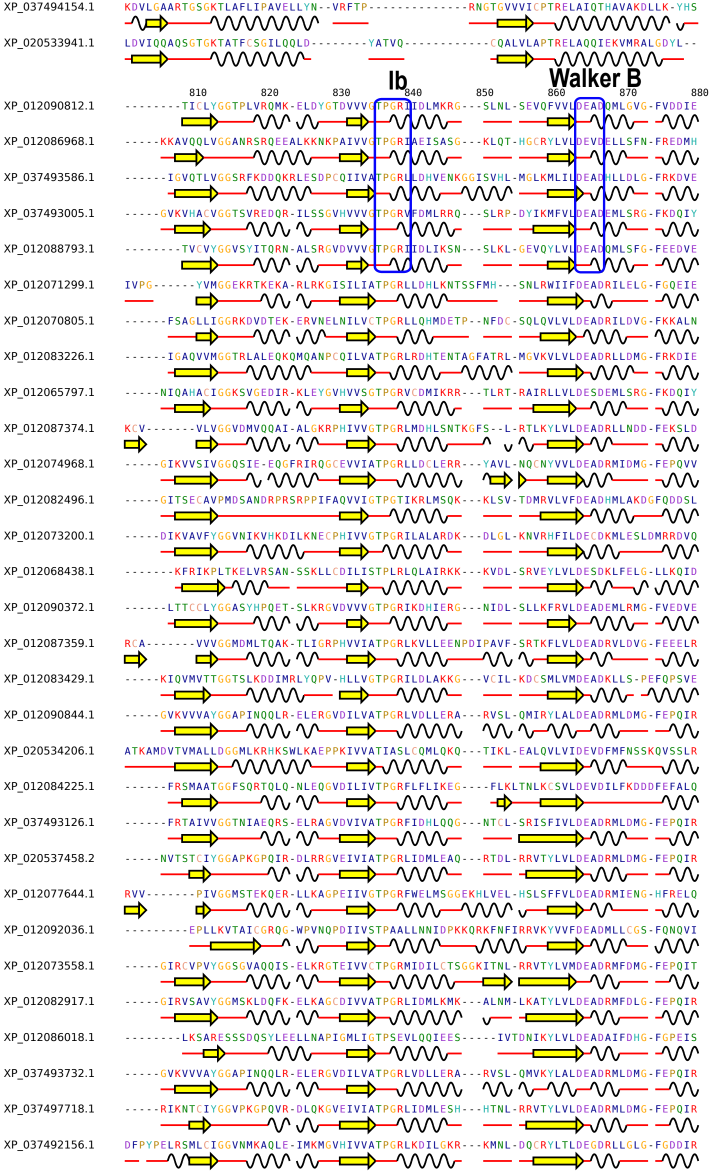

XP_037494154.1 KDVLGAARTGSGKTLAFLIPAVELLYN--VRFTP-------------RNGTGVVVICPTRELAIQTHAVAKDLLK-YHS
XP_020533941.1 LDVIQQAQSGTGKTATFCSGILQQLD--------YATVQ-----------CQALVLAPTRELAQQIEKVMRALGDYL--
Ib
Walker B
810 820 830 840 850 860 870 880
XP_012090812.1 --------TICLYGGTPLVRQMK-ELDYGTDVVVGTPGRIIDLMKRG---SLNL-SEVQFVVLDEADQMLGVG-FVDDIE
XP_012086968.1 -----KKAVQQLVGGANRSRQEEALKKNKPAIVVGTPGRIAEISASG---KLQT-HGCRYLVLDEVDELLSFN-FREDMH
XP_037493586.1 ------IGVQTLVGGSRFKDDQKRLESDPCQIIVATPGRLLDHVENKGGISVHL-MGLKMLILDEADHLLDLG-FRKDVE
XP_037493005.1 -----GVKVHACVGGTSVREDQR-ILSSGVHVVVGTPGRVFDMLRRQ---SLRP-DYIKMFVLDEADEMLSRG-FKDQIY
XP_012088793.1 --------TVCVYGGVSYITQRN-ALSRGVDVVVGTPGRIIDLIKSN---SLKL-GEVQYLVLDEADQMLSFG-FEEDVE
XP_012071299.1 IVPG------YVMGGEKRTKEKA-RLRKGISILIATPGRLLDHLKNTSSFMH---SNLRWIIFDEADRILELG-FGQEIE
XP_012070805.1 ------FSAGLLIGGRKDVDTEK-ERVNELNILVCTPGRLLQHMDETP--NFDC-SQLQVLVLDEADRILDVG-FKKALN
XP_012083226.1 ------IGAQVVMGGTRLALEQKQMQANPCQILVATPGRLRDHTENTAGFATRL-MGVKVLVLDEADRLLDMG-FRKDIE
XP_012065797.1 -----NIQAHACIGGKSVGEDIR-KLEYGVHVVSGTPGRVCDMIKRR---TLRT-RAIRLLVLDESDEMLSRG-FKDQIY
XP_012087374.1 KCV-------VLVGGVDMVQQAI-ALGKRPHIVVGTPGRLMDHLSNTKGFS--L-RTLKYLVLDEADRLLNDD-FEKSLD
XP_012074968.1 -----GIKVVSIVGGQSIE-EQGFRIRQGCEVVIATPGRLLDCLERR---YAVL-NQCNYVVLDEADRMIDMG-FEPQVV
XP_012082496.1 -----GITSECAVPMDSANDRPRSRPPIFAQVVIGTPGTIKRLMSQK---KLSV-TDMRVLVFDEADHMLAKDGFQDDSL
XP_012073200.1 -----DIKVAVFYGGVNIKVHKDILKNECPHIVVGTPGRILALARDK---DLGL-KNVRHFILDECDKMLESLDMRRDVQ
XP_012068438.1 -------KFRIKPLTKELVRSAN-SSKLLCDILISTPLRLQLAIRKK---KVDL-SRVEYLVLDESDKLFELG-LLKQID
XP_012090372.1 ------LTTCCLYGGASYHPQET-SLKRGVDVVVGTPGRIKDHIERG---NIDL-SLLKFRVLDEADEMLRMG-FVEDVE
XP_012087359.1 RCA-------VVVGGMDMLTQAK-TLIGRPHVVIATPGRLKVLLEENPDIPAVF-SRTKFLVLDEADRVLDVG-FEEELR
XP_012083429.1 -----KIQVMVTTGGTSLKDDIMRLYQPV-HLLVGTPGRILDLAKKG---VCIL-KDCSMLVMDEADKLLS-PEFQPSVE
XP_012090844.1 -----GVKVVVAYGGAPINQQLR-ELERGVDILVATPGRLVDLLERA---RVSL-QMIRYLALDEADRMLDMG-FEPQIR
XP_020534206.1 ATKAMDVTVMALLDGGMLKRHKSWLKAEPPKIVVATIASLCQMLQKQ---TIKL-EALQVLVIDEVDFMFNSSKQVSSLR
XP_012084225.1 ------FRSMAATGGFSQRTQLQ-NLEQGVDILIVTPGRFLFLIKEG----FLKLTNLKCSVLDEVDILFKDDDFEFALQ
XP_037493126.1 ------FRTAIVVGGTNIAEQRS-ELRAGVDVIVATPGRFIDHLQQG---NTCL-SRISFIVLDEADRMLDMG-FEPQIR
XP_020537458.2 -----NVTSTCIYGGAPKGPQIR-DLRRGVEIVIATPGRLIDMLEAQ---RTDL-RRVTYLVLDEADRMLDMG-FEPQIR
XP_012077644.1 RVV-------PIVGGMSTEKQER-LLKAGPEIIVGTPGRFWELMSGGEKHLVEL-HSLSFFVLDEADRMIENG-HFRELQ
XP_012092036.1 ---------EPLLKVTAICGRQG-WPVNQPDIIVSTPAALLNNIDPKKQRKFNFIRRVKYVVFDEADMLLCGS-FQNQVI
XP_012073558.1 -----GIRCVPVYGGSGVAQQIS-ELKRGTEIVVCTPGRMIDILCTSGGKITNL-RRVTYLVMDEADRMFDMG-FEPQIT
XP_012082917.1 -----GIRVSAVYGGMSKLDQFK-ELKAGCDIVVATPGRLIDMLKMK---ALNM-LKATYLVLDEADRMFDLG-FEPQIR
XP_012086018.1 --------LKSARESSSDQSYLEELLNAPIGMLIGTPSEVLQQIEES-----IVTDNIKYLVLDEADAIFDHG-FGPEIS
XP_037493732.1 -----GVKVVVAYGGAPINQQLR-ELERGVDILVATPGRLVDLLERA---RVSL-QMVKYLALDEADRMLDMG-FEPQIR
XP_037497718.1 -----RIKNTCIYGGVPKGPQVR-DLQKGVEIVIATPGRLIDMLESH---HTNL-RRVTYLVLDEADRMLDMG-FEPQIR
XP_037492156.1 DFPYPELRSMLCIGGVNMKAQLE-IMKMGVHIVVATPGRLKDILGKR---KMNL-DQCRYLTLDEGDRLLGLG-FGDDIR

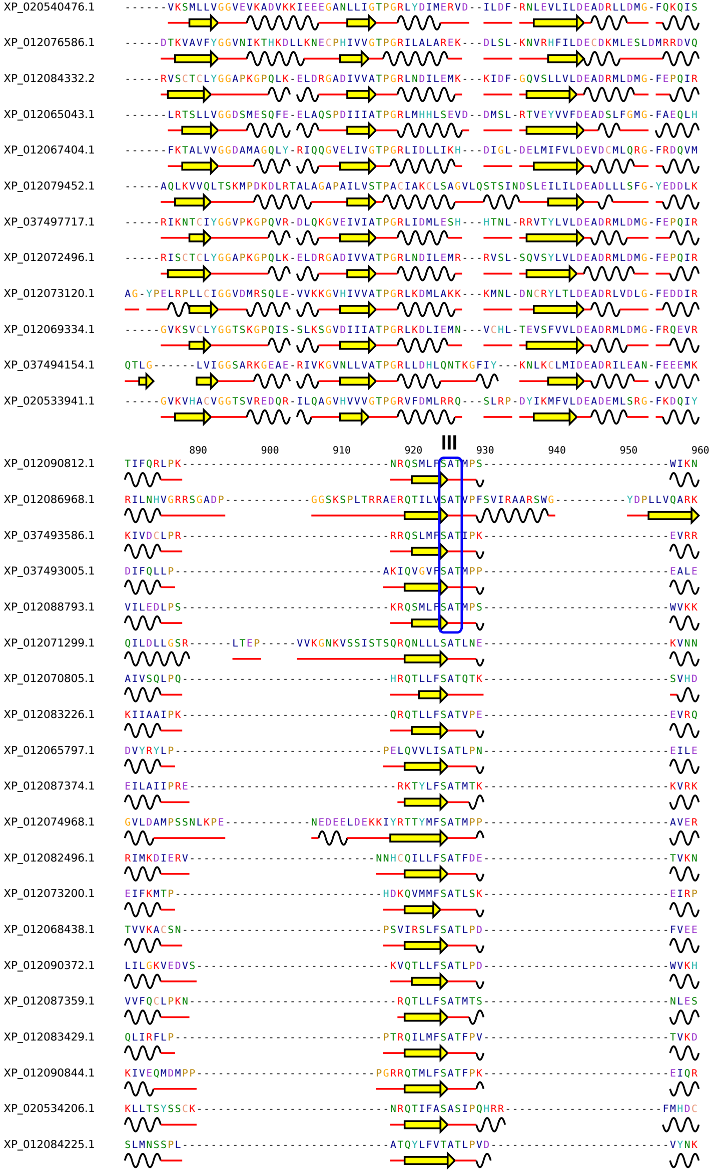
XP_020540476.1 ------VKSMLLVGGVEVKADVKKIEEEGANLLIGTPGRLYDIMERVD--ILDF-RNLEVLILDEADRLLDMG-FQKQIS
XP_012076586.1 -----DTKVAVFYGGVNIKTHKDLLKNECPHIVVGTPGRILALAREK---DLSL-KNVRHFILDECDKMLESLDMRRDVQ
XP_012084332.2 -----RVSCTCLYGGAPKGPQLK-ELDRGADIVVATPGRLNDILEMK---KIDF-GQVSLLVLDEADRMLDMG-FEPQIR
XP_012065043.1 ------LRTSLLVGGDSMESQFE-ELAQSPDIIIATPGRLMHHLSEVD--DMSL-RTVEYVVFDEADSLFGMG-FAEQLH
XP_012067404.1 ------FKTALVVGGDAMAGQLY-RIQQGVELIVGTPGRLIDLLIKH---DIGL-DELMIFVLDEVDCMLQRG-FRDQVM
XP_012079452.1 -----AQLKVVQLTSKMPDKDLRTALAGAPAILVSTPACIAKCLSAGVLQSTSINDSLEILILDEADLLLSFG-YEDDLK
XP_037497717.1 -----RIKNTCIYGGVPKGPQVR-DLQKGVEIVIATPGRLIDMLESH---HTNL-RRVTYLVLDEADRMLDMG-FEPQIR
XP_012072496.1 -----RISCTCLYGGAPKGPQLK-ELDRGADIVVATPGRLNDILEMR---RVSL-SQVSYLVLDEADRMLDMG-FEPQIR
XP_012073120.1 AG-YPELRPLLCIGGVDMRSQLE-VVKKGVHIVVATPGRLKDMLAKK---KMNL-DNCRYLTLDEADRLVDLG-FEDDIR
XP_012069334.1 -----GVKSVCLYGGTSKGPQIS-SLKSGVDIIIATPGRLKDLIEMN---VCHL-TEVSFVVLDEADRMLDMG-FRQEVR
XP_037494154.1 QTLG------LVIGGSARKGEAE-RIVKGVNLLVATPGRLLDHLQNTKGFIY---KNLKCLMIDEADRILEAN-FEEEMK
XP_020533941.1 -----GVKVHACVGGTSVREDQR-ILQAGVHVVVGTPGRVFDMLRRQ---SLRP-DYIKMFVLDEADEMLSRG-FKDQIY
III
890 900 910 920 930 940 950 960
XP_012090812.1 TIFQRLPK-----------------------------NRQSMLFSATMPS--------------------------WIKN
XP_012086968.1 RILNHVGRRSGADP------------GGSKSPLTRRAERQTILVSATVPFSVIRAARSWG----------YDPLLVQARK
XP_037493586.1 KIVDCLPR-----------------------------RRQSLMFSATIPK--------------------------EVRR
XP_037493005.1 DIFQLLP-----------------------------AKIQVGVFSATMPP--------------------------EALE
XP_012088793.1 VILEDLPS-----------------------------KRQSMLFSATMPS--------------------------WVKK
XP_012071299.1 QILDLLGSR------LTEP-----VVKGNKVSSISTSQRQNLLLSATLNE--------------------------KVNN
XP_012070805.1 AIVSQLPQ-----------------------------HRQTLLFSATQTK--------------------------SVHD
XP_012083226.1 KIIAAIPK-----------------------------QRQTLLFSATVPE--------------------------EVRQ
XP_012065797.1 DVYRYLP-----------------------------PELQVVLISATLPN--------------------------EILE
XP_012087374.1 EILAIIPRE-----------------------------RKTYLFSATMTK--------------------------KVRK
XP_012074968.1 GVLDAMPSSNLKPE------------NEDEELDEKKIYRTTYMFSATMPP--------------------------AVER
XP_012082496.1 RIMKDIERV--------------------------NNHCQILLFSATFDE--------------------------TVKN
XP_012073200.1 EIFKMTP-----------------------------HDKQVMMFSATLSK--------------------------EIRP
XP_012068438.1 TVVKACSN----------------------------PSVIRSLFSATLPD--------------------------FVEE
XP_012090372.1 LILGKVEDVS---------------------------KVQTLLFSATLPD--------------------------WVKH
XP_012087359.1 VVFQCLPKN-----------------------------RQTLLFSATMTS--------------------------NLES
XP_012083429.1 QLIRFLP-----------------------------PTRQILMFSATFPV--------------------------TVKD
XP_012090844.1 KIVEQMDMPP-------------------------PGRRQTMLFSATFPK--------------------------EIQR
XP_020534206.1 KLLTSYSSCK---------------------------NRQTIFASASIPQHRR----------------------FMHDC
XP_012084225.1 SLMNSSPL-----------------------------ATQYLFVTATLPVD-------------------------VYNK

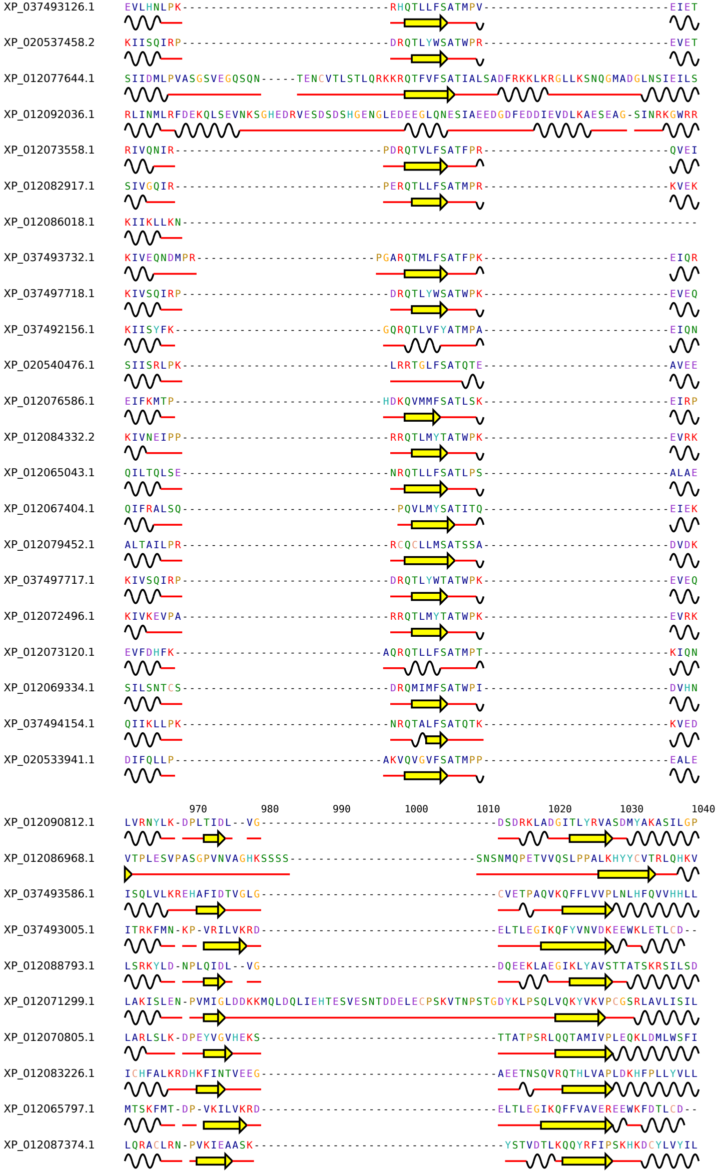
XP_037493126.1 EVLHNLPK-----------------------------RHQTLLFSATMPV--------------------------EIET
XP_020537458.2 KIISQIRP-----------------------------DRQTLYWSATWPR--------------------------EVET
XP_012077644.1 SIIDMLPVASGSVEGQSQN-----TENCVTLSTLQRKKRQTFVFSATIALSADFRKKLKRGLLKSNQGMADGLNSIEILS
XP_012092036.1 RLINMLRFDEKQLSEVNKSGHEDRVESDSDSHGENGLEDEEGLQNESIAEEDGDFEDDIEVDLKAESEAG-SINRKGWRR
XP_012073558.1 RIVQNIR-----------------------------PDRQTVLFSATFPR--------------------------QVEI
XP_012082917.1 SIVGQIR-----------------------------PERQTLLFSATMPR--------------------------KVEK
XP_012086018.1 KIIKLLKN--
XP_037493732.1 KIVEQNDMPR-------------------------PGARQTMLFSATFPK--------------------------EIQR
XP_037497718.1 KIVSQIRP-----------------------------DRQTLYWSATWPK--------------------------EVEQ
XP_037492156.1 KIISYFK-----------------------------GQRQTLVFYATMPA--------------------------EIQN
XP_020540476.1 SIISRLPK-----------------------------LRRTGLFSATQTE--------------------------AVEE
XP_012076586.1 EIFKMTP-----------------------------HDKQVMMFSATLSK--------------------------EIRP
XP_012084332.2 KIVNEIPP-----------------------------RRQTLMYTATWPK--------------------------EVRK
XP_012065043.1 QILTQLSE-----------------------------NRQTLLFSATLPS--------------------------ALAE
XP_012067404.1 QIFRALSQ------------------------------PQVLMYSATITQ--------------------------EIEK
XP_012079452.1 ALTAILPR-----------------------------RCQCLLMSATSSA--------------------------DVDK
XP_037497717.1 KIVSQIRP-----------------------------DRQTLYWTATWPK--------------------------EVEQ
XP_012072496.1 KIVKEVPA-----------------------------RRQTLMYTATWPK--------------------------EVRK
XP_012073120.1 EVFDHFK-----------------------------AQRQTLLFSATMPT--------------------------KIQN
XP_012069334.1 SILSNTCS-----------------------------DRQMIMFSATWPI--------------------------DVHN
XP_037494154.1 QIIKLLPK-----------------------------NRQTALFSATQTK--------------------------KVED
XP_020533941.1 DIFQLLP-----------------------------AKVQVGVFSATMPP--------------------------EALE
970 980 990 1000 1010 1020 1030 1040
XP_012090812.1 LVRNYLK-DPLTIDL--VG---------------------------------DSDRKLADGITLYRVASDMYAKASILGP
XP_012086968.1 VTPLESVPASGPVNVAGHKSSSS--------------------------SNSNMQPETVVQSLPPALKHYYCVTRLQHKV
XP_037493586.1 ISQLVLKREHAFIDTVGLG---------------------------------CVETPAQVKQFFLVVPLNLHFQVVHHLL
XP_037493005.1 ITRKFMN-KP-VRILVKRD---------------------------------ELTLEGIKQFYVNVDKEEWKLETLCD--
XP_012088793.1 LSRKYLD-NPLQIDL--VG---------------------------------DQEEKLAEGIKLYAVSTTATSKRSILSD
XP_012071299.1 LAKISLEN-PVMIGLDDKKMQLDQLIEHTESVESNTDDELECPSKVTNPSTGDYKLPSQLVQKYVKVPCGSRLAVLISIL
XP_012070805.1 LARLSLK-DPEYVGVHEKS---------------------------------TTATPSRLQQTAMIVPLEQKLDMLWSFI
XP_012083226.1 ICHFALKRDHKFINTVEEG---------------------------------AEETNSQVRQTHLVAPLDKHFPLLYVLL
XP_012065797.1 MTSKFMT-DP-VKILVKRD---------------------------------ELTLEGIKQFFVAVEREEWKFDTLCD--
XP_012087374.1 LQRACLRN-PVKIEAASK-----------------------------------YSTVDTLKQQYRFIPSKHKDCYLVYIL

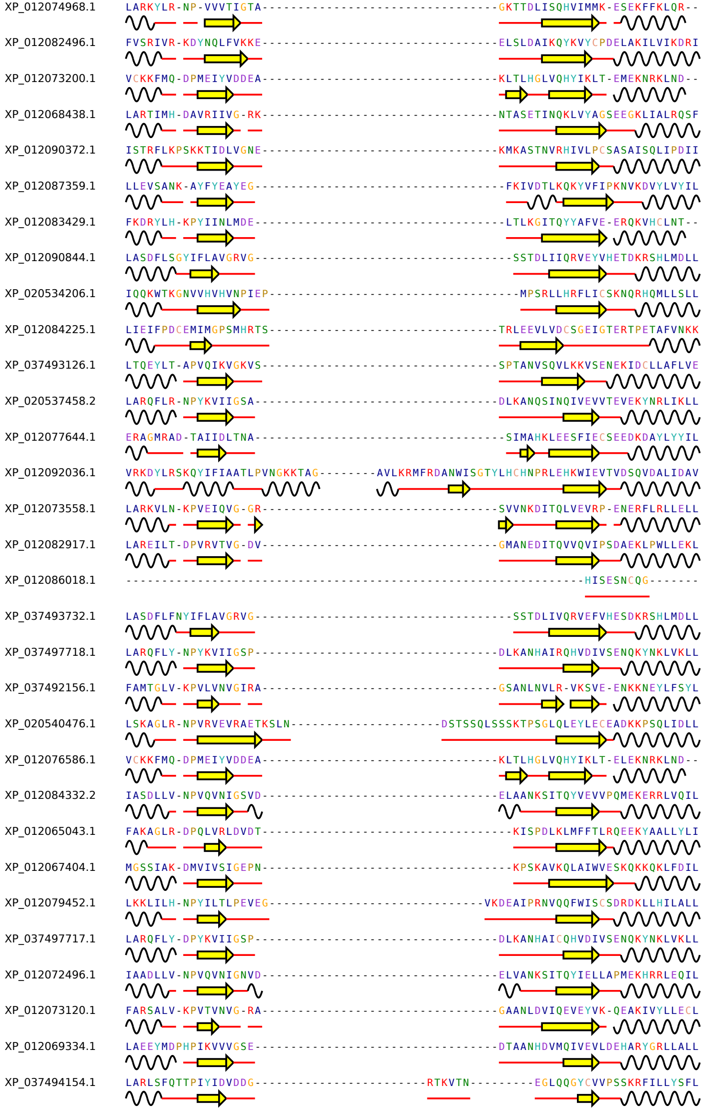

XP_012074968.1 LARKYLR-NP-VVVTIGTA---------------------------------GKTTDLISQHVIMMK-ESEKFFKLQR--
XP_012082496.1 FVSRIVR-KDYNQLFVKKE---------------------------------ELSLDAIKQYKVYCPDELAKILVIKDRI
XP_012073200.1 VCKKFMQ-DPMEIYVDDEA---------------------------------KLTLHGLVQHYIKLT-EMEKNRKLND--
XP_012068438.1 LARTIMH-DAVRIIVG-RK---------------------------------NTASETINQKLVYAGSEEGKLIALRQSF
XP_012090372.1 ISTRFLKPSKKTIDLVGNE---------------------------------KMKASTNVRHIVLPCSASAISQLIPDII
XP_012087359.1 LLEVSANK-AYFYEAYEG-----------------------------------FKIVDTLKQKYVFIPKNVKDVYLVYIL
XP_012083429.1 FKDRYLH-KPYIINLMDE-----------------------------------LTLKGITQYYAFVE-ERQKVHCLNT--
XP_012090844.1 LASDFLSGYIFLAVGRVG------------------------------------SSTDLIIQRVEYVHETDKRSHLMDLL
XP_020534206.1 IQQKWTKGNVVHVHVNPIEP-----------------------------------MPSRLLHRFLICSKNQRHQMLLSLL
XP_012084225.1 LIEIFPDCEMIMGPSMHRTS--------------------------------TRLEEVLVDCSGEIGTERTPETAFVNKK
XP_037493126.1 LTQEYLT-APVQIKVGKVS---------------------------------SPTANVSQVLKKVSENEKIDCLLAFLVE
XP_020537458.2 LARQFLR-NPYKVIIGSA----------------------------------DLKANQSINQIVEVVTEVEKYNRLIKLL
XP_012077644.1 ERAGMRAD-TAIIDLTNA-----------------------------------SIMAHKLEESFIECSEEDKDAYLYYIL
XP_012092036.1 VRKDYLRSKQYIFIAATLPVNGKKTAG--------AVLKRMFRDANWISGTYLHCHNPRLEHKWIEVTVDSQVDALIDAV
XP_012073558.1 LARKVLN-KPVEIQVG-GR---------------------------------SVVNKDITQLVEVRP-ENERFLRLLELL
XP_012082917.1 LAREILT-DPVRVTVG-DV---------------------------------GMANEDITQVVQVIPSDAEKLPWLLEKL
XP_012086018.1 --HISESNCQG-------
XP_037493732.1 LASDFLFNYIFLAVGRVG------------------------------------SSTDLIVQRVEFVHESDKRSHLMDLL
XP_037497718.1 LARQFLY-NPYKVIIGSP----------------------------------DLKANHAIRQHVDIVSENQKYNKLVKLL
XP_037492156.1 FAMTGLV-KPVLVNVGIRA---------------------------------GSANLNVLR-VKSVE-ENKKNEYLFSYL
XP_020540476.1 LSKAGLR-NPVRVEVRAETKSLN---------------------DSTSSQLSSSKTPSGLQLEYLECEADKKPSQLIDLL
XP_012076586.1 VCKKFMQ-DPMEIYVDDEA---------------------------------KLTLHGLVQHYIKLT-ELEKNRKLND--
XP_012084332.2 IASDLLV-NPVQVNIGSVD---------------------------------ELAANKSITQYVEVVPQMEKERRLVQIL
XP_012065043.1 FAKAGLR-DPQLVRLDVDT-----------------------------------KISPDLKLMFFTLRQEEKYAALLYLI
XP_012067404.1 MGSSIAK-DMVIVSIGEPN-----------------------------------KPSKAVKQLAIWVESKQKKQKLFDIL
XP_012079452.1 LKKLILH-NPYILTLPEVEG------------------------------VKDEAIPRNVQQFWISCSDRDKLLHILALL
XP_037497717.1 LARQFLY-DPYKVIIGSP----------------------------------DLKANHAICQHVDIVSENQKYNKLVKLL
XP_012072496.1 IAADLLV-NPVQVNIGNVD---------------------------------ELVANKSITQYIELLAPMEKHRRLEQIL
XP_012073120.1 FARSALV-KPVTVNVG-RA---------------------------------GAANLDVIQEVEYVK-QEAKIVYLLECL
XP_012069334.1 LAEEYMDPHPIKVVVGSE----------------------------------DTAANHDVMQIVEVLDEHARYGRLLALL
XP_037494154.1 LARLSFQTTPIYIDVDDG------------------------RTKVTN---------EGLQQGYCVVPSSKRFILLYSFL

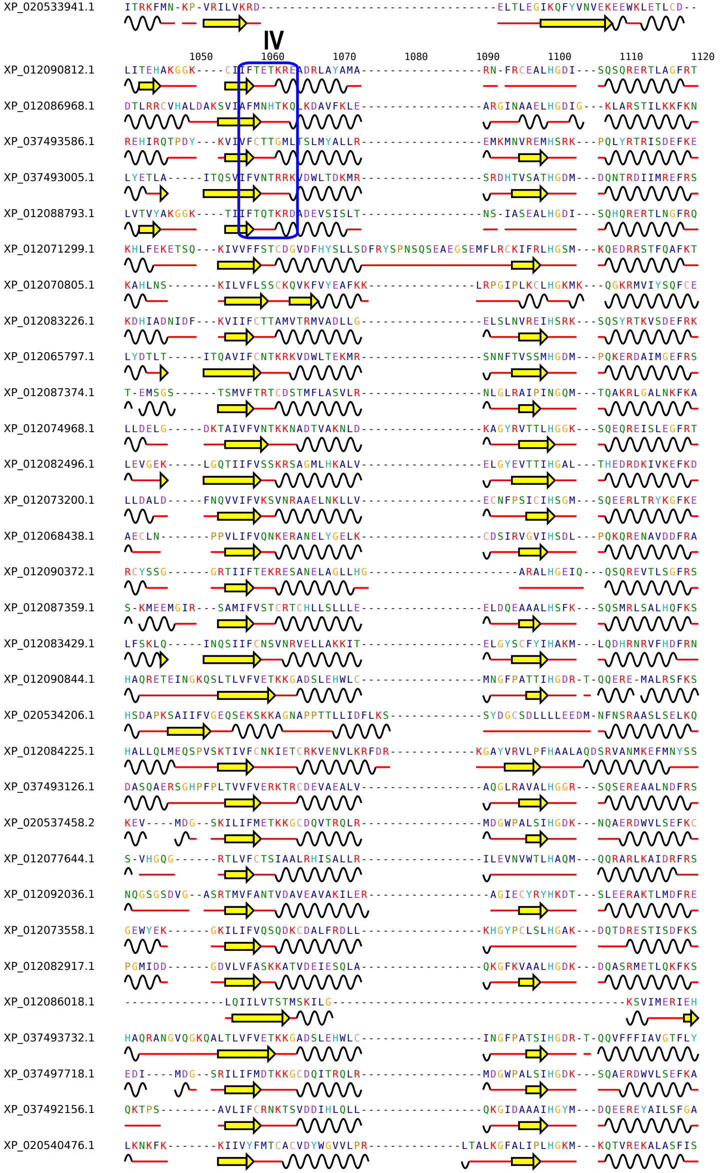
XP_020533941.1 ITRKFMN-KP-VRILVKRD---------------------------------ELTLEGIKQFYVNVEKEEWKLETLCD--
IV
1050 1060 1070 1080 1090 1100 1110 1120
XP_012090812.1 LITEHAKGGK----CIIFTETKREADRLAYAMA-----------------RN-FRCEALHGDI---SQSQRERTLAGFRT
XP_012086968.1 DTLRRCVHALDAKSVIAFMNHTKQLKDAVFKLE-----------------ARGINAAELHGDIG---KLARSTILKKFKN
XP_037493586.1 REHIRQTPDY---KVIVFCTTGMLTSLMYALLR-----------------EMKMNVREMHSRK---PQLYRTRISDEFKE
XP_037493005.1 LYETLA-----ITQSVIFVNTRRKVDWLTDKMR-----------------SRDHTVSATHGDM---DQNTRDIIMREFRS
XP_012088793.1 LVTVYAKGGK----TIIFTQTKRDADEVSISLT-----------------NS-IASEALHGDI---SQHQRERTLNGFRQ
XP_012071299.1 KHLFEKETSQ---KIVVFFSTCDGVDFHYSLLSDFRYSPNSQSEAEGSEMFLRCKIFRLHGSM---KQEDRRSTFQAFKT
XP_012070805.1 KAHLNS-------KILVFLSSCKQVKFVYEAFKK---------------LRPGIPLKCLHGKMK---QGKRMVIYSQFCE
XP_012083226.1 KDHIADNIDF---KVIIFCTTAMVTRMVADLLG-----------------ELSLNVREIHSRK---SQSYRTKVSDEFRK
XP_012065797.1 LYDTLT-----ITQAVIFCNTKRKVDWLTEKMR-----------------SNNFTVSSMHGDM---PQKERDAIMGEFRS
XP_012087374.1 T-EMSGS------TSMVFTRTCDSTMFLASVLR-----------------NLGLRAIPINGQM---TQAKRLGALNKFKA
XP_012074968.1 LLDELG-----DKTAIVFVNTKKNADTVAKNLD-----------------KAGYRVTTLHGGK---SQEQREISLEGFRT
XP_012082496.1 LEVGEK-----LGQTIIFVSSKRSAGMLHKALV-----------------ELGYEVTTIHGAL---THEDRDKIVKEFKD
XP_012073200.1 LLDALD-----FNQVVIFVKSVNRAAELNKLLV-----------------ECNFPSICIHSGM---SQEERLTRYKGFKE
XP_012068438.1 AECLN-------PPVLIFVQNKERANELYGELK-----------------CDSIRVGVIHSDL---PQKQRENAVDDFRA
XP_012090372.1 RCYSSG------GRTIIFTEKRESANELAGLLHG---------------------ARALHGEIQ---QSQREVTLSGFRS
XP_012087359.1 S-KMEEMGIR---SAMIFVSTCRTCHLLSLLLE-----------------ELDQEAAALHSFK---SQSMRLSALHQFKS
XP_012083429.1 LFSKLQ-----INQSIIFCNSVNRVELLAKKIT-----------------ELGYSCFYIHAKM---LQDHRNRVFHDFRN
XP_012090844.1 HAQRETEINGKQSLTLVFVETKKGADSLEHWLC-----------------MNGFPATTIHGDR-T-QQERE-MALRSFKS
XP_020534206.1 HSDAPKSAIIFVGEQSEKSKKAGNAPPTTLLIDFLKS-------------SYDGCSDLLLLEEDM-NFNSRAASLSELKQ
XP_012084225.1 HALLQLMEQSPVSKTIVFCNKIETCRKVENVLKRFDR------------KGAYVRVLPFHAALAQDSRVANMKEFMNYSS
XP_037493126.1 DASQAERSGHPFPLTVVFVERKTRCDEVAEALV-----------------AQGLRAVALHGGR---SQSEREAALNDFRS
XP_020537458.2 KEV----MDG--SKILIFMETKKGCDQVTRQLR-----------------MDGWPALSIHGDK---NQAERDWVLSEFKC
XP_012077644.1 S-VHGQG------RTLVFCTSIAALRHISALLR-----------------ILEVNVWTLHAQM---QQRARLKAIDRFRS
XP_012092036.1 NQGSGSDVG--ASRTMVFANTVDAVEAVAKILER-----------------AGIECYRYHKDT---SLEERAKTLMDFRE
XP_012073558.1 GEWYEK------GKILIFVQSQDKCDALFRDLL-----------------KHGYPCLSLHGAK---DQTDRESTISDFKS
XP_012082917.1 PGMIDD------GDVLVFASKKATVDEIESQLA-----------------QKGFKVAALHGDK---DQASRMETLQKFKS
XP_012086018.1 --------------LQIILVTSTMSKILG---KSVIMERIEH
XP_037493732.1 HAQRANGVQGKQALTLVFVETKKGADSLEHWLC-----------------INGFPATSIHGDR-T-QQVFFFIAVGTFLY
XP_037497718.1 EDI----MDG--SRILIFMDTKKGCDQITRQLR-----------------MDGWPALSIHGDK---SQAERDWVLSEFKA
XP_037492156.1 QKTPS--------AVLIFCRNKTSVDDIHLQLL-----------------QKGIDAAAIHGYM---DQEEREYAILSFGA
XP_020540476.1 LKNKFK-------KIIVYFMTCACVDYWGVVLPR-------------LTALKGFALIPLHGKM---KQTVREKALASFIS

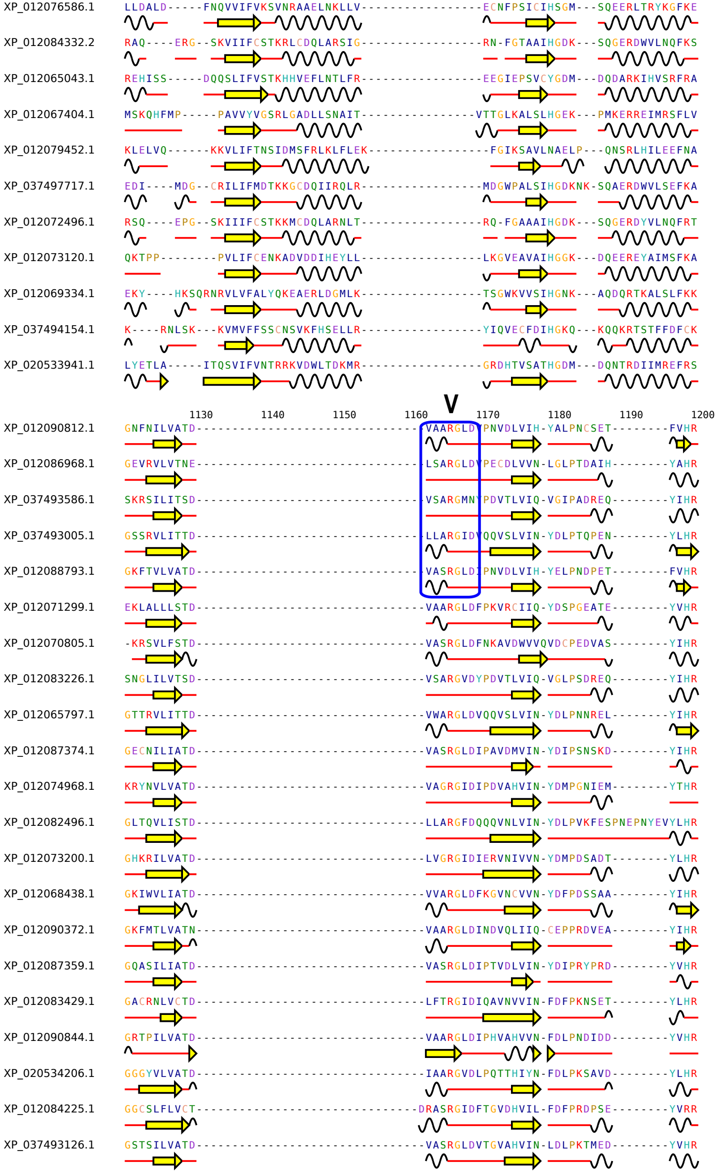

XP_012076586.1 LLDALD-----FNQVVIFVKSVNRAAELNKLLV----------------ECNFPSICIHSGM---SQEERLTRYKGFKE
XP_012084332.2 RAQ----ERG--SKVIIFCSTKRLCDQLARSIG-----------------RN-FGTAAIHGDK---SQGERDWVLNQFKS
XP_012065043.1 REHISS-----DQQSLIFVSTKHHVEFLNTLFR-----------------EEGIEPSVCYGDM---DQDARKIHVSRFRA
XP_012067404.1 MSKQHFMP-----PAVVYVGSRLGADLLSNAIT----------------VTTGLKALSLHGEK---PMKERREIMRSFLV
XP_012079452.1 KLELVQ------KKVLIFTNSIDMSFRLKLFLEK-----------------FGIKSAVLNAELP---QNSRLHILEEFNA
XP_037497717.1 EDI----MDG--CRILIFMDTKKGCDQIIRQLR-----------------MDGWPALSIHGDKNK-SQAERDWVLSEFKA
XP_012072496.1 RSQ----EPG--SKIIIFCSTKKMCDQLARNLT-----------------RQ-FGAAAIHGDK---SQGERDYVLNQFRT
XP_012073120.1 QKTPP--------PVLIFCENKADVDDIHEYLL-----------------LKGVEAVAIHGGK---DQEEREYAIMSFKA
XP_012069334.1 EKY----HKSQRNRVLVFALYQKEAERLDGMLK-----------------TSGWKVVSIHGNK---AQDQRTKALSLFKK
XP_037494154.1 K----RNLSK---KVMVFFSSCNSVKFHSELLR-----------------YIQVECFDIHGKQ---KQQKRTSTFFDFCK
XP_020533941.1 LYETLA-----ITQSVIFVNTRRKVDWLTDKMR-----------------GRDHTVSATHGDM---DQNTRDIIMREFRS
V
1130 1140 1150 1160 1170 1180 1190 1200
XP_012090812.1 GNFNILVATD--------------------------------VAARGLDYPNVDLVIH-YALPNCSET--------FVHR
XP_012086968.1 GEVRVLVTNE--------------------------------LSARGLDYPECDLVVN-LGLPTDAIH--------YAHR
XP_037493586.1 SKRSILITSD--------------------------------VSARGMNYPDVTLVIQ-VGIPADREQ--------YIHR
XP_037493005.1 GSSRVLITTD--------------------------------LLARGIDYQQVSLVIN-YDLPTQPEN--------YLHR
XP_012088793.1 GKFTVLVATD--------------------------------VASRGLDIPNVDLVIH-YELPNDPET--------FVHR
XP_012071299.1 EKLALLLSTD--------------------------------VAARGLDFPKVRCIIQ-YDSPGEATE--------YVHR
XP_012070805.1 -KRSVLFSTD--------------------------------VASRGLDFNKAVDWVVQVDCPEDVAS--------YIHR
XP_012083226.1 SNGLILVTSD--------------------------------VSARGVDYPDVTLVIQ-VGLPSDREQ--------YIHR
XP_012065797.1 GTTRVLITTD--------------------------------VWARGLDVQQVSLVIN-YDLPNNREL--------YIHR
XP_012087374.1 GECNILIATD--------------------------------VASRGLDIPAVDMVIN-YDIPSNSKD--------YIHR
XP_012074968.1 KRYNVLVATD--------------------------------VAGRGIDIPDVAHVIN-YDMPGNIEM--------YTHR
XP_012082496.1 GLTQVLISTD--------------------------------LLARGFDQQQVNLVIN-YDLPVKFESPNEPNYEVYLHR
XP_012073200.1 GHKRILVATD--------------------------------LVGRGIDIERVNIVVN-YDMPDSADT--------YLHR
XP_012068438.1 GKIWVLIATD--------------------------------VVARGLDFKGVNCVVN-YDFPDSSAA--------YIHR
XP_012090372.1 GKFMTLVATN--------------------------------VAARGLDINDVQLIIQ-CEPPRDVEA--------YIHR
XP_012087359.1 GQASILIATD--------------------------------VASRGLDIPTVDLVIN-YDIPRYPRD--------YVHR
XP_012083429.1 GACRNLVCTD--------------------------------LFTRGIDIQAVNVVIN-FDFPKNSET--------YLHR
XP_012090844.1 GRTPILVATD--------------------------------VAARGLDIPHVAHVVN-FDLPNDIDD--------YVHR
XP_020534206.1 GGGYVLVATD--------------------------------IAARGVDLPQTTHIYN-FDLPKSAVD--------YLHR
XP_012084225.1 GGCSLFLVCT-------------------------------DRASRGIDFTGVDHVIL-FDFPRDPSE--------YVRR
XP_037493126.1 GSTSILVATD--------------------------------VASRGLDVTGVAHVIN-LDLPKTMED--------YVHR

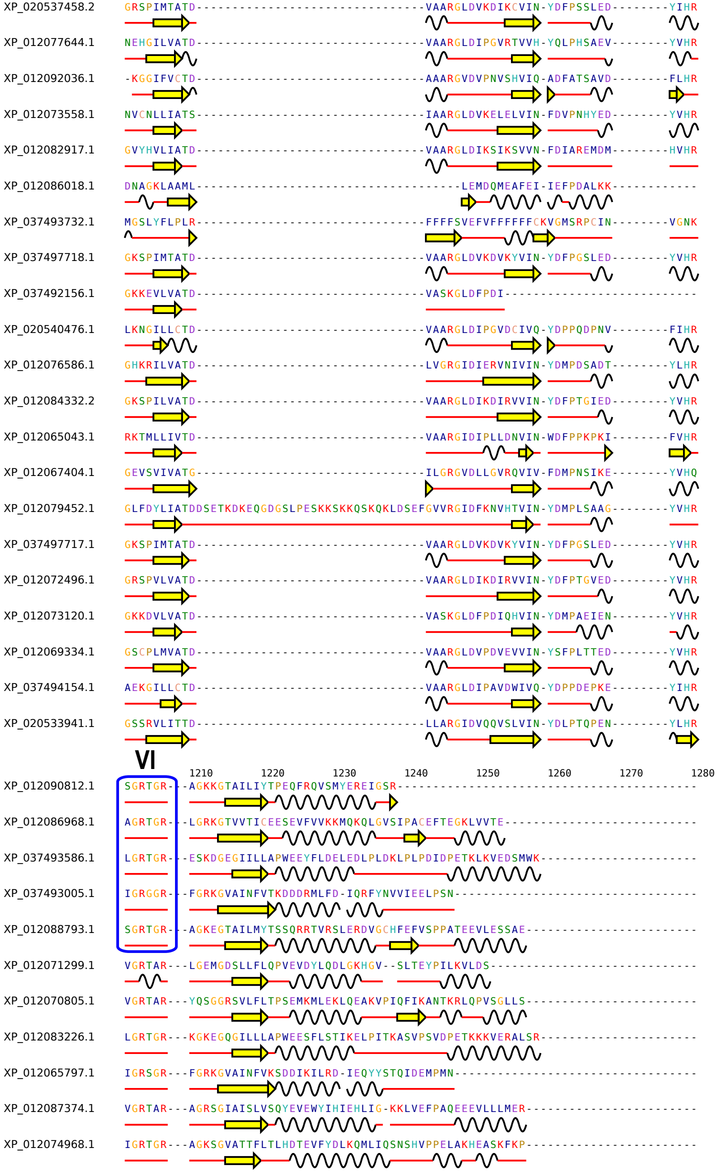
XP_020537458.2 GRSPIMTATD--------------------------------VAARGLDVKDIKCVIN-YDFPSSLED--------YIHR
XP_012077644.1 NEHGILVATD--------------------------------VAARGLDIPGVRTVVH-YQLPHSAEV--------YVHR
XP_012092036.1 -KGGIFVCTD--------------------------------AAARGVDVPNVSHVIQ-ADFATSAVD--------FLHR
XP_012073558.1 NVCNLLIATS--------------------------------IAARGLDVKELELVIN-FDVPNHYED--------YVHR
XP_012082917.1 GVYHVLIATD--------------------------------VAARGLDIKSIKSVVN-FDIAREMDM--------HVHR
XP_012086018.1 DNAGKLAAML-------------------------------------LEMDQMEAFEI-IEFPDALKK------------
XP_037493732.1 MGSLYFLPLR--------------------------------FFFFSVEFVFFFFFFCKVGMSRPCIN--------VGNK
XP_037497718.1 GKSPIMTATD--------------------------------VAARGLDVKDVKYVIN-YDFPGSLED--------YVHR
XP_037492156.1 GKKEVLVATD--------------------------------VASKGLDFPDI---------------------------
XP_020540476.1 LKNGILLCTD--------------------------------VAARGLDIPGVDCIVQ-YDPPQDPNV--------FIHR
XP_012076586.1 GHKRILVATD--------------------------------LVGRGIDIERVNIVIN-YDMPDSADT--------YLHR
XP_012084332.2 GKSPILVATD--------------------------------VAARGLDIKDIRVVIN-YDFPTGIED--------YVHR
XP_012065043.1 RKTMLLIVTD--------------------------------VAARGIDIPLLDNVIN-WDFPPKPKI--------FVHR
XP_012067404.1 GEVSVIVATG--------------------------------ILGRGVDLLGVRQVIV-FDMPNSIKE--------YVHQ
XP_012079452.1 GLFDYLIATDDSETKDKEQGDGSLPESKKSKKQSKQKLDSEFGVVRGIDFKNVHTVIN-YDMPLSAAG--------YVHR
XP_037497717.1 GKSPIMTATD--------------------------------VAARGLDVKDVKYVIN-YDFPGSLED--------YVHR
XP_012072496.1 GRSPVLVATD--------------------------------VAARGLDIKDIRVVIN-YDFPTGVED--------YVHR
XP_012073120.1 GKKDVLVATD--------------------------------VASKGLDFPDIQHVIN-YDMPAEIEN--------YVHR
XP_012069334.1 GSCPLMVATD--------------------------------VAARGLDVPDVEVVIN-YSFPLTTED--------YVHR
XP_037494154.1 AEKGILLCTD--------------------------------VAARGLDIPAVDWIVQ-YDPPDEPKE--------YIHR
XP_020533941.1 GSSRVLITTD--------------------------------LLARGIDVQQVSLVIN-YDLPTQPEN--------YLHR
VI
1210 1220 1230 1240 1250 1260 1270 1280
XP_012090812.1 SGRTGR---AGKKGTAILIYTPEQFRQVSMYEREIGSR--
XP_012086968.1 AGRTGR---LGRKGTVVTICEESEVFVVKKMQKQLGVSIPACEFTEGKLVVTE---------------------------
XP_037493586.1 LGRTGR---ESKDGEGIILLAPWEEYFLDELEDLPLDKLPLPDIDPETKLKVEDSMWK----------------------
XP_037493005.1 IGRGGR---FGRKGVAINFVTKDDDRMLFD-IQRFYNVVIEELPSN----------------------------------
XP_012088793.1 SGRTGR---AGKEGTAILMYTSSQRRTVRSLERDVGCHFEFVSPPATEEVLESSAE------------------------
XP_012071299.1 VGRTAR---LGEMGDSLLFLQPVEVDYLQDLGKHGV--SLTEYPILKVLDS-----------------------------
XP_012070805.1 VGRTAR---YQSGGRSVLFLTPSEMKMLEKLQEAKVPIQFIKANTKRLQPVSGLLS------------------------
XP_012083226.1 LGRTGR---KGKEGQGILLLAPWEESFLSTIKELPITKASVPSVDPETKKKVERALSR----------------------
XP_012065797.1 IGRSGR---FGRKGVAINFVKSDDIKILRD-IEQYYSTQIDEMPMN----------------------------------
XP_012087374.1 VGRTAR---AGRSGIAISLVSQYEVEWYIHIEHLIG-KKLVEFPAQEEEVLLLMER------------------------
XP_012074968.1 IGRTGR---AGKSGVATTFLTLHDTEVFYDLKQMLIQSNSHVPPELAKHEASKFKP------------------------

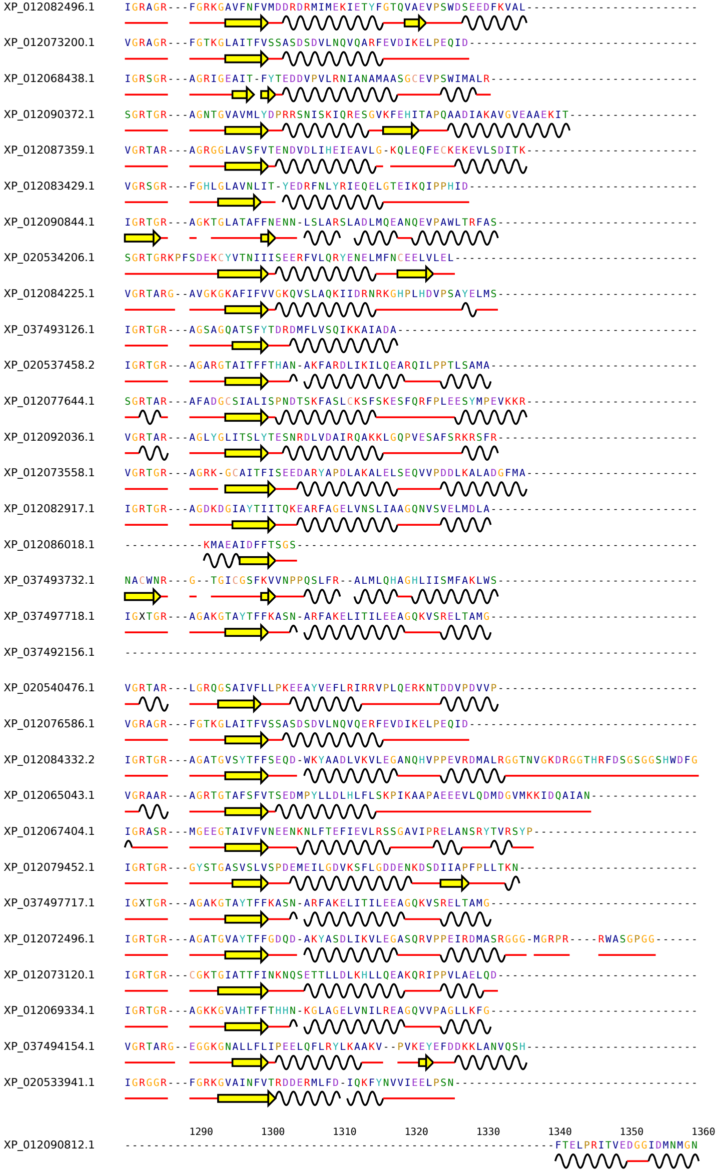

XP_012082496.1 IGRAGR---FGRKGAVFNFVMDDRDRMIMEKIETYFGTQVAEVPSWDSEEDFKVAL------------------------
XP_012073200.1 VGRAGR---FGTKGLAITFVSSASDSDVLNQVQARFEVDIKELPEQID--------------------------------
XP_012068438.1 IGRSGR---AGRIGEAIT-FYTEDDVPVLRNIANAMAASGCEVPSWIMALR-----------------------------
XP_012090372.1 SGRTGR---AGNTGVAVMLYDPRRSNISKIQRESGVKFEHITAPQAADIAKAVGVEAAEKIT------------------
XP_012087359.1 VGRTAR---AGRGGLAVSFVTENDVDLIHEIEAVLG-KQLEQFECKEKEVLSDITK------------------------
XP_012083429.1 VGRSGR---FGHLGLAVNLIT-YEDRFNLYRIEQELGTEIKQIPPHID--------------------------------
XP_012090844.1 IGRTGR---AGKTGLATAFFNENN-LSLARSLADLMQEANQEVPAWLTRFAS----------------------------
XP_020534206.1 SGRTGRKPFSDEKCYVTNIIISEERFVLQRYENELMFNCEELVLEL----------------------------------
XP_012084225.1 VGRTARG--AVGKGKAFIFVVGKQVSLAQKIIDRNRKGHPLHDVPSAYELMS----------------------------
XP_037493126.1 IGRTGR---AGSAGQATSFYTDRDMFLVSQIKKAIADA--
XP_020537458.2 IGRTGR---AGARGTAITFFTHAN-AKFARDLIKILQEARQILPPTLSAMA-----------------------------
XP_012077644.1 SGRTAR---AFADGCSIALISPNDTSKFASLCKSFSKESFQRFPLEESYMPEVKKR------------------------
XP_012092036.1 VGRTAR---AGLYGLITSLYTESNRDLVDAIRQAKKLGQPVESAFSRKRSFR----------------------------
XP_012073558.1 VGRTGR---AGRK-GCAITFISEEDARYAPDLAKALELSEQVVPDDLKALADGFMA------------------------
XP_012082917.1 IGRTGR---AGDKDGIAYTIITQKEARFAGELVNSLIAAGQNVSVELMDLA-----------------------------
XP_012086018.1 -----------KMAEAIDFFTSGS--
XP_037493732.1 NACWNR---G--TGICGSFKVVNPPQSLFR--ALMLQHAGHLIISMFAKLWS----------------------------
XP_037497718.1 IGXTGR---AGAKGTAYTFFKASN-ARFAKELITILEEAGQKVSRELTAMG-----------------------------
XP_037492156.1 --
XP_020540476.1 VGRTAR---LGRQGSAIVFLLPKEEAYVEFLRIRRVPLQERKNTDDVPDVVP----------------------------
XP_012076586.1 VGRAGR---FGTKGLAITFVSSASDSDVLNQVQERFEVDIKELPEQID--------------------------------
XP_012084332.2 IGRTGR---AGATGVSYTFFSEQD-WKYAADLVKVLEGANQHVPPEVRDMALRGGTNVGKDRGGTHRFDSGSGGSHWDFG
XP_012065043.1 VGRAAR---AGRTGTAFSFVTSEDMPYLLDLHLFLSKPIKAAPAEEEVLQDMDGVMKKIDQAIAN---------------
XP_012067404.1 IGRASR---MGEEGTAIVFVNEENKNLFTEFIEVLRSSGAVIPRELANSRYTVRSYP-----------------------
XP_012079452.1 IGRTGR---GYSTGASVSLVSPDEMEILGDVKSFLGDDENKDSDIIAPFPLLTKN-------------------------
XP_037497717.1 IGXTGR---AGAKGTAYTFFKASN-ARFAKELITILEEAGQKVSRELTAMG-----------------------------
XP_012072496.1 IGRTGR---AGATGVAYTFFGDQD-AKYASDLIKVLEGASQRVPPEIRDMASRGGG-MGRPR---RWASGPGG-------
XP_012073120.1 IGRTGR---CGKTGIATTFINKNQSETTLLDLKHLLQEAKQRIPPVLAELQD----------------------------
XP_012069334.1 IGRTGR---AGKKGVAHTFFTHHN-KGLAGELVNILREAGQVVPAGLLKFG-----------------------------
XP_037494154.1 VGRTARG--EGGKGNALLFLIPEELQFLRYLKAAKV--PVKEYEFDDKKLANVQSH------------------------
XP_020533941.1 IGRGGR---FGRKGVAINFVTRDDERMLFD-IQKFYNVVIEELPSN----------------------------------
1290 1300 1310 1320 1330 1340 1350 1360
XP_012090812.1 --FTELPRITVEDGGIDMNMGN

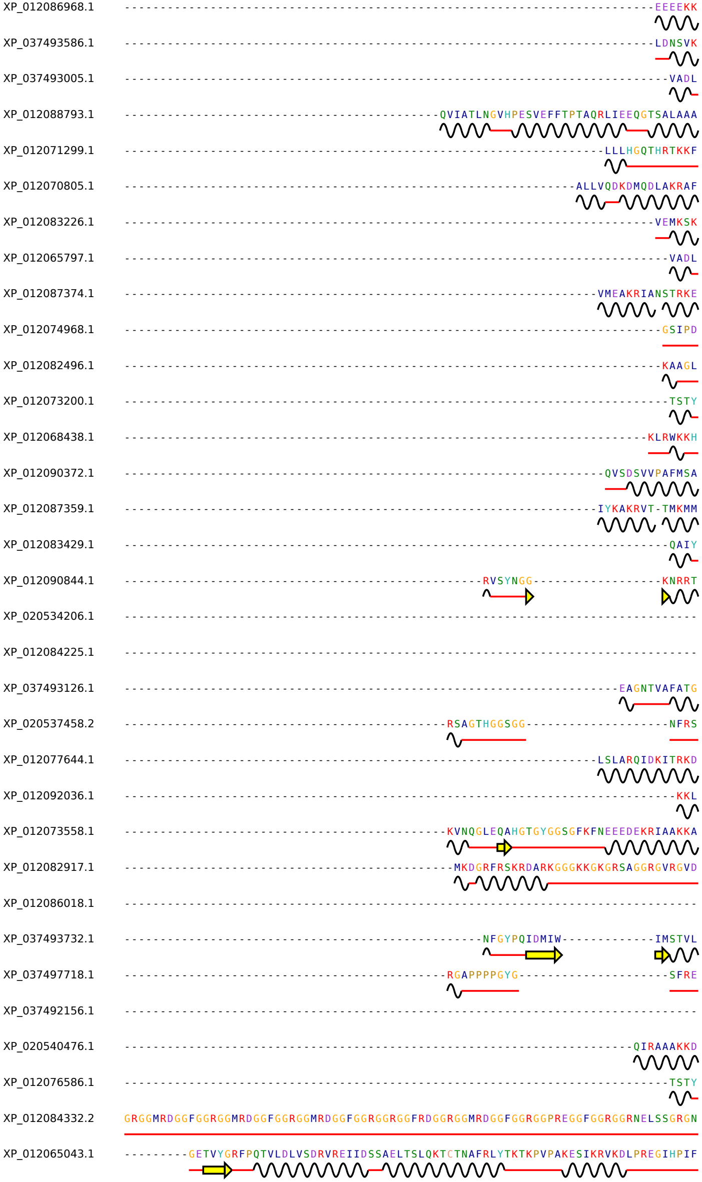
XP_012086968.1 EEEEKK
XP_037493586.1 LDNSVK
XP_037493005.1 VADL
XP_012088793.1 QVIATLNGVHPESVEFFTPTAQRLIEEQGTSALAAA
XP_012071299.1 LLLHGQTHRTKKF
XP_012070805.1 ALLVQDKDMQDLAKRAF
XP_012083226.1 VEMKSK
XP_012065797.1 VADL
XP_012087374.1 VMEAKRIANSTRKE
XP_012074968.1 GSIPD
XP_012082496.1 KAAGL
XP_012073200.1 TSTY
XP_012068438.1 KLRWKKH
XP_012090372.1 QVSDSVVPAFMSA
XP_012087359.1 IYKAKRVT-TMKMM
XP_012083429.1 QAIY
XP_012090844.1 RVSYNGG KNRRT
XP_020534206.1
XP_012084225.1
XP_037493126.1 EAGNTVAFATG
XP_020537458.2 RSAGTHGGSGG NFRS
XP_012077644.1 LSLARQIDKITRKD
XP_012092036.1 KKL
XP_012073558.1 KVNQGLEQAHGTGYGGSGFKFNEEEDEKRIAAKKA
XP_012082917.1 MKDGRFRSKRDARKGGGKKGKGRSAGGRGVRGVD
XP_012086018.1
XP_037493732.1 NFGYPQIDMIW IMSTVL
XP_037497718.1 RGAPPPPGYG SFRE
XP_037492156.1
XP_020540476.1 QIRAAAKKD
XP_012076586.1 TSTY
XP_012084332.2 GRGGMRDGGFGGRGGMRDGGFGGRGGMRDGGFGGRGGRGGFRDGGRGGMRDGGFGGRGGPREGGFGGRGGRNELSSGRGN
XP_012065043.1 GETVYGRFPQTVLDLVSDRVREIIDSSAELTSLQKTCTNAFRLYTKTKPVPAKESIKRVKDLPREGIHPIF

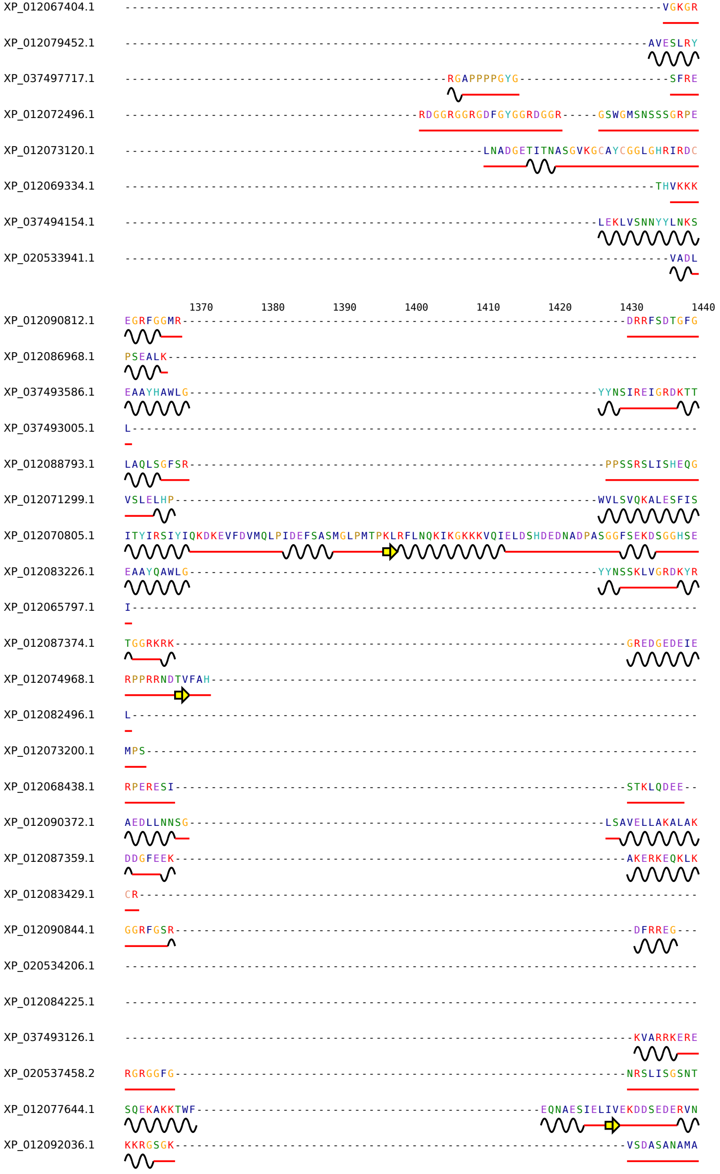
XP_012067404.1 VGKGR
XP_012079452.1 AVESLRY
XP_037497717.1 RGAPPPPGYG SFRE
XP_012072496.1 RDGGRGGRGDFGYGGRDGGR GSWGMSNSSSGRPE
XP_012073120.1 LNADGETITNASGVKGCAYCGGLGHRIRDC
XP_012069334.1 THVKKK
XP_037494154.1 LEKLVSNNYYLNKS
XP_020533941.1 VADL
1370 1380 1390 1400 1410 1420 1430 1440
XP_012090812.1 EGRFGGMR DRRFSDTGFG
XP_012086968.1 PSEALK
XP_037493586.1 EAAYHAWLG YYNSIREIGRDKTT
XP_037493005.1 L
XP_012088793.1 LAQLSGFSR PPSSRSLISHEQG
XP_012071299.1 VSLELHP WVLSVQKALESFIS
XP_012070805.1 ITYIRSIYIQKDKEVFDVMQLPIDEFSASMGLPMTPKLRFLNQKIKGKKKVQIELDSHDEDNADPASGGFSEKDSGGHSE
XP_012083226.1 EAAYQAWLG YYNSSKLVGRDKYR
XP_012065797.1 I
XP_012087374.1 TGGRKRK GREDGEDEIE
XP_012074968.1 RPPRRNDTVFAH
XP_012082496.1 L
XP_012073200.1 MPS
XP_012068438.1 RPERESI STKLQDEE
XP_012090372.1 AEDLLNNSG LSAVELLAKALAK
XP_012087359.1 DDGFEEK AKERKEQKLK
XP_012083429.1 CR
XP_012090844.1 GGRFGSR DFRREG
XP_020534206.1
XP_012084225.1
XP_037493126.1 KVARRKERE
XP_020537458.2 RGRGGFG NRSLISGSNT
XP_012077644.1 SQEKAKKTWF EQNAESIELIVEKDDSEDERVN
XP_012092036.1 KKRGSGK VSDASANAMA

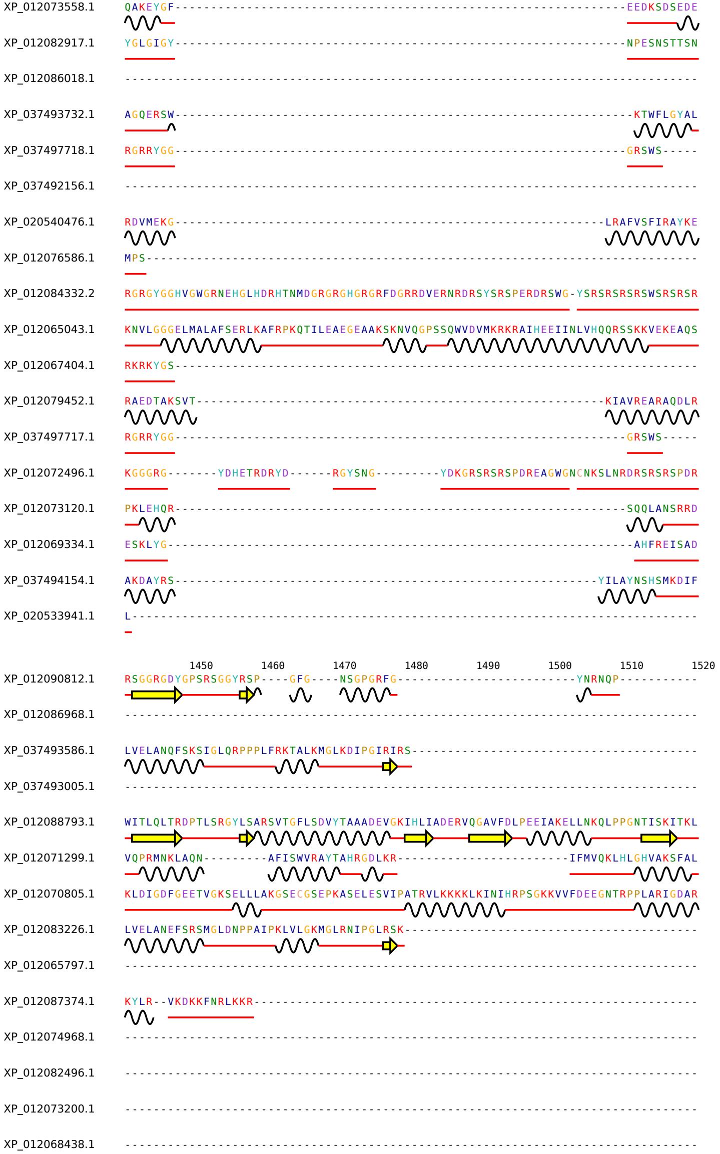
XP_012073558.1 QAKEYGF---EEDKSDSEDE
XP_012082917.1 YGLGIGY---NPESNSTTSN
XP_012086018.1 --
XP_037493732.1 AGQERSW--KTWFLGYAL
XP_037497718.1 RGRRYGG---GRSWS-----
XP_037492156.1 --
XP_020540476.1 RDVMEKG--LRAFVSFIRAYKE
XP_012076586.1 MPS---
XP_012084332.2 RGRGYGGHVGWGRNEHGLHDRHTNMDGRGRGHGRGRFDGRRDVERNRDRSYSRSPERDRSWG-YSRSRSRSRSWSRSRSR
XP_012065043.1 KNVLGGGELMALAFSERLKAFRPKQTILEAEGEAAKSKNVQGPSSQWVDVMKRKRAIHEEIINLVHQQRSSKKVEKEAQS
XP_012067404.1 RKRKYGS---
XP_012079452.1 RAEDTAKSVT---KIAVREARAQDLR
XP_037497717.1 RGRRYGG---GRSWS-----
XP_012072496.1 KGGGRG-------YDHETRDRYD------RGYSNG---------YDKGRSRSRSPDREAGWGNCNKSLNRDRSRSRSPDR
XP_012073120.1 PKLEHQR---SQQLANSRRD
XP_012069334.1 ESKLYG---AHFREISAD
XP_037494154.1 AKDAYRS---YILAYNSHSMKDIF
XP_020533941.1 L---
1450 1460 1470 1480 1490 1500 1510 1520
XP_012090812.1 RSGGRGDYGPSRSGGYRSP----GFG----NSGPGRFG-------------------------YNRNQP-----------
XP_012086968.1 --
XP_037493586.1 LVELANQFSKSIGLQRPPPLFRKTALKMGLKDIPGIRIRS--
XP_037493005.1 --
XP_012088793.1 WITLQLTRDPTLSRGYLSARSVTGFLSDVYTAAADEVGKIHLIADERVQGAVFDLPEEIAKELLNKQLPPGNTISKITKL
XP_012071299.1 VQPRMNKLAQN---------AFISWVRAYTAHRGDLKR------------------------IFMVQKLHLGHVAKSFAL
XP_012070805.1 KLDIGDFGEETVGKSELLLAKGSECGSEPKASELESVIPATRVLKKKKLKINIHRPSGKKVVFDEEGNTRPPLARIGDAR
XP_012083226.1 LVELANEFSRSMGLDNPPAIPKLVLGKMGLRNIPGLRSK---
XP_012065797.1 --
XP_012087374.1 KYLR--VKDKKFNRLKKR--
XP_012074968.1 --
XP_012082496.1 --
XP_012073200.1 --
XP_012068438.1 --

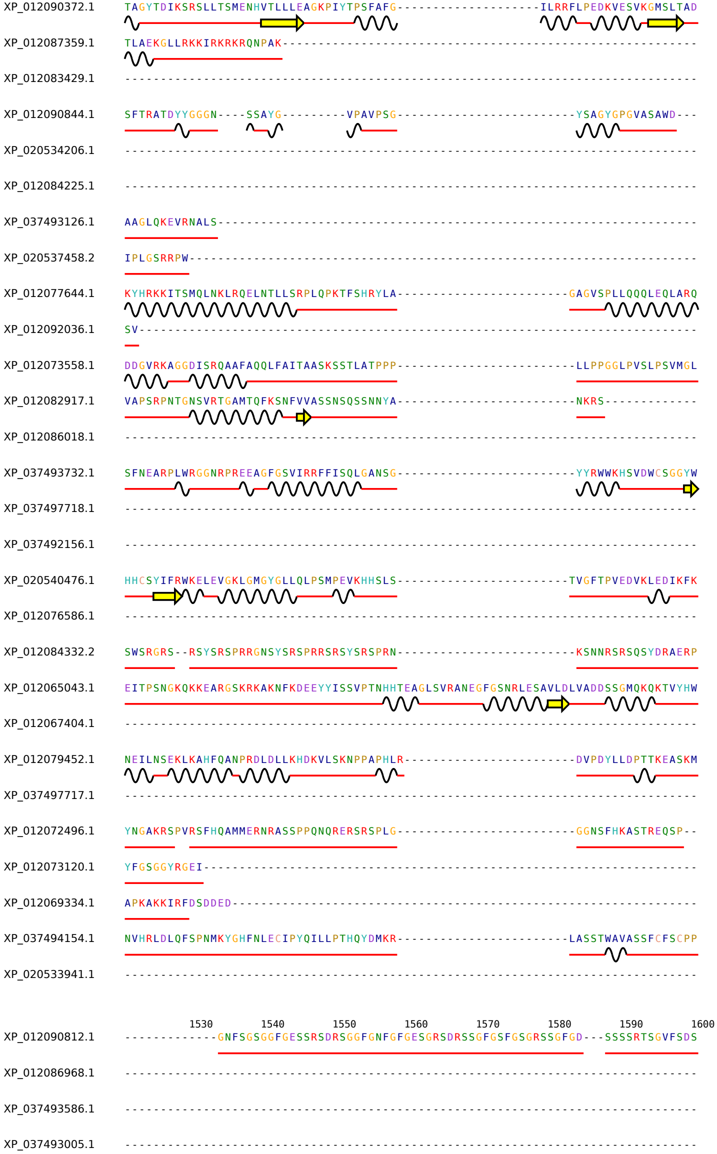
XP_012090372.1 TAGYTDIKSRSLLTSMENHVTLLLEAGKPIYTPSFAFG--------------------ILRRFLPEDKVESVKGMSLTAD
XP_012087359.1 TLAEKGLLRKKIRKRKRQNPAK--
XP_012083429.1 --
XP_012090844.1 SFTRATDYYGGGN----SSAYG---------VPAVPSG-------------------------YSAGYGPGVASAWD---
XP_020534206.1 --
XP_012084225.1 --
XP_037493126.1 AAGLQKEVRNALS---
XP_020537458.2 IPLGSRRPW---
XP_012077644.1 KYHRKKITSMQLNKLRQELNTLLSRPLQPKTFSHRYLA------------------------GAGVSPLLQQQLEQLARQ
XP_012092036.1 SV--
XP_012073558.1 DDGVRKAGGDISRQAAFAQQLFAITAASKSSTLATPPP-------------------------LLPPGGLPVSLPSVMGL
XP_012082917.1 VAPSRPNTGNSVRTGAMTQFKSNFVVASSNSQSSNNYA-------------------------NKRS-------------
XP_012086018.1 --
XP_037493732.1 SFNEARPLWRGGNRPREEAGFGSVIRRFFISQLGANSG-------------------------YYRWWKHSVDWCSGGYW
XP_037497718.1 --
XP_037492156.1 --
XP_020540476.1 HHCSYIFRWKELEVGKLGMGYGLLQLPSMPEVKHHSLS------------------------TVGFTPVEDVKLEDIKFK
XP_012076586.1 --
XP_012084332.2 SWSRGRS--RSYSRSPRRGNSYSRSPRRSRSYSRSPRN-------------------------KSNNRSRSQSYDRAERP
XP_012065043.1 EITPSNGKQKKEARGSKRKAKNFKDEEYYISSVPTNHHTEAGLSVRANEGFGSNRLESAVLDLVADDSSGMQKQKTVYHW
XP_012067404.1 --
XP_012079452.1 NEILNSEKLKAHFQANPRDLDLLKHDKVLSKNPPAPHLR------------------------DVPDYLLDPTTKEASKM
XP_037497717.1 --
XP_012072496.1 YNGAKRSPVRSFHQAMMERNRASSPPQNQRERSRSPLG-------------------------GGNSFHKASTREQSP--
XP_012073120.1 YFGSGGYRGEI---
XP_012069334.1 APKAKKIRFDSDDED---
XP_037494154.1 NVHRLDLQFSPNMKYGHFNLECIPYQILLPTHQYDMKR------------------------LASSTWAVASSFCFSCPP
XP_020533941.1 --
1530 1540 1550 1560 1570 1580 1590 1600
XP_012090812.1 -------------GNFSGSGGFGESSRSDRSGGFGNFGFGESGRSDRSSGFGSFGSGRSSGFGD---SSSSRTSGVFSDS
XP_012086968.1 --
XP_037493586.1 --
XP_037493005.1 --

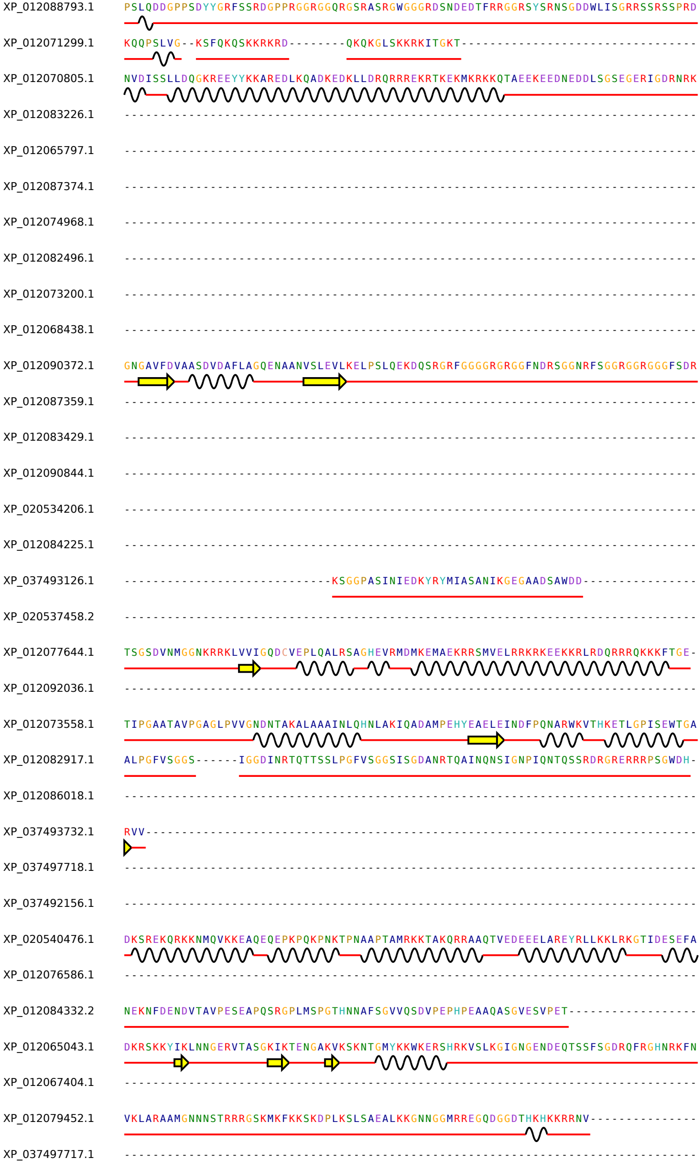
XP_012088793.1 PSLQDDGPPSDYYGRFSSRDGPPRGGRGGQRGSRASRGWGGGRDSNDEDTFRRGGRSYSRNSGDDWLISGRRSSRSSPRD
XP_012071299.1 KQQPSLVG--KSFQKQSKKRKRD--------QKQKGLSKKRKITGKT---------------------------------
XP_012070805.1 NVDISSLLDQGKREEYYKKAREDLKQADKEDKLLDRQRRREKRTKEKMKRKKQTAEEKEEDNEDDLSGSEGERIGDRNRK
XP_012083226.1 --
XP_012065797.1 --
XP_012087374.1 --
XP_012074968.1 --
XP_012082496.1 --
XP_012073200.1 --
XP_012068438.1 --
XP_012090372.1 GNGAVFDVAASDVDAFLAGQENAANVSLEVLKELPSLQEKDQSRGRFGGGGRGRGGFNDRSGGNRFSGGRGGRGGGFSDR
XP_012087359.1 --
XP_012083429.1 --
XP_012090844.1 --
XP_020534206.1 --
XP_012084225.1 --
XP_037493126.1 -----------------------------KSGGPASINIEDKYRYMIASANIKGEGAADSAWDD----------------
XP_020537458.2 --
XP_012077644.1 TSGSDVNMGGNKRRKLVVIGQDCVEPLQALRSAGHEVRMDMKEMAEKRRSMVELRRKRKEEKKRLRDQRRRQKKKFTGE-
XP_012092036.1 --
XP_012073558.1 TIPGAATAVPGAGLPVVGNDNTAKALAAAINLQHNLAKIQADAMPEHYEAELEINDFPQNARWKVTHKETLGPISEWTGA
XP_012082917.1 ALPGFVSGGS------IGGDINRTQTTSSLPGFVSGGSISGDANRTQAINQNSIGNPIQNTQSSRDRGERRRPSGWDH-
XP_012086018.1 --
XP_037493732.1 RVV---
XP_037497718.1 --
XP_037492156.1 --
XP_020540476.1 DKSREKQRKKNMQVKKEAQEQEPKPQKPNKTPNAAPTAMRKKTAKQRRAAQTVEDEEELAREYRLLKKLRKGTIDESEFA
XP_012076586.1 --
XP_012084332.2 NEKNFDENDVTAVPESEAPQSRGPLMSPGTHNNAFSGVVQSDVPEPHPEAAQASGVESVPET------------------
XP_012065043.1 DKRSKKYIKLNNGERVTASGKIKTENGAKVKSKNTGMYKKWKERSHRKVSLKGIGNGENDEQTSSFSGDRQFRGHNRKFN
XP_012067404.1 --
XP_012079452.1 VKLARAAMGNNNSTRRRGSKMKFKKSKDPLKSLSAEALKKGNNGGMRREGQDGGDTHKHKKRRNV---------------
XP_037497717.1 --

```
XP_012072496.1    -AGSFHQ-AIMGRGQGSTLTEQGRSPATGQENTGYNGGSYSHFGEDEEEGMIPADNEGMSQRADGH--------------
XP_012073120.1    --------------------------------------------------------------------------------
XP_012069334.1    --------------------------------------------------------------------------------
XP_037494154.1    KVNLNIDSSASKFRKKAHKGSRNGFSESNPYGRQRDEEDKRQFVRH----------------------------------
XP_020533941.1    --------------------------------------------------------------------------------

                            1610          1620          1630          1640          1650          1660
XP_012090812.1    RSSRFG---SFGDVKTDDFNTG-----------KRQF------------------------------
XP_012086968.1    -------------------------------------------------------------------
XP_037493586.1    -------------------------------------------------------------------
XP_037493005.1    -------------------------------------------------------------------
XP_012088793.1    RSSSWGRSSSQDRFGGSCFNCGRPGHRASDCPNKADF------------------------------
XP_012071299.1    -------------------------------------------------------------------
XP_012070805.1    RTKIYFDSDNDDGNAVGKRENLVNTDSISLAEQEALALKLLNSM-----------------------
XP_012083226.1    -------------------------------------------------------------------
XP_012065797.1    -------------------------------------------------------------------
XP_012087374.1    -------------------------------------------------------------------
XP_012074968.1    -------------------------------------------------------------------
XP_012082496.1    -------------------------------------------------------------------
XP_012073200.1    -------------------------------------------------------------------
XP_012068438.1    -------------------------------------------------------------------
XP_012090372.1    RNSFGGRGRSNGNRW----------------------------------------------------
XP_012087359.1    -------------------------------------------------------------------
XP_012083429.1    -------------------------------------------------------------------
XP_012090844.1    -------------------------------------------------------------------
XP_020534206.1    -------------------------------------------------------------------
XP_012084225.1    -------------------------------------------------------------------
XP_037493126.1    -------------------------------------------------------------------
XP_020537458.2    -------------------------------------------------------------------
XP_012077644.1    -------------------------------------------------------------------
XP_012092036.1    -------------------------------------------------------------------
XP_012073558.1    AITTRGQYFPPGRIPGPGERKLYLFIEGPSETSVKKAKTELKRVLEDITNQALSLPGGAQPGRYSVI
XP_012082917.1    -------------------------------------------------------------------
XP_012086018.1    -------------------------------------------------------------------
```

```
XP_037493732.1    -------------------------------------------------------------------
XP_037497718.1    -------------------------------------------------------------------
XP_037492156.1    -------------------------------------------------------------------
XP_020540476.1    RLTGTEEL-----------------------------------------------------------
XP_012076586.1    -------------------------------------------------------------------
XP_012084332.2    -------------------------------------------------------------------
XP_012065043.1    AGRKHHSVPNANVRSEIKDLEQVRKERQKKANKISHMKNKAGGGKNFGKNKKRGKPRK---------
XP_012067404.1    -------------------------------------------------------------------
XP_012079452.1    -------------------------------------------------------------------
XP_037497717.1    -------------------------------------------------------------------
XP_012072496.1    -------------------------------------------------------------------
XP_012073120.1    -------------------------------------------------------------------
XP_012069334.1    -------------------------------------------------------------------
XP_037494154.1    -------------------------------------------------------------------
XP_020533941.1    -------------------------------------------------------------------
```
